# Integrative taxonomy of *Leptonetela* spiders (Araneae, Leptonetidae), with descriptions of 46 new species

**DOI:** 10.24272/j.issn.2095-8137.2017.076

**Published:** 2017-11-18

**Authors:** Chun-Xia Wang, Xin Xu, Shu-Qiang Li

**Affiliations:** ^1^Institute of Zoology, Chinese Academy of Sciences, Beijing 100101, China; ^2^Southeast Asia Biodiversity Research Institute, Chinese Academy of Sciences, Yezin Nay Pyi Taw 05282, Myanmar; ^3^College of Life Sciences, Hunan Normal University, Changsha Hunan 410006, China; ^4^University of Chinese Academy of Sciences, Beijing 100049, China

**Keywords:** DNA barcoding, Phylogeny, Phenotype, Species delineation

## Abstract

Extreme environments, such as subterranean habitats, are suspected to be responsible for morphologically inseparable cryptic or sibling species and can bias biodiversity assessment. A DNA barcode is a short, standardized DNA sequence used for taxonomic purposes and has the potential to lessen the challenges presented by a biotic inventory. Here, we investigate the diversity of the genus *Leptonetela* Kratochvíl, 1978 that is endemic to karst systems in Eurasia using DNA barcoding. We analyzed 624 specimens using one mitochondrial gene fragment (*COI*). The results show that DNA barcoding is an efficient and rapid species identification method in this genus. DNA barcoding gap and automatic barcode gap discovery (ABGD) analyses indicated the existence of 90 species, a result consistent with previous taxonomic hypotheses, and supported the existence of extreme male pedipalpal tibial spine and median apophysis polymorphism in *Leptonetela* species, with direct implications for the taxonomy of the group and its diversity. Based on the molecular and morphological evidence, we delimit and diagnose 90 *Leptonetela* species, including the type species *Leptonetela kanellisi*(Deeleman-Reinhold, 1971). Forty-six of them are previously undescribed. The female of *Leptonetela zhai* Wang & Li, 2011 is reported for the first time. *Leptonetela tianxinensis* (Tong & Li, 2008) comb. nov. is transferred from the genus *Leptoneta* Simon, 1872;the genus *Guineta* Lin & Li, 2010 syn. nov. is a junior synonym of Leptonetela; *Leptonetela gigachela*(Lin & Li, 2010) comb. nov. is transferred from *Guineta*. The genus Sinoneta Lin & Li, 2010 syn. nov. is a junior synonym of *Leptonetela*; *Leptonetela notabilis*(Lin & Li, 2010) comb. nov. and *Leptonetela sexdigiti*(Lin & Li, 2010) comb. nov. are transferred from *Sinoneta*; *Leptonetela sanchahe* Wang & Li nom. nov. is proposed as a replacement name for *Sinoneta palmata*(Chen et al, 2010) because *Leptonetela palmata* is preoccupied.

## INTRODUCTION

Subterranean ecosystems, such as caves and cracks, are evident mainly in karst areas, which represent nearly 4% of the rocky outcrops of the world. These environments are marked by permanent darkness, a lack of diurnal and annual rhythms, and extremely scarce food sources (Culver & White, 2005; Howarth, 1983; Poulson & White, 1969). Many studies show that despite stressful and unfavorable conditions, the subsurface habitat harbors diverse animal communities (mainly invertebrates) (Amara-Zettler et al, 2002; Flot et al, 2010; López-García et al, 2001; Mathieu et al, 1997; Niemiller et al, 2012; Sket, 1999). Troglobionts are expected to adopt strategies that are characterized by significant geographic isolation and numerous local endemics (Convey, 1997; Waterman, 2001). Because the diversity of possible adaptive responses declines with stress intensity (Nevo, 2001), evolution in harsh environments is also expected to be influenced by convergence (Little & Vrijenhoek, 2003; Rothschild & Mancinelli, 2001; Waterman, 2001). Therefore, in subterranean, and more generally in extreme environments, diversification and speciation processes should be largely influenced by island-like habitats, such as caves, allopatric speciation and vicariant events, and could be masked by morphological convergence. For these groups of organisms, morphology alone cannot determine species boundaries, so identifying morphologically inseparable cryptic or sibling species requires an integrative approach that often includes DNA analysis.

DNA barcoding relies on the use of a standardized DNA region as a tag for accurate and rapid species identification (Hebert & Gregory, 2005) and has been used to help overcome the 'taxonomic impediment' (Herbert et al, 2003a; Tautz et al, 2003). It aids in the identification of species in applied settings, the association of morphologically distinct life-cycle forms within a species, the detection of host-specific lineages and the detection of morphologically cryptic species (Miller & Foottit, 2009). DNA barcoding has been used in a diverse range of vertebrate and invertebrate taxa (Clare et al, 2007; Ratnasingham & Hebert, 2007) and has enabled an increasing number of taxa to be identified. For example, a survey of crustacean stygofauna suggests that there could be substantial levels of subterranean biodiversity hidden in Australia's acquifer (Asmyhr & Cooper, 2012). Nevertheless, the exclusive use of single-locus molecular gene fragments is not without risks, for identical mitochondrial DNA sequences can be present in unrelated species due to introgression, or incomplete lineage sorting (Ballard & Whitlock, 2004). Additionally, the use of a divergence threshold for distinguishing intra-vs. interspecific sequence variation (Hebert et al, 2003a) can seriously compromise species identification and suffers from severe statistical problems (Vences et al, 2005). Furthermore, species misidentification has been observed when a reference database is not comprehensive; such that is does not contain all the species of the group under study (Meyer & Paulay, 2005).

The South China karst, a United Nations Educational, Scientific and Cultural Organization (UNESCO) World Heritage Site since 2007, is noted for its karst features and landscapes as well as rich biodiversity. Numerous subterraean species have been reported in this region, especially invertebrate fauna (Zhang, 1986). The spider genus *Leptonetela* is discontinuously distributed in the South China karst and the Balkan Peninsula, a karstic region in Europe. The genus has 54 catalogued species (World Spider Catalog, 2017), and with one exception (*L. pungitia* Wang & Li, 2011), nearly all *Leptonetela* species are endemic to either a single cave or a cave system. The spiders are cave adapted as shown by morphological features, such as vestigial eyes and highly reduced skin pigmentation. Over the past nine years, we have conducted extensive surveys of subterranean biodiversity in Eurasia. More than 1 500 caves were visited, and we ultimately sampled 122 *Leptonetela* populations (caves). Rapid and accurate identification within this genus is difficult due to congeneric species sharing similar morphological traits, a lack of obvious morphological differences between closely related species and some species only differ in one or a few quantitative differences, such as the location, length ratio or thickness of the male pedipalpal tibial spines and the number of teeth on the median apophysis.

In this study, we test the usefulness of DNA barcoding for species identification in the subterranean genus *Leptonetela* and investigate the diversity of the genus. The standard molecular barcode, cytochrome c oxidase subunit Ⅰ (*COI*) was used. A species discovery method, automatic barcode gap discovery (ABGD) (Puillandre et al, 2012), and a species validation method, DNA barcoding gap analysis, (Hebert et al, 2003b) were both used, depending on whether the samples were partitioned prior to analysis. The main goals of our study were: (ⅰ) to test whether the *COI* barcoding fragment can reliably resolve and identify subterranean *Leptonetela* species by comparing the *COI* barcode fragment results with those from morphological data; (ⅱ) to test taxonomic value of morphological characters used in traditional methods of classification.

## MATERIALS AND METHODS

### Taxon sampling

**Figure 1:**
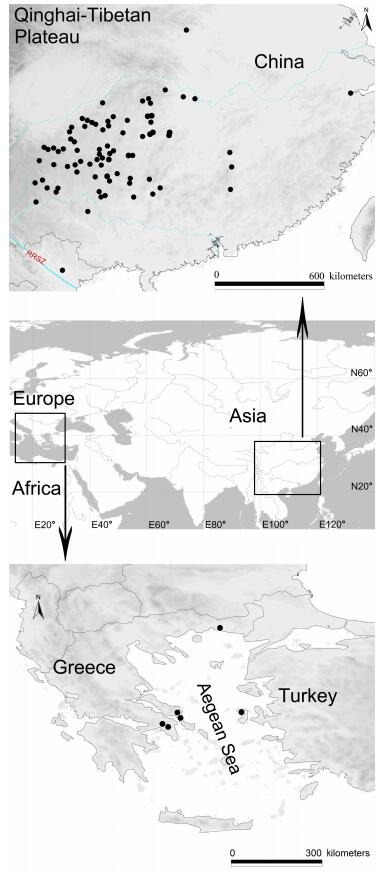
Area of endemic of *Leptonetela*

We sampled 624 *Leptonetela* individuals from 122 populations (caves) (Supplementary Table S1) in Eurasia (Insular and Peninsular Greece, and Southeast Asia; see inset in Figure 1). Nine individuals from three other genera of the family Leptonetidae were chosen as outgroups. All specimens were collected alive, fixed in absolute ethanol, and the legs were removed for subsequent DNA extraction. The remaining specimens were preserved in 80% ethanol for identification and morphological examination. Voucher specimens and all type specimens were deposited in the Institute of Zoology, Chinese Academy of Sciences (IZCAS), Beijing, China.

### Molecular protocols

Total genomic DNA was extracted using the Animal Genomic DNA Isolation Kit (Dingguo, Beijing, China) following the manufacturer's protocol. We amplified the cytochrome c oxidase subunit Ⅰ (*COI*) barcode region using the primer pairs LCOI490/HCO2198 (Folmer et al, 1994). PCR reaction conditions were: initial denaturation at 94 ℃ for 1 min; 35 cycles of denaturation at 94 ℃ for 1 min, annealing at 45 ℃ for 45 s, and elongation at 70 ℃ for 60 s; and a final extension at 72 ℃ for 5 min. The 25 μL PCR reactions included 17.25 μL of double-distilled H_2_O, 2.5 μL of 10× Taq buffer (mixed with MgCl_2_; TianGen Biotech, Beijing, China), 2.0 μL of dNTP Mix (2.5 mmol/L), 1 μL of each forward and reverse 10 μmol/L primer, 1 μL of DNA template, and 0.25 μL Taq DNA polymerase (2.5 U/μL; TianGen Biotech, Beijing, China). Double-stranded PCR products were visualized by agarose gel electrophoresis (1% agarose). PCR products were purified and sequenced by Sunny Biotechnology Co., Ltd (Shanghai, China) using the ABI 3730XL DNA analyser. Sequences were aligned using ClustalW in Mega 6.0 (Tamura et al, 2013), with visual inspection, translation, and manual adjustment to minimize alignment error. The most appropriate phylogenetic model for the sequence alignment was selected using jModelTest2 (Darriba et al, 2012) under the Akaike Information Criterion (Posada & Crandall, 1998).

### Phylogenetic analyses

Phylogenetic analyses were performed using maximum likelihood (ML) in RAXML v. 7.0.3 with the GTRCAT model (Stamatakis, 2006). One hundred replicate ML inferences were performed in the search for an optimal ML tree, each initiated with a random starting tree and employing the default rapid hill-climbing algorithm. Clade confidence was assessed with a rapid bootstrap of 1 000 replicates.

### Species delineation

We analyzed the *COI* barcode dataset (see Supplementary Table S1) using two species delineation methods. DNA barcoding gap analyses require an *a priori* species designation. Therefore, we divided the 624 *Leptonetela* individuals of 122 populations (caves) into 90 putative species based on morphological characters and geographic information. In our DNA barcoding gap analysis, we examined the overlap between the mean intraspecific and interspecific Kimura 2-parameter (K2P) (Kimura, 1980) and uncorrected *p*-distance (Nei & Kumar, 2000) for each candidate species, as calculated by Mega v. 6.0 (Tamura et al, 2013).

The automatic barcode gap discovery procedure (ABGD) (<xref ref-type="bibr" rid="b31-ZoolRes-38-6-321">Puillandre et al, 2012</xref>), which does not require assigning samples to putative species, calculates all pairwise distances in the dataset, evaluates intraspecific divergences, and then sorts the samples into candidate species using the calculated distances. We performed ABGD analyses online (<a href="http://wwwabi.snv.jussieu.fr/public/abgd/" target="_blank">http://wwwabi.snv.jussieu.fr/public/abgd/</a>), using three different distance metrics: Jukes-Cantor (JC69) (<xref ref-type="bibr" rid="b18-ZoolRes-38-6-321">Jukes & Cantor, 1969</xref>), Kimura 2-parameter (K2P) (<xref ref-type="bibr" rid="b19-ZoolRes-38-6-321">Kimura, 1980</xref>), and simple distance (<italic>p</italic>-distance) (<xref ref-type="bibr" rid="b26-ZoolRes-38-6-321">Nei & Kumar, 2000</xref>). We analyzed the data using two different values for the parameters Pmin (0.0001 and 0.001), Pmax (0.1 and 0.2), and relative gap width (X=1 or 1.5), with all other parameters at default values. 

### Taxonomy

The terminology and the measurements in this paper generally follow Wang & Li (2011) and Ledford et al (2011). All measurements were taken in millimetres (mm). The left palpi of male spiders are illustrated, except where otherwise indicated. Abbreviations used in text include: PL: prolateral lobe; E: embolus; C: conductor; MA: median apophysis; At: atrium; SS: spermathecae stalk; SH: spermathecae.

### Nomenclatural acts

This article conforms to the requirements of the amended International Code of Zoological Nomenclature. All nomenclatural acts contained within this published work have been registered in ZooBank. The ZooBank LSIDs (Life Science Identifiers) can be resolved and the associated information viewed by appending the LSID to the prefix "<a href="http://zoobank.org/" target="_blank">http://zoobank.org/</a>". The LSID for this publication is: urn:lsid:zoobank.org:pub:7ECB1BDC-8893-4D0F-8BEA-17ECE327FC47 

## RESULTS

In total, 624 DNA barcodes were analyzed. A full list of the analyzed specimens can be found in Supplementary Table S1. Fragment lengths of the analyzed DNA barcodes ranged from 107 (0.005%) to 617 bp (89%). For all populations, except *L. kanellisi* and *L. robustispina*, four or more DNA barcodes were generated. All nucleotides were translated into functional protein sequences in the correct reading frame, with no stop codons or indels observed. Similar to other arthropod studies, our data indicated a high AT-content for this mitochondrial gene fragment: the mean sequence compositions were A=20.5%, C=12.6%, G=24.4%, T=41.4%.

### Phylogenetic inference

**Figure 2:**
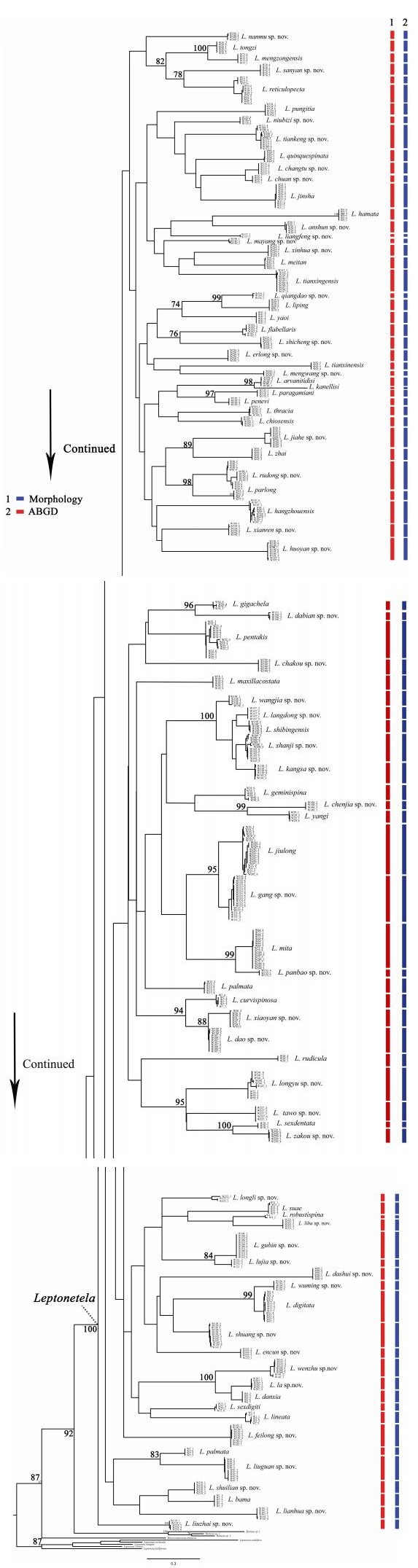
Maximum likelihood *COI* gene tree for 624 terminals of *Leptonetela*, with the results of two different species delimitation approaches

The ML gene tree topology suggests that *Leptonetela* is monophyletic, with the node highly supported (Figure 2; bootstrap value, BS=92). Our analyses revealed all *Leptonetela* species formed non-overlapping clusters, with bootstrap support values of 100. In contrast, relationships among putative species were largely unresolved, usually with low bootstrap support on the ML gene tree, particularly at deeper phylogenetic levels.

### Species delineation

**Figure 3:**
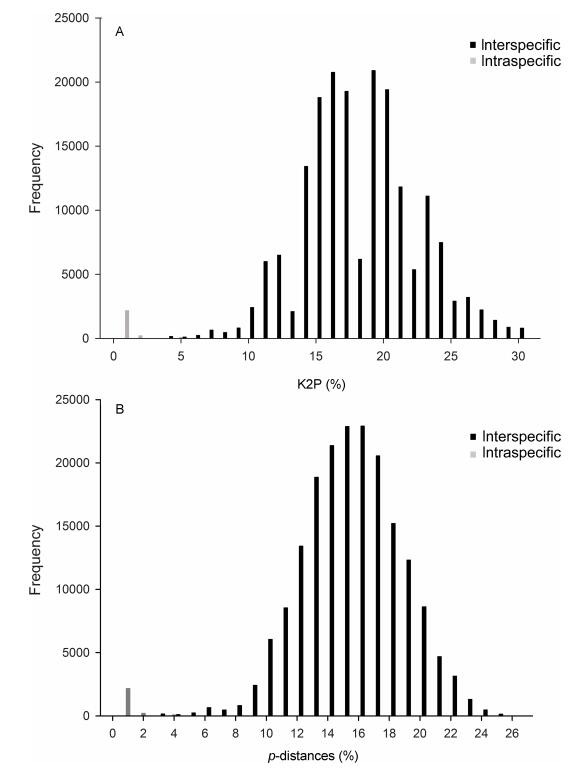
DNA barcoding for *Leptonetela*

DNA barcoding gap analysis: Based on our *a priori* species hypotheses, Interaspecific divergences ranged from zero to 5.3/5.0% (K2P/uncorrected *p*-distance) whereas interspecific distances were between 3.1/3% and 31.9/25% (K2P/uncorrected *p*-distance). Maximum intraspecific distances > 3% were found for two species, including *L. reticulopecta* (4.3/4.0%), and *L. pentakis* Lin & Li, 2010 (5.3/5%). The lowest interspecific distance were revealed for the two species pairs *L.changtu* Wang & Li **sp. nov.** with *L. chuan* Wang & Li **sp. nov.** and *L. kangsa* Wang & Li **sp. nov.** with *L. shibingensis* Guo, Yu & Chen, 2016 with a value of 3.1/3%. Minimum interspecific pairwise distances < 5%, and > 3% were found for two species pairs: *L. shibingensis* with *L. shanji* Wang & Li **sp. nov.** and *L. dao* Wang & Li **sp. nov.** with *L. xiaoyan* Wang & Li **sp. nov.** The mean interspecific distance between the 90 tentative species was 17.9/15.6% (K2P/uncorrected *p*-distance), and the mean intraspecific distance within each species was 0.2% (both K2P and uncorrected *p*-distance) in *Leptonetela*. A histogram of the gap and overlap between intra-and interspecies genetic distances are show in Figure 3.

### ABGD analysis

The ABGD analyses of the *COI* dataset, using the originally specified parameter combinations and partitions resulted mostly in 90 distinct species that correspond to the 90 species observed in the previous taxonomic hypotheses based on morphology. The result was the same regardless of the model of evolution employed (Jukes-Cantor (JC), K2P, Simple Distance). The settings *P* min/*P* max=0.0001/0.2 yielded the most significant *P* values. However, at lower values of prior intraspecific distance (*P*), recursive partitioning of ABGD recognized more species (Table 1) when *P* min/*P* max=0.0001/0.2, *P* values=0.159, JC and K2P distance resulted in 98 species, and simple distance resulted in 95 species.

**Table 1:** Results of the automatic barcode gap discovery (ABGD) analyses

		Prior intraspecific divergence (*P*)									
Substitution mode	*P* min/*P* max	X	Partition	0.001	0.0017	0.0028	0.0046	0.0077	0.0129	0.0215	0.0359
JC	0.001/0.1	1.5	Initial	90	90	90	90	90	90	90	90
			Recursive	171	136	136	106	106	99	92	90
K2P	0.001/0.1	1.5	Initial	90	90	90	90	90	90	90	90
			Recursive	171	136	136	106	106	99	92	90
Simple	0.001/0.1	1.5	Initial	90	90	90	90	90	90	90	90
			Recursive	105	105	105	97	96	95	92	90
JC	0.001/0.1	1	Initial	90	90	90	90	90	90	90	90
			Recursive	169	132	132	106	106	99	92	90
K2P	0.001/0.1	1	Initial	90	90	90	90	90	90	90	90
			Recursive	172	137	137	106	106	99	92	90
Simple	0.001/0.1	1	Initial	90	90	90	90	90	90	90	90
			Recursive	105	105	105	97	96	95	92	90
				0.0001	0.0002	0.0005	0.0013	0.0029	0.0068	0.0159	0.0369
JC	0.0001/0.2	1.5	Initial	90	90	90	90	90	90	90	90
			Recursive	171	171	171	171	136	106	98	90
K2P	0.0001/0.2	1.5	Initial	90	90	90	90	90	90	90	90
			Recursive	171	171	171	171	136	106	98	90
Simple	0.0001/0.2	1.5	Initial	90	90	90	90	90	90	90	90
			Recursive	105	105	105	105	105	96	95	90

## DISCUSSION

DNA barcoding is widely recognized as a useful tool for species identification across the animal kingdom (Chesters et al, 2012; Wang et al, 2011). Our research represents an important step towards the application of DNA barcodes for identification of *Leptonetela* taxa, and for 119 taxa (97%), our data represent the first published DNA barcodes.

Classically, geographic isolation is considered a primary feature of troglobitic taxa (Hedin, 1997; Hedin & Thomas, 2010). Our DNA barcoding result is consistent with this view and similar to other DNA barcoding studies, in which *COI* showed high genetic structure between populations within species (Tavares et al, 2001).

Choosing appropriate thresholds that can delimit species is one of the main challenges and concerns for DNA barcoding research (Ferguson, 2002). Our DNA barcoding gap analysis shows an overlap in the range of intra-and interspecific *COI* sequence divergence. The interspecific genetic divergences between *L. chuan* Wang & Li **sp. nov.** and *L. changtu* Wang & Li **sp. nov.**, *L. kangsa* Wang & Li **sp. nov.** and *L. shibingensis*, as well as between *L. shibingensis* and *L. shanji* Wang & Li **sp. nov.** was 3.1/3.0% based on K2P/uncorrected and *p*-distance models. Compared with other species *L. chuan* Wang & Li **sp. nov.**, and *L. changtu* Wang & Li **sp. nov.**, *L. kangsa* Wang & Li **sp. nov.**, *L. shibingensis* and *L. shanji* Wang & Li **sp. nov.** are more closely distributed. In morphology, *L. chuan* Wang & Li **sp. nov.** and *L. changtu* Wang & Li **sp. nov.** can be distinguished by the shape of the median apophysis and the conductor (median apophysis palm-shaped, edge with sclerotized spots, conductor semicircular in *L. changtu* Wang & Li **sp. nov.**, median apophysis rectangular, with 5 larger teeth distally, conductor triangular in ventral view in *L. chuan* Wang & Li **sp. nov.**); *L. kangsa* Wang & Li **sp. nov.**, *L. shibingensis* and *L. shanji* Wang & Li **sp. nov.** can be distinguished by the location and pattern of male pedipalpal tibial spines (Ⅰ spine located at the middle in *L. shibingensis* and *L. shanji* Wang & Li **sp. nov.**; Ⅰ spine asymmetrically bifurcated in *L. shanji* Wang & Li **sp. nov.**, male pedipalpal tibia Ⅰ spine located at base and not bifurcated in *L. kangsa* Wang & Li **sp. nov.**). Nevertheless, we found two species with maximum pairwise distance > 3%, including *L. reticulopecta* (specimens from Tianshegnqiao Cave are clearly distant from the rest) with 4.3/4.0%, *L. pentakis* (specimens from Liaoya cave is clearly distant from the rest) with 5.3/5.0%. Then we achieved a threshold of 3.11/3.0% (K2P/uncorrected and *p*-distance), excluding taxa from Tianshegnqiao Cave and Liaoya Cave. This threshold was interestingly close to the 3% commonly used in barcoding literature (Hebert et al, 2003a, b).

Here, we were highly successful using ABGD for identification. In ABGD analysis, the taxa from Tianshegnqiao Cave and Liaoya Cave were identified as *L. reticulopecta* and *L. pentakis*, respectivelly. Given that all specimens of *L. reticulopecta* and *L. pentakis* are morphologically very similar, we are currently unable to ascertain if the observed genetic distances simply represent a high level of intraspecific variation or reflect cryptic species between the taxa of *L. reticulopecta* and *L. pentakis*. To answer this question, more specimens need to be collected and analyzed, using both morphological characters and nuclear sequence data.

In conclusion, our study demonstrates the power of an integrative approach, in which both classical and DNA barcoding taxonomy complements each other and both contribute to a more accurate taxonomic classification.

### Taxonomy

Key to species of *Leptonetela*

(Mostly referring to characters of the male pedipalp)

1  Spermathecae thin and loosely twisted…………………………*L. strinatii* (Brignoli, 1976) (male unknown)

-  Not as above……………………………………………………………2

2  Male pedipalp with median apophysis………………………3

-  Male pedipalp without median apophysis…………………9

3  Median apophysis like pine needles, sclerotized………4

-  Not as above……………………………………………………………33

4  Median apophysis appears as 4 pine needle-like appendages…………………………………………………………………………5

-  Median apophysis divided into more or less than 4 pine needle-shaped appendages…………………………………………6

5  Tibia Ⅰ spine strong, conspicuous, with bifurcated tip……………………………………………………………………*L. chakou* **sp.nov.**

-  Tibia Ⅰ spine strong, located at the middle of tibia prolaterally……………………………………………*L. grandispina* Lin & Li, 2010

6  Cymbium roughly double the length of bulb………………7

-  Cymbium roughly the same length as bulb…………………8

7  Median apophysis divided into 15 pine needle-like appendages………………………*L. liuzhai* Wang & Li **sp. nov.**

-  Median apophysis divided into 2 pine needle-like appendages………………………*L. shuilian* Wang & Li **sp. nov.**

8  Cymbium constricted medially, median apophysis divided into 5 pine needle-like appendages…………………………………………………………………………………*L. pentakis* Lin & Li, 2010

-  Cymbium not constricted medially, median apophysis divided into 2 pine needle-like appendages………………………………………………………………………………*L. dao* Wang & Li **sp. nov.**

9  Male pedipalp with 5 tibial spines prolaterally…………10

-  Male pedipalp with more than 5 tibial spines prolaterally…29

10  Cymbium constricted and wrinkled medially……………11

-  Cymbium not constricted or wrinkled medially…………22

11  Tibial spines slender and without bifurcated tip…………12

-  Tibial spines strong or with bifurcated tip…………………16

12  Prolateral lobe tongue-shaped…………………………………13

-  Prolateral lobe absent………*L. sanyan* Wang & Li **sp. nov.**

13  Pedipalpal tibia with one spine significantly longer than other spines………………………………………………………………………14

-  Pedipalp tibia Ⅰ, Ⅱ spines nearly the same length…………………………………………………………………*L. meitan* Lin & Li, 2010

14  Conductor bamboo leaf-shaped in ventral view…………15

-  Conductor C-shaped in ventral view………………………………………………………………………*L. liangfeng* Wang & Li **sp. nov.**

15  Embolus and conductor long, intersecting……………………………………………………………………………*L. suae* Lin & Li, 2010

-  Embolus and conductor short, not intersecting………………………………………………………………………*L. tongzi* Lin & Li, 2010

16  Pedipalpal tibia Ⅰ spine with bifurcated tip…………………17

-  Pedipalpal tibia Ⅰ spine without bifurcated tip……………19

17  Pedipalpal tibia Ⅰ spine strong, asymmetrically bifurcate…18

-  Pedipalpal tibia Ⅰ spine slender, symmetrically bifurcate………………………………………………………*L. danxia* Lin & Li, 2010

18  Pedipalpal tibia Ⅰ spine located proximally at tibia, thin spines Ⅱ, Ⅴ and Ⅵ arranged in a triangle, conductor bamboo leaf-shaped in ventral view………*L. andreevi* Deltshev, 1985

-  Pedipalpal tibia Ⅰ spine located at distal 1/3 of tibia, conductor C shaped in ventral view………………………………………………………………………………*L. furcaspina* Lin & Li, 2010

19  Pedipalpal tibia Ⅰ spine longest…………………………………20

-  Pedipalpal tibia Ⅱ spine longest………………………………21

20  Pedipalpal tibia Ⅰ spine bent distally, conductor reduced……………………………………………*L. langdong* Wang & Li **sp. nov.**

-  Pedipalpal tibia Ⅰ spine not bent distally, conductor semicircular in ventral view……………………………*L. yaoi* Wang & Li, 2011

21  Eyes absent, pedipalpal tibia Ⅲ, Ⅴ and Ⅵ spines more slender than Ⅰ, Ⅱ spines……………*L. lineata* Wang & Li, 2011

-  Six eyes, pedipalpal tibial spines equally strong………………………………………………………………*L. caucasica* Dunin, 1990

22  Conductor developed………………………………………………23

-  Conductor reduced…………………………………………………26

23  Pedipalpal tibia Ⅰ spine without bifurcated tip……………24

-  Pedipalpal tibia Ⅰ spine with bifurcated tip, other spines concentrated distally, tip of conductor bifurcated…………………………………………………………………*L. anshun* Lin & Li, 2010

24  Conductor bamboo leaf-shaped in ventral view…………25

-  Conductor C-shaped in ventral view, pedipalpal tibia Ⅰ spine longest………………………………*L. dashui* Wang & Li **sp. nov.**

25  Pedipalpal tibia Ⅰ spine strong, prolateral bulbal lobe reduced………………………………………*L. qiangdao* Wang & Li **sp. nov.**

-  Pedipalpal tibia Ⅰ spine slender, prolateral bulbal lobe tongue-shaped……………*L. nuda* (Chen, Jia & Wang, 2010)

26  Cymbium with a distal and proximal spine prolaterally, pedipalpal tibial spines equidistant……………………………………………………………………………*L. curvispinosa* Lin & Li, 2010

-  Not as above……………………………………………………………27

27  Pedipalpal tibia Ⅰ spine slender, asymmetrically bifurcated…………………………………………*L. wangjia* Wang & Li **sp. nov.**

-  Not as above……………………………………………………………28

28  Pedipalpal tibia Ⅰ, Ⅱ, and Ⅲ spines concentrated in the mid of tibia, 2 additional spines located distally, prolateral bulbal lobe reduced……………………*L. maxillacostata* Lin & Li, 2010

-  Pedipalpal tibia Ⅰ spine longest, located far from others, prolateral lobe small, tongue shaped…………………………………………………………………………*L. chenjia* Wang & Li **sp. nov.**

29  Male pedipalp with 6 tibial spines retrolaterally…………30

-  Male pedipalp with 7 tibial spines retrolaterally…………32

30  Pedipalpal tibia Ⅰ, Ⅱ spines strong, equally length, Ⅱ spine asymmetrically bifurcated, conductor reduced………………………………………………………………*L. gang* Wang & Li **sp. nov.**

-  Pedipalpal tibial spines slender, not bifurcated, conductor developed………………………………………………………………31

31  Pedipalpal tibia with 2 large spines prolaterally, cymbium not constricted medially, earlobe-shaped process absent, and cymbium long, twice the length of bulb…………………………………………………………………*L. gigachela* (Lin & Li, 2010)

-  Pedipalpal tibia without prolateral spines, cymbium constricted medially, retrolaterally attaching an earlobe-shaped process, cymbium less than twice the length of bulb…………………………………………………………………*L. wenzhu* Wang & Li **sp. nov.**

32  Cymbium with 1 horn-shaped spine on the earlobe-shaped process, conductor thin, triangular in ventral view………………………………………………………*L. rudong* Wang & Li **sp. nov.**

-  Earlobe-shaped process of cymbium without spine, conductor broad, C shaped in ventral view…*L. la* Wang & Li **sp. nov.**

33  Median apophysis like a pointed process or lamelliform..34

-  Median apophysis finger-shaped or harrow-like………50

34  Cymbium not constricted medially, earlobe-shaped process reduced……………………………………………………………………35

-  Cymbium constricted medially, earlobe-shaped process developed…………………………………………………………………38

35  Male pedipalpal tibia with 6 spines retrolaterally……………36

-  Male pedipalpal tibia only with 5 spines retrolaterally……37

36  Pedipalpal tibia with 4 long spines prolaterally, the retrolateral Ⅰ spine longest, Ⅱ Ⅲ spines short and strong, median apophysis pointed, conductor bamboo leaf-shaped………………………………………………………………………………*L. bama* Lin & Li, 2010

-  Pedipalpal tibia with 3 long spines prolaterally, the retrolateral Ⅰ spine longest and strongest, median apophysis "M"-shaped, conductor reduced………………………*L. yangi* Lin & Li, 2010

37  Pedipalpal tibia with 1 long spine prolaterally, the retrolateral Ⅰ spine longest and strongest, median apophysis pointed, conductor bamboo leaf-shaped………*L. liping* Lin & Li, 2010

-  Pedipalpal tibia with 3 long spines prolaterally, the retrolateral spines Ⅰ slender, and longest, median apophysis obtuse triangle shaped, conductor narrow, triangular……………………………………………………………*L. mayang* Wang & Li **sp. nov.**

38  Cymbium with 1 strong spine on the earlobe-shaped process…………………………………………………………………………………39

-  No spine on the earlobe-shaped process…………………43

39  Male pedipalp tibia with 5 spines retrolaterally…………40

-  Male pedipalp tibia with more than 5 spines retrolaterally…41

40  Cymbium with 1 curved spine retrolaterally, median apophysis pointed, with 3 sclerotized apices distally, conductor C shaped…………………………………*L. jiahe* Wang & Li **sp. nov.**

-  Cymbium without curved spine retrolaterally, median apophysis punctate in ventral view, conductor vestigial………………………………………………*L. panbao* Wang & Li **sp. nov.**

41  Pedipalpal tibia Ⅰ spine strong, Ⅱ spine asymmetrically bifurcated, median apophysis lamelliform, conductor triangular…………………………………………………*L. jiulong* Lin & Li, 2010

-  Pedipalpal tibia Ⅰ spine slender, not bifurcated…………42

42  Pedipalpal tibia with 3 long spines prolaterally, 6 spines retrolaterally, median apophysis semicircular………………………………………………………………*L. parlonga* Wang & Li, 2011

-  Pedipalpal tibia with 5 long spines prolaterally, 7 spines retrolaterally, median apophysis mita-shaped, embolus with 1 tooth distally……………………………*L. mita* Wang & Li, 2011

43  Pedipalpal tibia with 5 spines retrolaterally………………44

-  Pedipalpal tibia with more than 5 spines retrolaterally……49

44  Pedipalpal tibia with 3 long spines prolaterally………………45

-  Pedipalpal tibia with 1 or 2 long spines prolaterally………47

45  Conductor C shaped in ventral view……………………………46

-  Conductor bamboo leaf-shaped in ventral view, retrolateral spines Ⅰ longest, median apophysis triangular………………………………………………………………*L. xianren* Wang & Li **sp. nov.**

46  Pedipalpal tibia Ⅰ spine longest, the rest concentrated at distal end of tibia, median apophysis spatula-shaped in ventral view…………………………*L. rudicula* Wang & Li, 2011

-  Pedipalpal tibia Ⅰ spine longest, Ⅰ, Ⅱ, and Ⅲ spines equally strong, median apophysis single quote shaped, " ′ " in ventral view…………………………*L. longli* Wang & Li **sp. nov.**

47  Pedipalpal tibia with 1 long spine prolaterally, median apophysis tongue-shaped, conductor triangular……………………………………………………………*L. pungitia* Wang & Li, 2011

-  Pedipalpal tibia with 2 long spines prolaterally………………48

48  Pedipalpal tibia Ⅰ spine strongest, Ⅲ-Ⅴ spines in a triangular arrangement, median apophysis punctate, conductor triangular…………………………*L. chiosensis* Wang & Li, 2011

-  Pedipalpal tibia Ⅰ spine longest, spine Ⅰ-Ⅲ equally strong, Ⅳ-Ⅴ situated distally median apophysis "m"-shaped, conductor triangular…………*L. feilong* Wang & Li **sp. nov.**

49  Pedipalpal tibia with 6 spines retrolaterally, tibia Ⅰ spine close to others, median apophysis flake-like, sclerotized distally, conductor broad, undulate distally………………………………………………………………*L. tiankeng* Wang & Li **sp. nov.**

-  Pedipalpal tibia with 7 spines retrolaterally, tibia Ⅰ spine distant from others, median apophysis small worm-shaped, conductor thin, triangular…………………………………………………………………*L. lophacantha* (Chen, Jia & Wang, 2010)

50  Median apophysis index finger like…………………………51

-  Median apophysis harrow-like…………………………………72

51  Embolus bifurcated……………*L. xinhua* Wang & Li **sp. nov.**

-  Embolus not bifurcated……………………………………………52

52  Base of median apophysis swollen…………………………53

-  Base of median apophysis not swollen……………………56

53  Male pedipalpal tibia with 5 spines retrolaterally………54

-  Male pedipalpal tibia with 6 slender spines retrolaterally, spines Ⅰ longest, conductor smooth, semicircular……………………………………………*L. quinquespinata* (Chen & Zhu, 2008)

54  Pedipalpal tibia Ⅰ spine much stronger than Ⅱ, asymmetrically bifurcated……………………………………*L. jinsha* Lin & Li, 2010

-  Pedipalpal tibia Ⅰ spine similarly strong as Ⅱ, not bifurcated…………………………………………………………………………………55

55. Cymbium constricted medially, earlobe-shaped process with 2 long, curved spines retrolaterally, base of median apophysis distinctly swollen, conductor smooth, broad, semicircular…………………………*L. gubin* Wang & Li **sp. nov.**

-  Cymbium not constricted medially, earlobe-shaped process small, base of median apophysis slightly swollen, conductor rugose, thin, triangular……………*L. lujia* Wang & Li **sp. nov.**

56  Median apophysis bifurcated distally…………………………………………………………………………*L. wuming* Wang & Li **sp. nov.**

-  Median apophysis not bifurcated distally…………………57

57  Pedipalpal tibia Ⅰ spine located at the base of tibia……58

-  Pedipalpal tibia Ⅰ spine located medially……………………59

58  Pedipalpal tibia Ⅰ spine asymmetrically bifurcated, tibia with 4 long spines prolaterally………………………………………………………………………………*L. shibingensis* Guo, Yu & Chen, 2016

-  Pedipalpal tibia Ⅰ spine not bifurcated…………………………………………………………………………*L. kangsa* Wang & Li **sp. nov.**

59  Male pedipalp tibia with 6 spines retrolaterally…………60

-  Male pedipalp tibia with 5 spines retrolaterally…………62

60  Pedipalpal tibia with 4 spines prolaterally, cymbium with 1 curved spine at the base of retrolateral surface, median apophysis weakly sclerotized…………………………………………………………………………………*L. xiaoyan* Wang & Li **sp. nov.**

-  Not as above……………………………………………………………61

61  Male pedipalp tibia with 2 spines prolaterally, conductor short, broad and rugose………*L. oktocantha* Lin & Li, 2010

-  Male Pedipalp tibia without spine prolaterally, conductor smooth, semicircular……………*L. hexacantha* Lin & Li, 2010

62  Median apophysis curved distally……………………………63

-  Median apophysis not curved distally………………………65

63  Cymbium with 1 horn-shaped spine on the earlobe-shaped process retrolaterally, tibia spines gradually shorted, conductor smooth, C shaped……………………………………………………………………*L. mengzongensis* Wang & Li, 2011

-  Cymbium without spine on the earlobe-shaped process retrolaterally………………………………………………………………64

64  Male pedipalp tibia with 2 long setae prolaterally, tibia Ⅰ Ⅱ and Ⅲ spines equally in length, conductor broad, semicircular…………………………………………………*L. hamata* Lin & Li, 2010

-  Male pedipalp tibia with 4 long spines prolaterally, tibia Ⅰ Ⅱ spines equally in length, conductor long, curved distally……………………………………………*L. tetracantha* Lin & Li, 2010

65  Pedipalpal tibia Ⅰ spur strong……………………………………66

-  Pedipalpal tibia Ⅰ spine slender…………………………………70

66  Pedipalpal tibia Ⅰ spine asymmetrically bifurcated………67

-  Pedipalpal tibia Ⅰ spine not bifurcated, conductor broad, C shaped, median apophysis distinctly sclerotized…………………………………………………………*L. reticulopecta* Lin & Li, 2010

67  Median apophysis tapering……………………………………68

-  Median apophysis blunt…………………………………………69

68  Pedipalpal tibia Ⅰ spine located at the middle of tibia………………………………………………………*L. shanji* Wang & Li **sp. nov.**

-  Pedipalpal tibia Ⅰ spine located at the basal of tibia………………………………………………………………*L. digitata* Lin & Li, 2010

69  Pedipalpal tibia Ⅱ-Ⅴ spines slender flexible, Ⅰ and Ⅱ spines equally length, conductor shorter than median apophysis………………………………………*L. tianxinensis* (Tong & Li, 2008)

-  Pedipalpal tibia Ⅱ spine slender, Ⅲ spine strong, conductor longer than median apophysis……………………………………………………………………………………*L. nanmu* Wang & Li **sp. nov.**

70  Pedipalpal tibia Ⅰ spine located at the base of tibia, other spines concentrated distally on tibia, conductor smooth, semicircular………………………*L. huoyan* Wang & Li **sp. nov.**

-  Not as above……………………………………………………………71

71  Pedipalpal tibia Ⅰ Ⅱ spines adjacent, the rest short, concentrated distally, outermost plumose, tibia with 2 spines prolaterally, conductor bifurcate………………………………………………………………………*L. geminispina* Lin & Li, 2010

-  Pedipalpal tibia Ⅰ-Ⅳ spines spaced at regular intervals, Ⅳ and Ⅴ adjacent, tibia Ⅰ-Ⅲ equal in length, conductor short, C shaped…………………………*L. tianxingensis* Wang & Li, 2011

72  Median apophysis harrow-like, horrow pin reduced to sclerotized spots………………………………………………………73

-  Median apophysis harrow-like, horrow pin not reduced…75

73  Pedipalpal tibial spines slender, equally strong, median apophysis long, half the length of bulb………………………………………………………………………*L. liuguan* Wang & Li **sp. nov.**

-  Pedipalpal tibial spines not equally strong, median apophysis short, 1/5 the length of bulb…………………………74

74  Pedipalpal tibia Ⅰ, Ⅱ spines equally strong, stronger than other spines, Ⅲ-Ⅴ in triangular arrangement, cymbium constricted medially, with one curved spine at the base of constriction retrolaterally…………*L. penevi* Wang & Li, 2016

-  Pedipalpal tibia Ⅰ Ⅱ Ⅲ spines equally strong, stronger than other spines, Ⅲ-Ⅴ not triangular arrangement, cymbium not constricted medially…………*L. changtu* Wang & Li **sp. nov.**

75  Median apophysisi harrow-like, the horrow pin not constant in size………………………………………………………………………76

-  Median apophysisi harrow-like, the horrow pin constant in size…………………………………………………………………………80

76  Pedipalpal tibia Ⅰ spine not bifurcated…………………………77

-  Pedipalpal tibia Ⅰ spine strong, asymmetrically bifurcated, other 4 spines slender, median apophysis with 5 small teeth and 1 large, horn-shaped tooth…………………………………………………………………………*L. lianhua* Wang & Li **sp. nov.**

77  Pedipalpal tibia Ⅰ spine longest…………………………………78

-  Pedipalpal tibia Ⅱ spine longest………………………………79

78  Median apophysis palmate, with six teeth distally…………………………………………*L. megaloda* (Chen, Jia & Wang, 2010)

-  Median apophysis antler-like, with 4 small teeth and 1 large tooth, which bears 2 small teeth…………………………………………………………………………………*L. niubizi* Wang & Li **sp. nov.**

79  Two large teeth on the periphery of median apophysis, 2 small teeth in the middle……………………………………………………………………*L. hangzhouensis* (Chen, Shen & Gao, 1984)

-  Two large teeth on the periphery of median apophysis, 5 small teeth in the middle.. *L. microdonta* (Xu & Song, 1983)

80  Median apophysis short and broad…………………………81

-  Median apophysis long and thin……………………………87

81  Pedipalpal tibia Ⅰ spine strongest……………………………82

-  Pedipalpal tibia Ⅰ spine not strongest………………………83

82  Pedipalpal tibia Ⅰ, Ⅱ spines equally strong, stronger than other 3 spines, median apophysis with 6 small teeth apically………………………………*L. identica* (Chen, Jia & Wang, 2010)

-  Pedipalpal tibia Ⅱ, Ⅲ spines equally strong, spine Ⅱ longest, median apophysis with 5 sharp teeth apically……………………………………………………………………………*L. meiwang* sp.nov.

83  Pedipalpal tibia Ⅰ spine bifurcated……………………………84

-  Pedipalpal tibia Ⅰ spine not bifurcated………………………89

84  Distal edge of median apophysis with 6 teeth……………85

-  Distal edge of median apophysis with 5 or 10 teeth…86

85  Teeth of median apophysis needle-shaped, earlobe-shaped process of cymbium absent; in the female, anterior margin of atrium with one pointed process medially…………………………………………………………………*L. zakou* Wang & Li **sp. nov.**

-  Teeth of median apophysis normal, cymbium with earlobe-shaped process, female anterior margin of atrium without pointed process………………*L. sexdentata* Wang & Li, 2011

86  Distal edge of median apophysis with 5 teeth, conductor C shaped, tip of conductor undulate in the female, anterior margin of atrium with one pointed process medially…………………………………*L. longyu* Wang & Li **sp. nov.**

-  Distal edge of median apophysis with 10 teeth, conductor C shaped, distal edge of conductor smooth; in the female, anterior margin of atrium without pointed process…………………………………………………*L. shicheng* Wang & Li **sp. nov.**

87  Pedipalpal tibia Ⅱ spine tapering………………………………88

-  Pedipalpal tibia Ⅰ spine blunt…*L. flabellaris* Wang & Li, 2011

88  Distal edge of median apophysis with 5 teeth, conductor short, C shaped………………………*L. palmata* Lin & Li, 2010

-  Distal edge of median apophysis with 7 teeth, conductor long, triangular shaped………………………………………………………………………………*L. kanellisi* (Deeleman-Reinhold, 1971)

89  Pedipalpal tibia with clusters of short spines dorsally……90

-  Pedipalpal tibia without clusters of short spines dorsally………………………………………………………………………………………91

90  Distal edge of median apophysis linear, with 8 teeth……………………………………………………*L. encun* Wang & Li **sp. nov.**

-  Distal edge of median apophysis semicircular, with 12 teeth………………………*L. robustispina* (Chen, Jia & Wang, 2010)

91  Base of pedipalpal tibia swollen………………………………92

-  Base of pedipalpal tibia not swollen…………………………96

92  Pedipalpal tibia Ⅰ spine bifurcate………………………………93

-  Pedipalpal tibia Ⅰ spine trifurcate…………………………………………………………………………………*L. notabilis* (Lin & Li, 2010)

93  Conductor triangular, longer than median apophysis, median apophysis with 7 teeth………………………………………………94

-  Conductor C shaped, shorter than median apophysis, median apophysis with 6 teeth……………………………………95

94  Spermathecae not twisted distally………………………………………………………………………………*L. shuang* Wang & Li **sp. nov.**

-  Spermathecae twisted distally……………………………………………………………………………*L. sanchahe* Wang & Li **nom. nov.**

95  Spermathecae weakly twisted……………………………………………………………………………*L. sexdigiti* (Lin & Li, 2011)

-  Spermathecae strongly twisted………………………………………………………………………………………*L. lihu* Wang & Li **sp. nov.**

96  Pedipalpal tibia Ⅰ spine strongset……………………………97

-  Pedipalpal tibia Ⅰ, Ⅱ, Ⅲ spines equally strong, stronger than other spines……………………………………………………………103

97  Pedipalpal tibia Ⅱ spine bifurcate………………………………98

-  Pedipalpal tibia Ⅰ spine not bifurcate………………………101

98  Pedipalpal tibia Ⅱ-Ⅴ spine slender, curved, equally strong…………………………………………………………………………………99

-  Pedipalpal tibia Ⅱ-Ⅴ spine not equally strong…………100

99  Distal edge of median apophysis with 6 teeth……………………………………………………*L. arvanitidisi* Wang & Li, 2016

-  Distal edge of median apophysis with 5 teeth………………………………………………………*L. erlong* Wang & Li **sp. nov.**

100  Distal edge of median apophysis with 4 teeth, tibia Ⅱ, Ⅲ spines equally strong, stronger than other 2 spines………………………………………………*L. tawo* Wang & Li **sp. nov.**

-  Distal edge of median apophysis with 3 teeth, tibia Ⅲ-Ⅴ spines equally strong, slender than spine Ⅱ……………………………………………………………*L. paragamiani* Wang & Li, 2016

101  Pedipalpal tibia Ⅱ-Ⅴ spines equally strong………………102

-  Pedipalpal tibia Ⅲ-Ⅴ spines equally strong, slender than spine Ⅱ, distal edge of median apophysis with 4 teeth……………………………………………………*L. deltshevi* (Brignoli, 1979)

102  Distal edge of median apophysis with 5 teeth, conductor C shaped…………………………*L. gittenbergeri* Wang & Li, 2011

-  Distal edge of median apophysis with 6 teeth, conductor semicircular………………………………*L. zhai* Wang & Li, 2011

103  Pedipalpal tibia Ⅰ Ⅱ spines equally strong……………………………………………………………………………*L. thracia* Gasparo, 2005

-  Pedipalpal tibia Ⅱ and Ⅲ spines equally strong………104

104  Distal edge of median apophysis with 3 teeth, tibia with 3 large spines prolaterally………*L. dabian* Wang & Li **sp. nov.**

-  Distal edge of median apophysis with 6 teeth, tibia with 6 long setae prolaterally…………*L. chuan* Wang & Li **sp. nov.**

### Family Leptonetidae Simon, 1890

### Genus *Leptonetela* Kratochvíl, 1978

**Type species:** *Leptonetela kanellisi* (Deeleman-Reinhold, 1971) from Greece.

**Diagnosis.** The genus *Leptonetela* can be distinguished from other leptonetid genera by the following combination of male pedipalpal characters: femur lacking spines and tibia with a longitudinal row of spines on the retrolateral surface.

**Redescription.** Carapace yellowish or white. Sternum shield-shaped. Opisthosoma gray, ovoid, covered with short hairs. Male pedipalpal patella with one short spine dorso-distally; tibia with trichobothria dorsally; cymbium with strong, thorny spine distally; bulb yellowish, ovoid, with two appendages inserted ventrally, median apophysis chitinous, conductor membranous, median apophysis and conductor absent in some species, embolus transparent, membranous. Female genital area covered with short hairs. Vulva with a pair of spermathecae and sperm ducts, spermathecae twisted and weakly sclerotized.

**Distribution.** Greece, Turkey, Georgia, Azerbaijan, Vietnam and China.

### *Leptonetela chakou* Wang & Li sp. nov. Figures 4-5, 97

**Figure 4:**
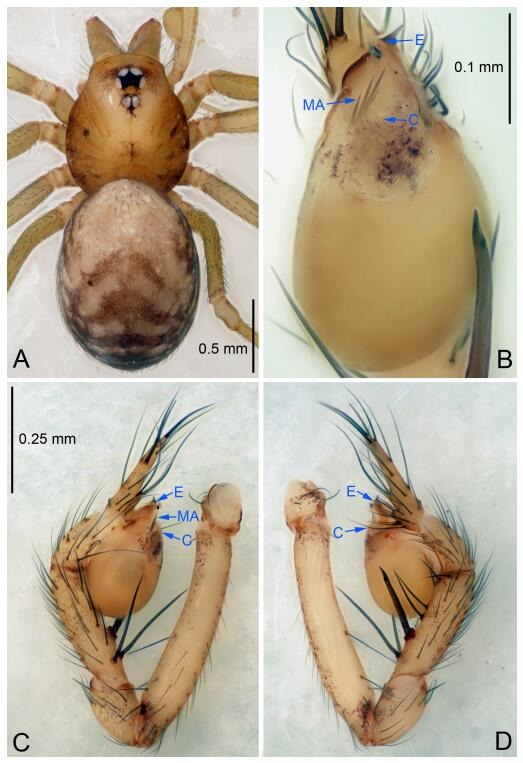
*Leptonetela chakou* sp. nov., holotype male

**Figure 5:**
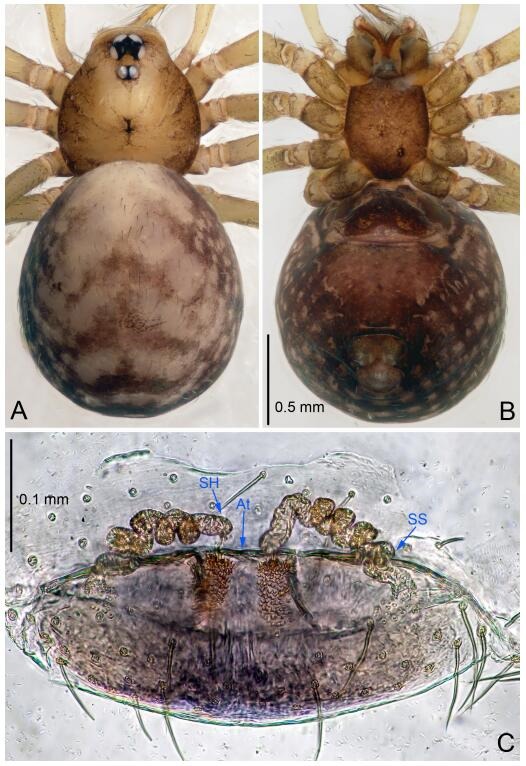
*Leptonetela chakou* sp. nov., one of the paratype females

**Figure 97:**
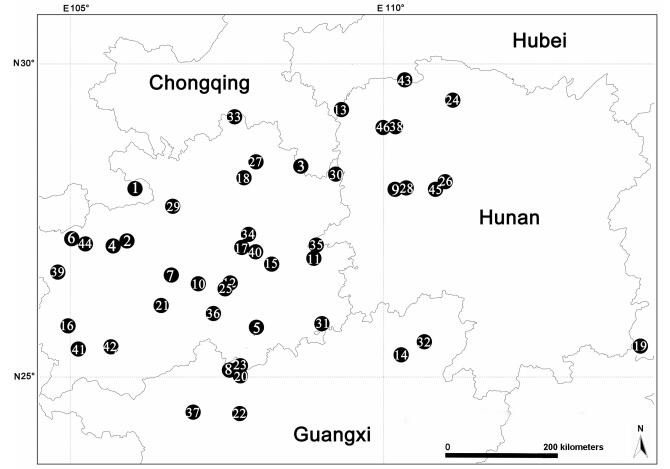
Locality records for forty-six new species of *Leptonetela* in China

**Type material. Holotype:** male (IZCAS), Chakou Cave, N27.93°, E106.14°, Shalang, Shibao Town, Gulin County, Luzhou City, Sichuan Province, China, 20 April 2014, Y. Li, H. Zhao & Y. Lin leg. **Paratypes:** 1 male and 3 females, same data as holotype.

**Etymology.** The specific name refers to the type locality; noun.

**Diagnosis.** This new species is similar to *L. dao* Wang & Li **sp. nov.**, *L. grandispina* Lin & Li, 2010, *L. liuzhai* Wang & Li **sp. nov.**, *L. pentakis* Lin & Li, 2010, and *L. shuilian* Wang & Li **sp. nov.**, but can be distinguished by the male pedipalpal tibia with 5 spines retrolaterally, the basal spine strong, conspicuous and with a bifurcate tip (Figure 4D) (6 short spines, with spine Ⅱ largest in *L. grandispina*, 5 slender spines in *L. dao* Wang & Li **sp. nov.**, *L. liuzhai* Wang & Li **sp. nov.**, *L. pentakis* and *L. shuilian* Wang & Li **sp. nov.**); the median apophysis divided into 4 pine needle like structures (Figure 4B) (median apophysis divided into 2 pine needlelike structures a in *L. dao* Wang & Li **sp. nov.** and *L. shuilian* Wang & Li **sp. nov.**, 15 pine needlelike structures in *L. liuzhai* Wang & Li **sp. nov.**, and 5 pine needle like structures in *L. pentakis*); from *L. dao* Wang & Li **sp. nov.**, *L. grandispina*, *L. pentakis* by the conductor reduced in this new species (Figure 4B); from *L. liuzhai* Wang & Li **sp. nov.** by the cymbium 1.3 times longer than bulb (Figure 4C-D) (cymbium 2 times longer than bulb in *L. liuzhai* Wang & Li **sp. nov.** and *L. shuilian* Wang & Li **sp. nov.**).

**Description. Male (holotype).** Total length 2.25 (Figure 4A). Carapace 0.87 long, 0.87 wide. Opisthosoma 1.50 long, 1.00 wide. Carapace brown. Eyes six. Median groove, cervical grooves and radial furrows distinct. Clypeus 0.13 high. Opisthosoma gray, ovoid, with pigmented stripe. Leg measurements: Ⅰ 7.63 (2.05, 0.35, 2.35, 1.75, 1.13); Ⅱ 5.71 (1.63, 0.30, 1.60, 1.30, 0.88); Ⅲ 4.73 (1.25, 0.30, 1.13, 1.20, 0.85); Ⅳ 6.30 (1.75, 0.35, 1.75, 1.45, 1.00). Male pedipalp (Figure 4C-D): tibia with 2 large spines prolaterally, and 5 spines retrolaterally, Ⅰ spine strong, conspicuous, tip bifurcated. Cymbium constricted medially, attaching to an earlobe-shaped process. Embolus triangular, bearing a basal tooth. Median apophysis sclerotized, divided into 4 pine needle like structures. Conductor membranous, reduced (Figure 4B).

**Female (one of the paratypes).** Similar to male in color and general features, but larger and with shorter legs. Total length 2.27 (Figure 5A-B). Carapace 0.88 long, 0.80 wide. Opisthosoma 1.50 long, 1.25 wide. Clypeus 0.12 high. Leg measurements: Ⅰ 5.83 (1.50, 0.35, 1.55, 1.38, 1.05); Ⅱ 4.43 (1.13, 0.30, 1.25, 1.00, 0.75); Ⅲ 3.62 (1.00, 0.25, 1.00, 0.75, 0.62); Ⅳ 4.96 (1.38, 0.35, 1.35, 1.13, 0.75). Vulva (Figure 5C): spermathecae coiled, atrium fusiform.

**Distribution.** China (Sichuan).

### *Leptonetela dao* Wang & Li sp. nov. Figures 6-7, 97

**Figure 6:**
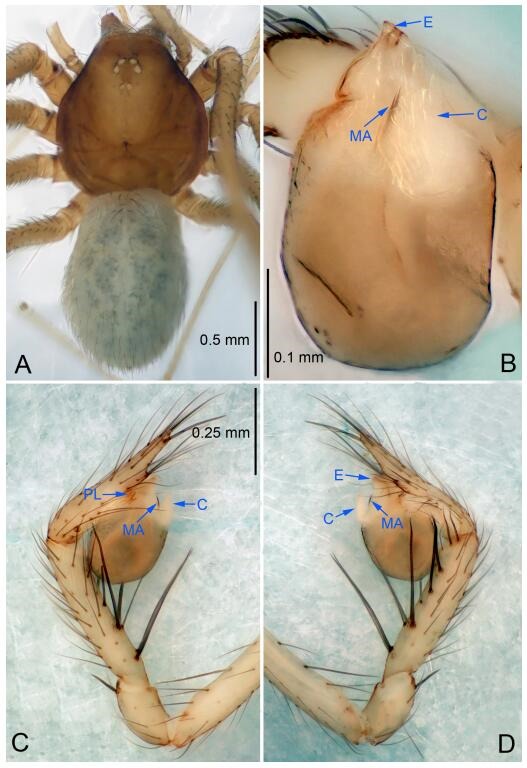
*Leptonetela dao* sp. nov., holotype male

**Figure 7:**
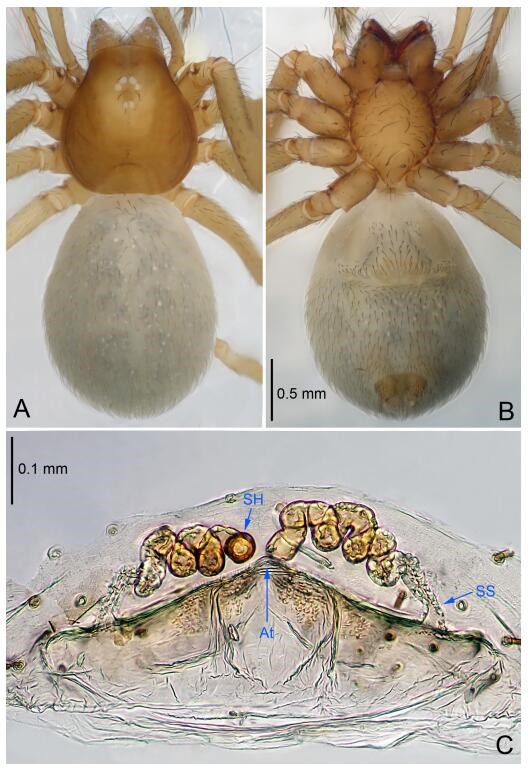
*Leptonetela dao* sp. nov., one of the paratype females

**Type material. Holotype:** male (IZCAS), Dao Cave, N27.19°, E105.06°, Shuanglong, Salaxi County, Bijie City, Guizhou Province, China, 18 November 2011, H. Chen & Z. Zha leg. **Paratypes:** 1 male and 20 females, same data as holotype; 5 males and 7 females, Shanlanqiao Cave, N26.28°, E106.04°, Shanlanqiao, Qianyanqiao Town, Anshun City, Guizhou Province, China, 4 November 2011, H. Chen & Z. Zha leg.

**Etymology.** The specific name refers to the type locality; noun.

**Diagnosis.** This new species is similar to *L. chakou* Wang & Li **sp. nov.**, *L. grandispina* Lin & Li, 2010, *L. liuzhai* Wang & Li **sp. nov.** *L. pentakis* Lin & Li, 2010, and *L. shuilian* Wang & Li **sp. nov.**, but can be separated from *L. chakou* Wang & Li **sp. nov.**, *L. grandispina*, *L. liuzhai* Wang & Li **sp. nov.** and *L. pentakis* by median apophysis divided into 2 pine needlelike (Figure 6B) (median apophysis divided into 4 pine needle like structures in *L. chakou* Wang & Li **sp. nov.** and *L. grandispina*, 15 pine needlelike structures in *L. liuzhai* Wang & Li **sp. nov.**, and 5 pine needlelike structures in *L. pentakis*); from *L. chakou* Wang & Li **sp. nov.**, *L. grandispina* by the tibial spines slender (Figure 6D) (the tibia Ⅰ spine in *L. chakou* Wang & Li **sp. nov.** and Ⅱ spines in *L. grandispina* strong); from *L. chakou* Wang & Li **sp. nov.**, *L. pentakis* by the cymbium not constricted medially (Figure 6C); from *L. liuzhai* Wang & Li **sp. nov.** and *L. shuilian* Wang & Li **sp. nov.** by the cymbium 1.2 times longer than bulb (Figure 6C-D) (cymbium 2 times longer than bulb in *L. liuzhai* Wang & Li **sp. nov.** and *L. shuilian* Wang & Li **sp. nov.**).

**Description. Male (holotype).** Total length 2.28 (Figure 6A). Carapace 1.15 long, 1.03 wide. Opisthosoma 1.28 long, 0.93 wide. Carapace brown. Eyes six, reduced to white vestiges. Median groove, cervical grooves and radial furrows distinct. Clypeus 0.15 high. Opisthosoma gray, ovoid. Leg measurements: Ⅰ 10.36 (2.76, 0.40, 3.24, 2.40, 1.56); Ⅱ 8.72 (2.44, 0.36, 2.60, 1.72, 1.60); Ⅲ 6.20 (2.04, 0.32, 1.52, 1.40, 0.92); Ⅳ 8.80 (2.56, 0.40, 2.60, 2.04, 1.20). Male pedipalp (Figure 6C-D): tibia with 5 slender spines prolaterally and 5 slender spines retrolaterally, with Ⅰ spine longest. Cymbium not wrinkled, earlobe-shaped process small. Embolus triangular, prolateral lobe small, oval. Median apophysis sclerotized, divided into 2 pine needle like structures. Conductor broad, C shaped in ventral view (Figure 6B).

**Female (one of the paratypes).** Similar to male in color and general features, but larger and with longer legs. Total length 2.76 (Figure 7A-B). Carapace 1.13 long, 1.10 wide. Opisthosoma 1.65 long, 1.40 wide. Clypeus 0.13 high. Leg measurements: Ⅰ 11.36 (3.00, 0.40, 3.60, 2.60, 1.76); Ⅱ 9.08 (2.64, 0.36, 2.80, 1.88, 1.40); Ⅲ 7.44 (2.24, 0.36, 1.96, 1.64, 1.24); Ⅳ 9.68 (2.80, 0.40, 3.00, 2.08, 1.40). Vulva (Figure 7C): spermathecae coiled, atrium triangular, anterior margin of atrium with short hairs.

**Distribution.** China (Guizhou).

### *Leptonetela liuzhai* Wang & Li sp. nov. Figures 8-9, 97

**Figure 8:**
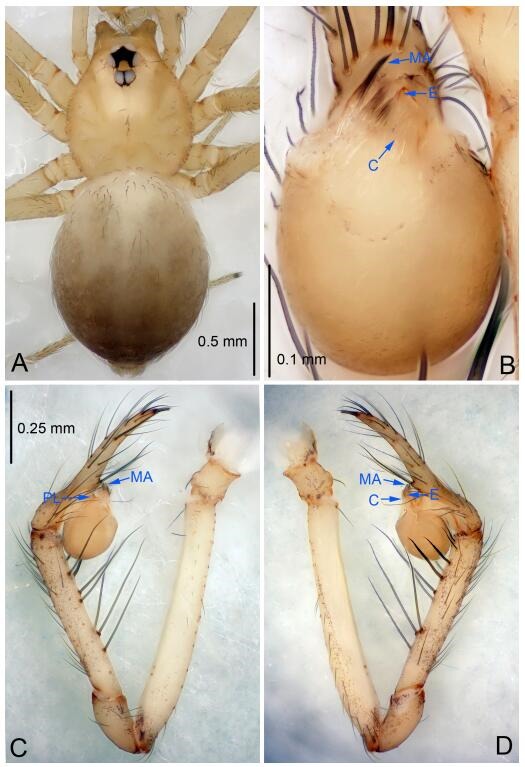
*Leptonetela liuzhai* sp. nov., holotype male

**Figure 9:**
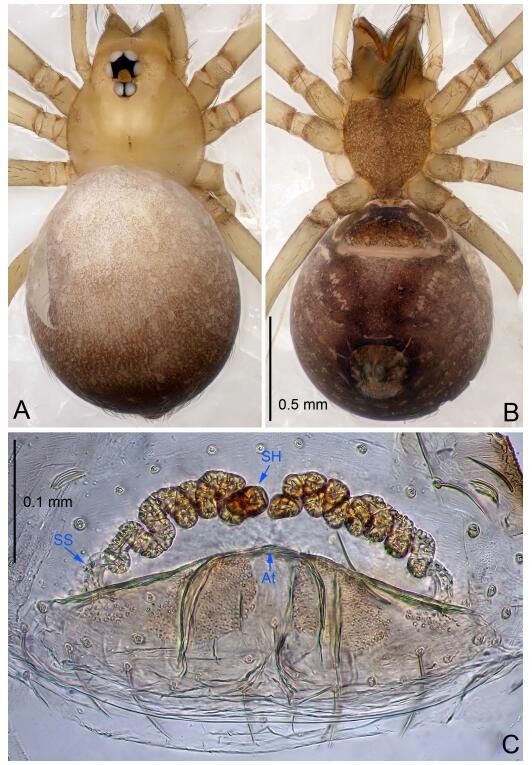
*Leptonetela liuzhai* sp. nov., one of the paratype females

**Type material. Holotype:** male (IZCAS), nameless Cave, N25.27°, E107.43°, Longli, Liuzhai Town, Nandan County, Hechi City, Guangxi Zhuang Autonomous Region, China, 29 January 2015, Y. Li & Z. Chen leg. **Paratypes:** 2 males and 6 females, same data as holotype.

**Etymology.** The specific name refers to the type locality; noun.

**Diagnosis.** This new species is similar to *L. chakou* Wang & Li **sp. nov.**, *L. dao* Wang & Li **sp. nov.**, *L. grandispina* Lin & Li, 2010, *L. pentakis* Lin & Li, 2010, and *L. shuilian* Wang & Li **sp. nov.** but can be separated from *L. chakou* Wang & Li **sp. nov.**, *L. dao* Wang & Li **sp. nov.**, *L. grandispina*, and *L. pentakis* by the male pedipalpal cymbium double the length of bulb, median apophysis divided into 15 pine needle-like structures (Figure 8B) (cymbium not double the length of bulb in *L. chakou* Wang & Li **sp. nov.**, *L. dao* Wang & Li **sp. nov.**, *L. grandispina*, and *L. pentakis*; median apophysis with 4 pine needlelike structures in *L. chakou* Wang & Li **sp. nov.** and *L. grandispina*, 2 pine needlelike structures in *L. dao* Wang & Li **sp. nov.** and *L. shuilian* Wang & Li **sp. nov.**, and 5 pine needlelike structures in *L. pentakis*); from *L. chakou* Wang & Li **sp. nov.**, and *L. grandispina* by the tibial spines slender (Figure 8D) (Ⅰ tibial spine in *L. chakou* Wang & Li **sp. nov.** and Ⅱ spines in *L. grandispina* strong); from *L. chakou* Wang & Li **sp. nov.**, and *L. pentakis* by the cymbium not constricted medially in this new species (Figure 8C-D).

**Description. Male (holotype).** Total length 2.25 (Figure 8A). Carapace 1.00 long, 0.88 wide. Opisthosoma 1.35 long, 1.10 wide. Carapace yellowish. Ocular area with a pair of setae, eyes six. Median groove needle-shaped, cervical grooves and radial furrows distinct. Clypeus 0.15 high. Opisthosoma gray, ovoid. Leg measurements: Ⅰ 8.30 (2.25, 0.25, 2.35, 1.95, 1.50); Ⅱ 6.68 (1.88, 0.25, 2.00, 1.55, 1.00); Ⅲ 5.70 (1.63, 0.20, 1.62, 1.35, 0.90); Ⅳ 7.49 (2.13, 0.25, 2.13, 1.85, 1.13). Male pedipalp (Figure 8C-D): tibia with 5 long spines prolaterally and 5 spines retrolaterally, with tibia Ⅰ spine longest. Cymbium not wrinkled, earlobe-shaped process small, cymbium double the length of bulb. Embolus triangular, prolateral lobe reduced. Median apophysis sclerotized, divided into 15 pine needlelike structures. Conductor reduced (Figure 8B).

**Female (one of the paratypes).** Similar to male in color and general features, but larger and with shorter legs. Total length 2.50 (Figure 9A-B). Carapace 1.50 long, 0.88 wide. Opisthosoma 1.13 long, 1.38 wide. Clypeus 0.13 high. Leg measurements: Ⅰ 7.30 (2.00, 0.25, 2.25, 1.75, 1.05); Ⅱ 5.51 (1.63, 0.20, 1.55, 1.25, 0.88); Ⅲ 4.76 (1.38, 0.25, 1.25, 1.13, 0.75); Ⅳ 6.50 (1.87, 0.25, 1.88, 1.50, 1.00). Vulva (Figure 9C): spermathecae coiled, atrium fusiform.

**Distribution.** China (Guangxi).

### *Leptonetela shuilian* Wang & Li sp. nov. Figures 10-11, 97

**Figure 10:**
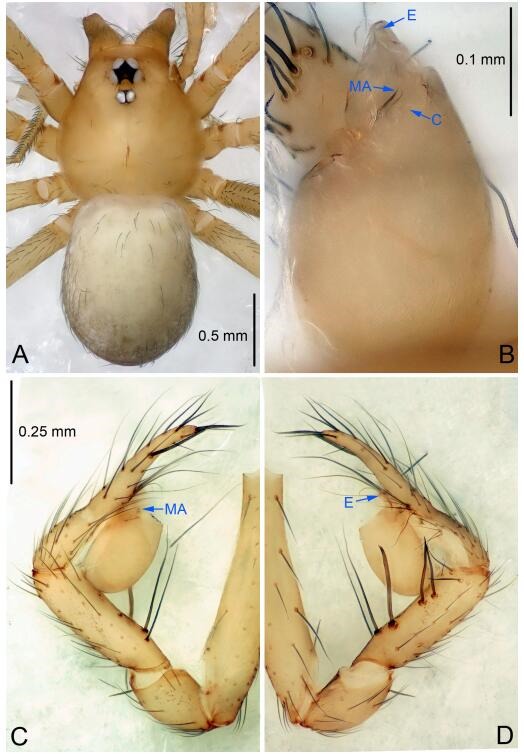
*Leptonetela shuilian* sp. nov., holotype male

**Figure 11:**
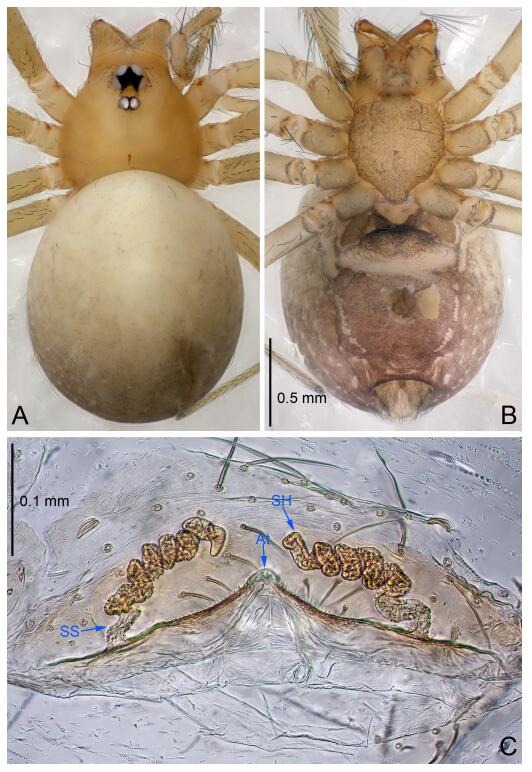
*Leptonetela shuilian* sp. nov., one of the paratype females

**Type material. Holotype:** male (IZCAS), Shuilian Cave, N24.43°, E106.97°, Pingle, Fengshan County, Hechi City, Guangxi Zhuang Autonomous Region, China, 22 March 2015, Y. Li & Z. Chen leg. **Paratypes:** 6 males and 4 females, same data as holotype.

**Etymology.** The specific name refers to the type locality; noun.

**Diagnosis.** This new species is similar to *L. chakou* Wang & Li **sp. nov.**, *L. dao* Wang & Li **sp. nov.**, *L. grandispina* Lin & Li, 2010, *L. pentakis* Lin & Li, 2010, and *L. liuzhai* Wang & Li **sp. nov.** but can be separated from *L. chakou* Wang & Li **sp. nov.**, *L. dao* Wang & Li **sp. nov.**, *L. grandispina* Lin & Li, 2010, *L. pentakis* Lin & Li, 2010 by the male pedipalpal cymbium double the length of bulb; from *L. chakou* Wang & Li **sp. nov.**, *L. grandispina*, *L. liuzhai* Wang & Li **sp. nov.** and *L. pentakis* by the median apophysis divided into 2 pine needlelike structures in *L. chakou* Wang & Li **sp. nov.** (Figure 10B) (median apophysis divided into 4 pine needlelike structures in *L. chakou* Wang & Li **sp. nov.** and *L. grandispina*, 15 pine needlelike structures in *L. liuzhai* Wang & Li **sp. nov.**, and 5 pine needlelike structures in *L. pentakis*); from *L. chakou* Wang & Li **sp. nov.** and *L. grandispina* by the tibial spines slender (Figure 10D) (Ⅰ tibial spine in *L. chakou* Wang & Li **sp. nov.** and Ⅱ spines in *L. grandispina* strong); from *L. chakou* Wang & Li **sp. nov.** and *L. pentakis* by the cymbium not constricted medially in this new species.

**Description. Male (holotype).** Total length 2.25 (Figure 10A). Carapace 1.13 long, 1.00 wide. Opisthosoma 1.25 long, 0.90 wide. Carapace yellow. Ocular area with a pair of setae, eyes six. Median groove needle-shaped, cervical grooves and radial furrows indistinct. Clypeus 0.12 high. Opisthosoma gray, ovoid. Leg measurements: Ⅰ -(2.63, -, 2.88, 2.35, 1.60); Ⅱ -(2.13, -, 2.25, 2.00, 1.10); Ⅲ -(1.88, -, 1.75, 1.50, 0.95); Ⅳ -(2.38, -, 2.38, 2.10, 1.25). Male pedipalp (Figure 10C-D): tibia with 3 long spines prolaterally, and 5 spines retrolaterally, with Ⅰ spine longest, tip bifurcated. Cymbium not wrinkled, earlobe-shaped process absent, cymbium double the length of bulb. Embolus spoon-shaped; prolateral lobe reduced. Median apophysis sclerotized, divided into 2 sharp pine needlelike structures. Conductor reduced (Figure 10B).

**Female (one of the paratypes).** Similar to male in color and general features, but smaller and with shorter legs. Total length 2.10 (Figure 11A-B). Carapace 1.00 long, 0.85 wide. Opisthosoma 1.50 long, 1.13 wide. Clypeus 0.10 high. Leg measurements: Ⅰ 7.30 (1.75, 0.35, 2.25, 1.70, 1.25); Ⅱ 5.33 (1.40, 0.30, 1.63, 1.00, 1.00); Ⅲ 5.01 (1.25, 0.25, 1.38, 1.25, 0.88); Ⅳ 6.48 (1.60, 0.30, 1.88, 1.60, 1.10). Vulva (Figure 11C): spermathecae coiled, apical part free, atrium semicircular, anterior margin of the atrium with short hairs.

**Distribution.** China (Guangxi).

### *Leptonetela chenjia* Wang & Li sp. nov. Figures 12-13, 97

**Figure 12:**
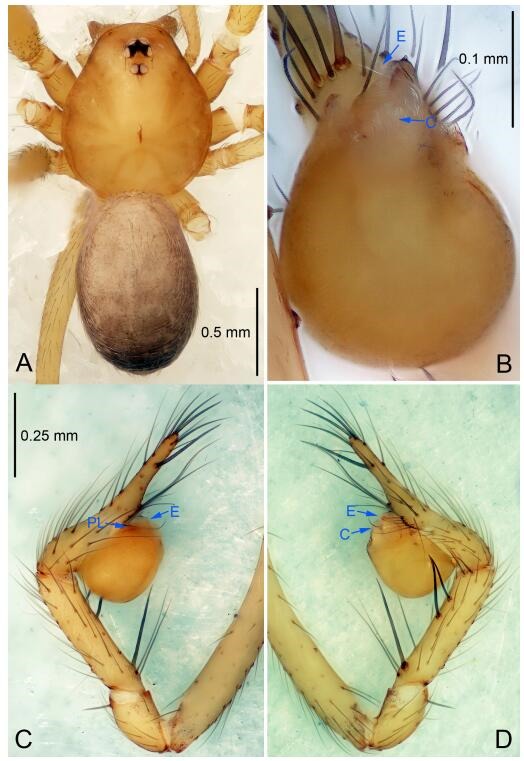
*Leptonetela chenjia* sp. nov., holotype male

**Figure 13:**
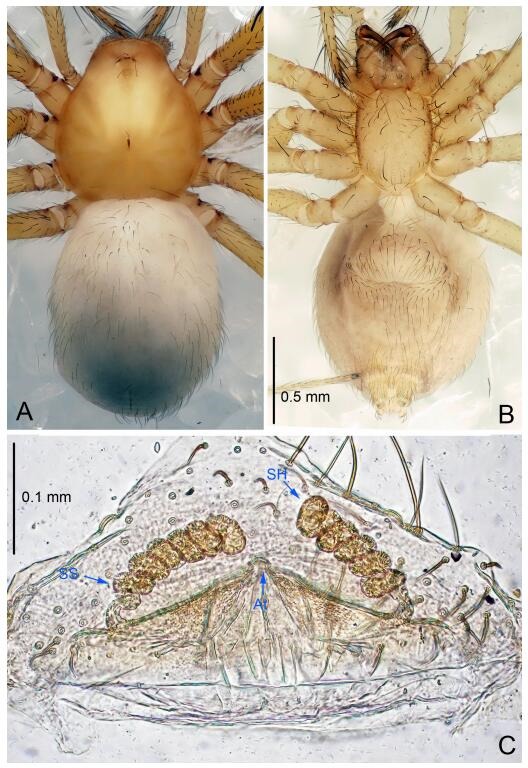
*Leptonetela chenjia* sp. nov., one of the paratype females

**Type material. Holotype:** male (IZCAS), Chenjia Cave, N28.38°, E108.67°, Tianba, Songtao County, Tongren City, Guizhou Prvince, China, 9 March 2013, H. Zhao & J. Liu leg. **Paratypes:** 1 male and 2 females, same data as holotype.

**Etymology.** The specific name refers to the type locality; noun.

**Diagnosis.** This new species is similar to *L. anshun* Lin & Li, 2010, *L. suae* Lin & Li, 2010, *L. tongzi* Lin & Li, 2010, *L. meitan* Lin & Li, 2010, *L. liangfeng* Wang & Li **sp. nov.**, and *L. sanyan* Wang & Li **sp. nov.**, but can be distinguished by the male pedipalal tibia Ⅰ spine far apart from the other 4 spines (Figure 12D), conductor reduced (Figure 12B) (tibial spines Ⅰ bifurcated symmetrically in *L. anshun*; conductor tip bifurcated in *L. anshun*, bamboo leaf-shaped in *L. sanyan* Wang & Li **sp. nov.**, and *L. tongzi*; thin, triangular in *L. suae* and *L. meitan*, and C shaped in *L. liangfeng* Wang & Li **sp. nov.**); is also similar to *L. huoyan* Wang & Li **sp. nov.**, but can be distinguished by the absent median apophysis, reduced conductor (Figure 12B) (median apophysis present, slightly sclerotized, index finger like, conductor broad, semicircular in *L. huoyan* Wang & Li **sp. nov.**).

**Description. Male (holotype).** Total length 2.50 (Figure 12A). Carapace 1.25 long, 0.95 wide. Opisthosoma 1.25 long, 0.88 wide. Carapace yellow. Ocular area with a pair of setae, six eyes. Median groove needle-shaped, cervical grooves and radial furrows distinct. Clypeus 0.15 high. Opisthosoma pale brown, ovoid, with pigmented stripe. Leg measurements: Ⅰ 10.44 (2.60, 0.37, 3.05, 2.50, 1.62); Ⅱ 7.84 (2.25, 0.35, 2.25, 1.87, 1.12); Ⅲ 6.41 (1.50, 0.32, 1.87, 1.62, 1.10); Ⅳ 8.59 (2.50, 0.35, 2.37, 2.12, 1.25). Male pedipalp (Figure 12C-D): tibia with 3 long spines prolaterally, 5 spines retrolaterally, with Ⅰ spine longest, far apart from others. Cymbium not wrinkled. Embolus triangular, prolateral lobe oval. Median apophysis absent. Conductor reduced (Figure 12B).

**Female (one of the paratypes).** Similar to male in color and general features, but smaller and with shorter legs. Total length 2.25 (Figure 13A-B). Carapace 0.87 long, 0.80 wide. Opisthosoma 1.37 long, 1.12 wide. Clypeus 0.12 high. Leg measurements: Ⅰ 7.89 (2.12, 0.37, 2.25, 1.85, 1.30); Ⅱ 6.19 (1.62, 0.32, 1.75, 1.40, 1.10); Ⅲ 5.10 (1.45, 0.30, 1.25, 1.20, 0.90); Ⅳ 6.76 (1.80, 0.35, 1.87, 1.62, 1.12). Vulva (Figure 13C): spermathecae coiled, apical part coiled, atrium triangular.

**Distribution.** China (Guizhou).

### *Leptonetela liangfeng* Wang & Li sp. nov. Figures 14-15, 97

**Figure 14:**
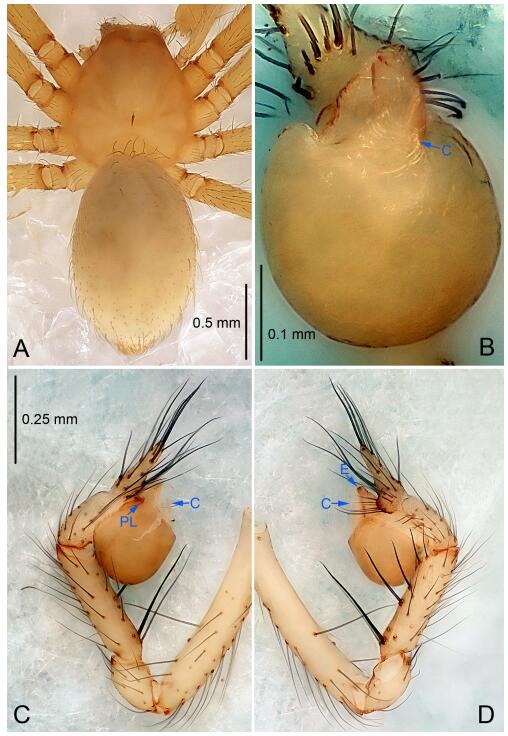
*Leptonetela liangfeng* sp. nov., holotype male

**Figure 15:**
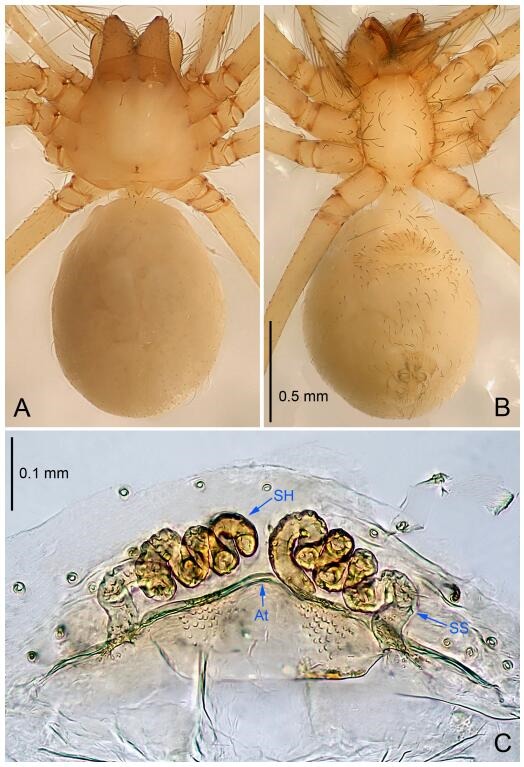
*Leptonetela liangfeng* sp. nov., one of the paratype females

**Type material. Holotype:** male (IZCAS), Liangfeng Cave, N28.32°, E107.84°, Tian, Fengle Town, Wuchuan County, Zunyi City, Guizhou Province, China, 7 August 2012, H. Zhao leg. **Paratypes** : 1 male and 2 females, same data as holotype.

**Etymology.** The specific name refers to the type locality; noun.

**Diagnosis.** This new species is similar to *L. anshun* Lin & Li, 2010, *L. suae* Lin & Li, 2010, *L. tongzi* Lin & Li, 2010, *L. meitan* Lin & Li, 2010, *L. chenjia* Wang & Li **sp. nov.**, and *L. sanyan* Wang & Li **sp. nov.**, but can be distinguished by the male pedipalpal bulb conductor C shaped (Figure 14B) (conductor tip bifurcated in *L. anshun*, bamboo leaf-shaped in *L. sanyan* Wang & Li **sp. nov.**, and *L. tongzi*; thin, triangular in *L. suae* and *L. meitan*, reduced in *L. chenjia* Wang & Li **sp. nov.**); from *L. anshun* by the tibia Ⅰ spine slender not bifurcated (Figure 14D) (tibia Ⅰ spine symmetrically bifurcated in *L. anshun*).

**Description. Male (holotype).** Total length 2.28 (Figure 14A). Carapace 0.93 long, 0.88 wide. Opisthosoma 1.35 long, 0.88 wide. Carapace yellow. Eyes absent. Median groove needle-shaped, cervical grooves and radial furrows indistinct. Clypeus 0.13 high. Opisthosoma yellowish, ovoid. Leg measurements: Ⅰ 9.47 (2.59, 0.43, 2.88, 2.25, 1.32); Ⅱ 8.61 (2.23, 0.32, 2.60, 2.05, 1.41); Ⅲ 7.38 (2.05, 0.43, 2.08, 1.55, 1.27); Ⅳ 8.81 (2.51, 0.38, 2.17, 2.28, 1.47). Male pedipalp (Figure 14C-D): tibia with 4 long setae prolaterally and 5 spines retrolaterally, tibia Ⅰ spine longest. Cymbium constricted medially, attached to an earlobe-shaped process. Embolus triangular, prolateral lobe oval. Median apophysis absent. Conductor C shaped in ventral view (Figure 14B).

**Female (one of the paratypes).** Similar to male in color and general features, but smaller and with shorter legs. Total length 2.14 (Figure 15A-B). Carapace 0.88 long, 0.73 wide. Opisthosoma 1.36 long, 0.95 wide. Clypeus 0.13 high. Leg measurements: Ⅰ 7.44 (1.98, 0.38, 2.03, 1.77, 1.28); Ⅱ 7.01 (1.88, 0.37, 1.98, 1.55, 1.23); Ⅲ 5.78 (1.33, 0.25, 1.75, 1.42, 1.03); Ⅳ 7.59 (2.03, 0.33, 2.18, 1.79, 1.26). Vulva (Figure 15C): spermathecae coiled, atrium triangular.

**Distribution.** China (Guizhou).

### *Leptonetela sanyan* Wang & Li sp. nov. Figures 16-17, 97

**Figure 16:**
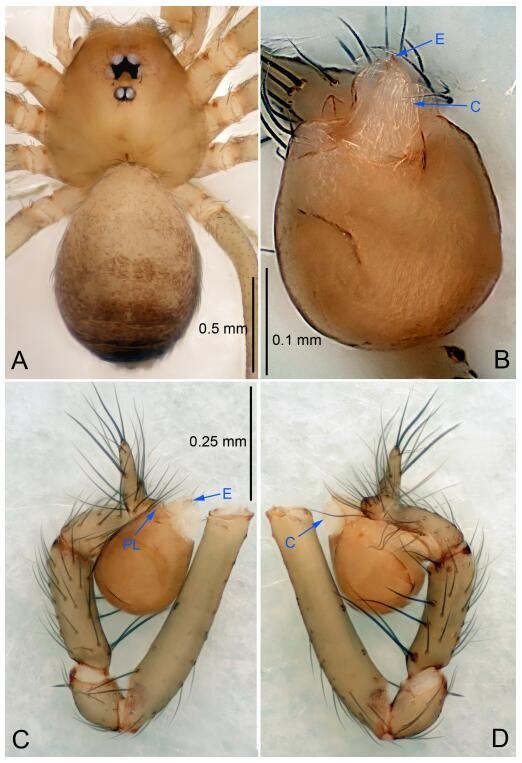
*Leptonetela sanyan* sp. nov., holotype male

**Figure 17:**
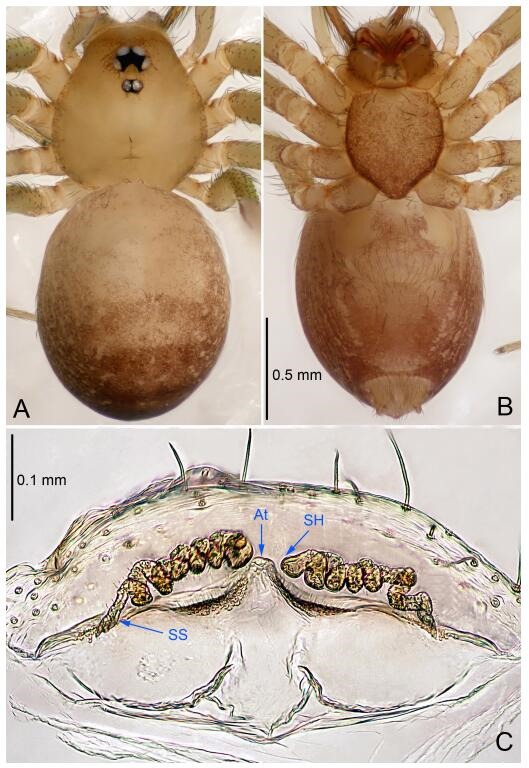
*Leptonetela sanyan* sp. nov., one of the paratype females

**Type material. Holotype:** male (IZCAS), Sanyan Cave, N29.15°, E107.60°, Heyi, Yangxi Town, Daozhen County, Guizhou Province, China, 30 May 2011, Z. Zha leg. **Paratypes:** 1 male and 2 females, same data as holotype.

**Etymology.** The specific name refers to the type locality; noun.

**Diagnosis.** This new species is similar to *L. anshun* Lin & Li, 2010, *L. suae* Lin & Li, 2010, *L. tongzi* Lin & Li, 2010, *L. meitan* Lin & Li, 2010, *L. chenjia* Wang & Li **sp. nov.**, and *L. liangfeng* Wang & Li **sp. nov.**, but can be separated from all above except *L. tongzi* by in the male conductor C shaped in this new species (Figure 16B) (conductor tip bifurcated in *L. anshun*, C shaped in *L. liangfeng* Wang & Li **sp. nov.**, thin, triangular in *L. suae* and *L. meitan,* reduced in *L. chenjia* Wang & Li **sp. nov.**); from *L. tongzi* by in the female atrium triangular, anterior margin of the atrium undulate (Figure 17C) (atrium fusiform, anterior margin of the atrium with pointed process medially in *L. tongzi*).

**Description. Male (holotype).** Total length 1.78 (Figure 16A). Carapace 0.83 long, 0.83 wide. Opisthosoma 1.00 long, 0.75 wide. Carapace yellowish. Ocular area with a pair of setae, six eyes. Median groove needle-shaped, pale brown. Cervical grooves and radial furrows indistinct. Clypeus 0.13 high, slightly sloped anteriorly. Opisthosoma yellow, ovoid, with pigmented stripe. Leg measurements: Ⅰ 7.08 (2.00, 0.33, 2.15, 1.75, 1.15); Ⅱ 6.09 (1.68, 0.30, 1.73, 1.38, 1.00); Ⅲ 4.84 (1.38, 0.28, 1.25, 1.13, 0.80); Ⅳ 6.28 (1.75, 0.30, 1.78, 1.50, 0.95). Male pedipalp (Figure 16C-D): tibia with 1 long spine prolaterally, 5 spines retrolaterally, with the basal spine longest. Cymbium constricted medially, attaching an earlobe-shaped process. Embolus triangular, prolateral lobe absent. Median apophysis absent. Conductor bamboo leaf-shaped in ventral view (Figure 16B).

**Female (one of the paratypes).** Similar to male in color and general features, but larger and with shorter legs. Total length 2.03 (Figure 17A-B). Carapace 0.80 long, 0.75 wide. Opisthosoma 1.25 long, 0.93 wide. Clypeus 0.13 high. Leg measurements: Ⅰ 6.92 (1.88, 0.33, 2.00, 1.58, 1.13); Ⅱ 5.36 (1.50, 0.30, 1.53, 1.15, 0.88); Ⅲ 4.44 (1.20, 0.28, 1.13, 1.05, 0.78); Ⅳ 5.94 (1.73, 0.30, 1.58, 1.38, 0.95). Vulva (Figure 17C): spermathecae coiled, atrium triangular, anterior margin of the atrium undulate. Short hairs modified spermathecae, sperm ducts, and anterior margin of atrium.

**Distribution.** China (Guizhou).

### *Leptonetela wangjia* Wang & Li sp. nov. Figures 18-19, 97

**Figure 18:**
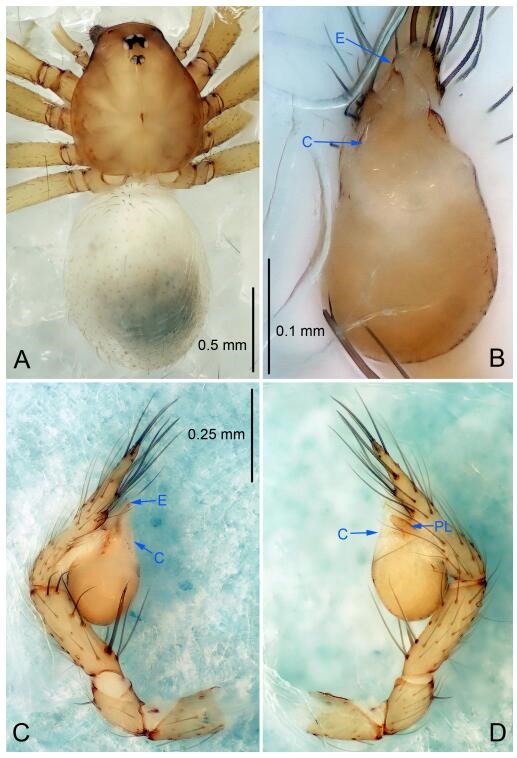
*Leptonetela wangjia* sp. nov., holotype male

**Figure 19:**
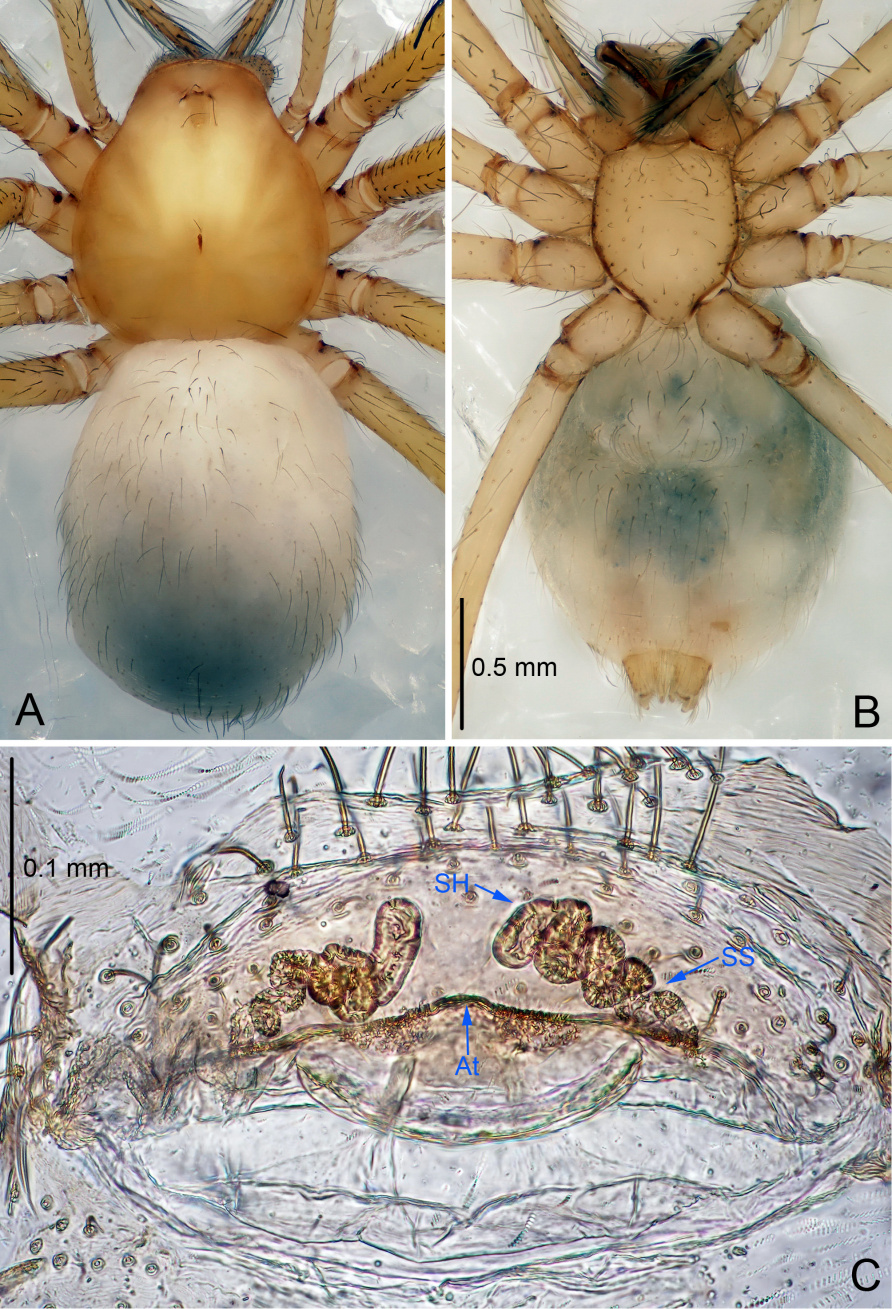
*Leptonetela wangjia* sp. nov., paratype female

**Type material. Holotype:** male (IZCAS), Wangjia Cave, N26.98°, E107.94°, Gaoqi, Nongchang Town, Huangpin County, Guizhou Province, China, 4 March 2012, H. Zhao & J. Liu leg. **Paratype:** 1 female, same data as holotype.

**Etymology.** The specific name refers to the type locality; noun.

**Diagnosis.** This new species is similar to *L. danxia* Lin & Li, 2010, and *L. yaoi* wang & Li, 2011, but can be distinguished by the reduced pedipalpal bulb conductor (Figure 18B) (conductor C shaped in *L. danxia*, bamboo leaf-shaped in *L. yaoi*), from *L. yaoi* by the tibia Ⅰ spine slender, asymmetrically bifurcated (Figure 18C) (tibia Ⅰ spine strong in *L. yaoi*); from *L. danxia* by the unwrinkled cymbium (Figure 18C-D) (cymbium constricted and wrinkled at 1/3 in *L. danxia*).

**Description. Male (holotype).** Total length 2.13 (Figure 18A). Carapace 0.88 long, 0.80 wide. Opisthosoma 1.00 long, 0.88 wide. Carapace yellow. Ocular area with a pair of setae, six eyes. Median groove needle-shaped, cervical grooves and radial furrows distinct. Clypeus 0.13 high. Opisthosoma gray, ovoid. Leg measurements: Ⅰ -(1.88, 0.25, -, -, -); Ⅱ 5.66 (1.63, 0.25, 1.63, 1.25, 0.90); Ⅲ 4.71 (1.25, 0.23, 1.25, 1.13, 0.85); Ⅳ 6.25 (1.75, 0.25, 1.75, 1.50, 1.00). Male pedipalp (Figure 18C-D): tibia with 7 long setae prolaterallly and 5 spines retrolaterally, Ⅰ spine slender, longest, asymmetrically bifurcated. Cymbium not wrinkled, earlobe-shaped process absent. Embolus triangular, prolateral lobe oval. Median apophysis absent. Conductor reduced (Figure 18B).

**Female.** Similar to male in color and general features, but larger and with longer legs. Total length 2.50 (Figure 19A-B). Carapace 1.00 long, 0.90 wide. Opisthosoma 1.50 long, 1.15 wide. Clypeus 0.20 high. Leg measurements: Ⅰ 9.26 (2.55, 0.38, 2.60, 2.10, 1.63); Ⅱ 8.25 (2.37, 0.38, 2.25, 1.90, 1.35); Ⅲ 6.90 (2.05, 0.35, 1.75, 1.65, 1.10); Ⅳ 7.86 (2.30, 0.38, 2.13, 1.75, 1.30). Vulva (Figure 19C): spermathecae coiled, atrium fusiform.

**Distribution.** China (Guizhou).

### *Leptonetela qiangdao* Wang & Li sp. nov. Figures 20-21, 97

**Figure 20:**
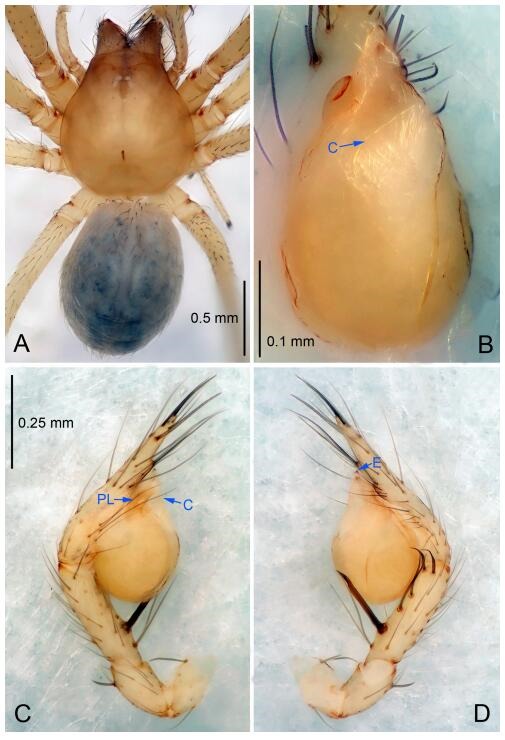
*Leptonetela qiangdao* sp. nov., holotype male

**Figure 21:**
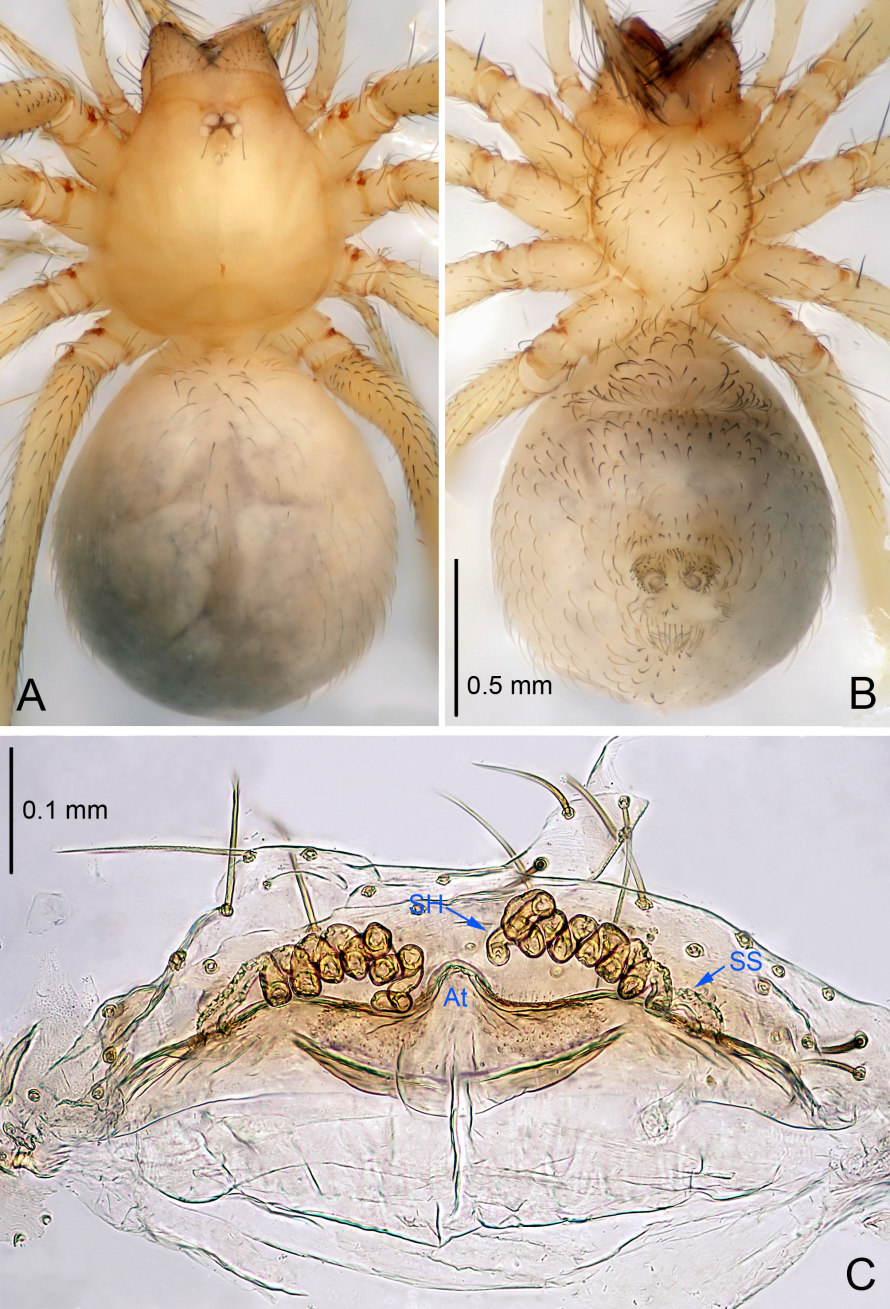
*Leptonetela qiangdao* sp. nov., one of the paratype females

**Type material. Holotype:** male (IZCAS), Qiangdao Cave, N25.83º, E109.04º, Guandong Town, Congjiang County, Qiandongnan Prefecture, Guizhou, China, 16 March 2013, H. Zhao & J. Liu leg. **Paratypes:** 3 females, same data as holotype.

**Etymology.** The specific name refers to the type locality; noun.

**Diagnosis.** This new species is similar to *L. furcaspina* Lin & Li, 2010, *L. langdong* Wang & Li **sp. nov.** and *L. dashui* Wang & Li **sp. nov.**, but can be distinguished by the male pedipalpal tibia Ⅰ spine strong (Figure 20D), conductor bamboo leaf-shaped in ventral view (Figure 20B) (tibia Ⅰ spine strong, asymmetrically bifurcated, conductor C shaped in *L. furcaspina*, tibia Ⅰ spine strong, tip curved, conductor reduced in *L. langdong* Wang & Li **sp. nov.**, tibia Ⅰ spine slender, Ⅱ Ⅲ spines curved basally, conductor C shaped in *L. dashui* Wang & Li **sp. nov.**).

**Description. Male (holotype).** Total length 1.80 (Figure 20A). Carapace 0.87 long, 0.90 wide. Opisthosoma 0.90 long, 0.75 wide. Carapace yellow. Four eyes, PME absent, ALE and PLE reduced to white points. Median groove needle-shaped, pale brown, cervical grooves and radial furrows indistinct. Clypeus 0.13 high. Opisthosoma whitish gray, ovoid, lacking distinctive pattern. Leg measurements: Ⅰ 7.88 (2.13, 0.33, 2.20, 1.92, 1.30); Ⅱ 6.41 (1.63, 0.30, 1.75, 1.58, 1.15); Ⅲ 5.40 (1.50, 0.30, 1.45, 1.23, 0.92); Ⅳ 7.43 (1.80, 0.33, 2.10, 1.72, 1.48). Male pedipalp (Figure 20C-D): tibia with 3 long setae prolaterally, 5 spines retrolaterally, with Ⅰ spine strong, longest, tip curved. Cymbium with no wrinkle medially, earlobe-shaped process small. Bulb with spoon-shaped embolus, prolateral lobe small. Median apophysis absent. Conductor bamboo leaf-shaped in ventral view (Figure 20B).

**Female (one of the paratypes).** Similar to male in color and general features, but larger and with shorter legs. Total length 2.25 (Figure 21A-B). Carapace 0.88 long, 0.80 wide. Opisthosoma 1.37 long, 1.13 wide. Clypeus 0.13 high. Leg measurements: Ⅰ 6.91 (2.10, 0.38, 1.88, 1.47, 1.08); Ⅱ 6.20 (1.70, 0.35, 1.75, 1.40, 1.00); Ⅲ 4.97 (1.37, 0.30, 1.25, 1.22, 0.83); Ⅳ 6.98 (1.88, 0.38, 1.92, 1.67, 1.13). Vulva (Figure 21C): spermathecae coiled, atrium trapezoidal, anterior margin of atrium undulate, covered with short hairs.

**Distribution.** China (Guizhou).

### *Leptonetela langdong* Wang & Li sp. nov. Figures 22-23, 97

**Figure 22:**
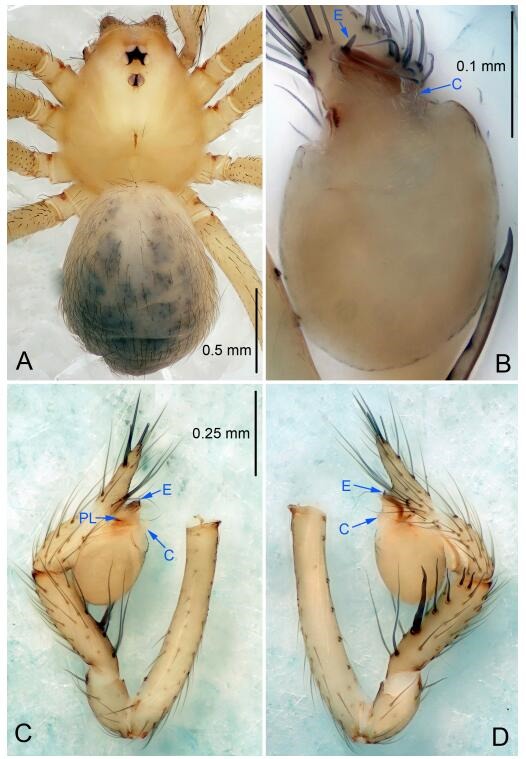
*Leptonetela langdong* sp. nov., holotype male

**Figure 23:**
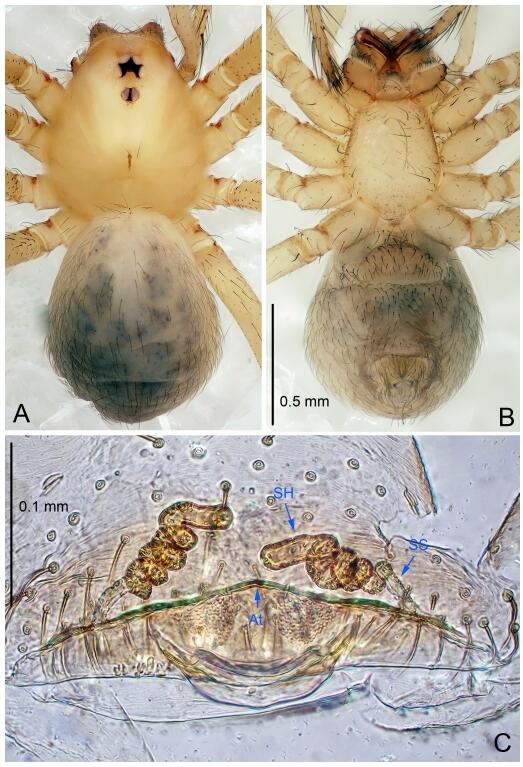
*Leptonetela langdong* sp. nov., one of the paratype females

**Type material. Holotype:** male (IZCAS), Menglonggong Cave, N27.07°, E107.76°, Langdong Village, Huangping County, Qiandongnan Prefecture, Guizhou Province, China, 3 March 2013, H. Zhao & J. Liu leg. **Paratypes:** 1 male and 2 females, same data as holotype.

**Etymology.** The specific name refers to the type locality; noun.

**Diagnosis.** This new species is similar to *L. furcaspina* Lin & Li, 2010, *L. qiangdao* Wang & Li **sp. nov.**, and *L. dashui* Wang & Li **sp. nov.**, but can be distinguished by the male pedipalpal tibia Ⅰ spine strong, tip curved (Figure 22D). conductor reduced (Figure 22B) (tibia Ⅰ spine strong, asymmetrically bifurcated, conductor C shaped in *L. furcaspina*, tibia Ⅰ spine strong, conductor bamboo leaf-shaped in *L. qiangdao* Wang & Li **sp. nov.**, tibia Ⅰ spine slender, Ⅱ Ⅲ spines curved basally, conductor C shaped in *L. dashui* Wang & Li **sp. nov.**).

**Description. Male (holotype):** total length 2.25 (Figure 22A). Carapace 1.25 long, 0.87 wide. Opisthosoma 1.13 long, 0.88 wide. Carapace yellowish. Six eyes. Median groove needle-shaped, cervical grooves and radial furrows indistinct. Clypeus 0.13 high. Opisthosoma gray, ovoid. Leg measurements: Ⅰ 8.91 (2.38, 0.35, 2.55, 2.13, 1.50); Ⅱ 7.23 (2.00, 0.35, 1.88, 1.75, 1.25); Ⅲ 6.22 (1.75, 0.34, 1.63, 1.50, 1.00); Ⅳ 8.05 (2.25, 0.35, 2.25, 1.90, 1.30). Male pedipalp (Figure 22C-D): femur with 6 spines ventrally, tibia with 3 long spines prolaterally, 1 long seta and 5 spines retrolaterally, with Ⅰ spine strong, tip curved. Cymbium constricted medially, attached to an earlobe-shaped process. Embolus triangular, bearing a tooth basally, prolateral lobe reduced. Median apophysis absent. Conductor reduced (Figure 22B).

**Female (one of the paratypes).** Similar to male in color and general features, but larger and with shorter legs. Total length 2.25 (Figure 23A-B). Carapace 1.10 long, 0.60 wide. Opisthosoma 1.25 long, 0.88 wide. Clypeus 0.15 high. Leg measurements: Ⅰ 7.48 (2.10, 0.38, 2.13, 1.62, 1.25); Ⅱ 6.14 (1.75, 0.38, 1.63, 1.38, 1.00); Ⅲ 5.18 (1.50, 0.35, 1.25, 1.20, 0.88); Ⅳ 6.86 (2.00, 0.38, 1.88, 1.50, 1.10). Vulva (Figure 23C): spermathecae coiled, atrium triangular.

**Distribution.** China (Guizhou).

### *Leptonetela dashui* Wang & Li sp. nov. Figures 24-25, 97

**Figure 24:**
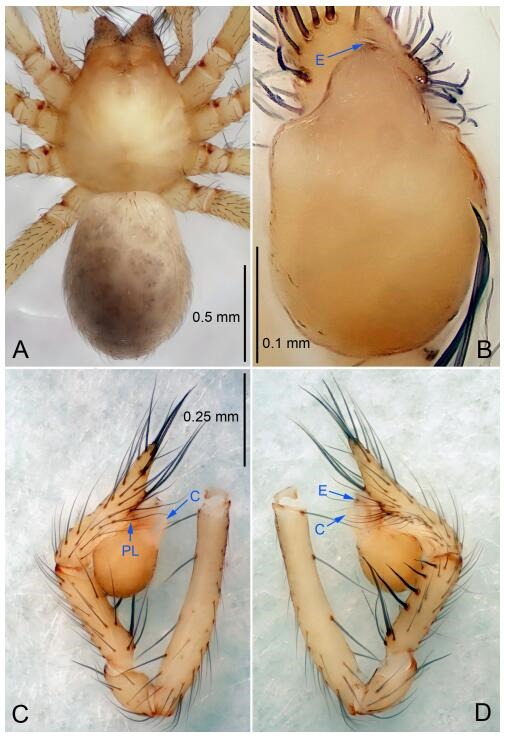
*Leptonetela dashui* sp. nov., holotype male

**Figure 25:**
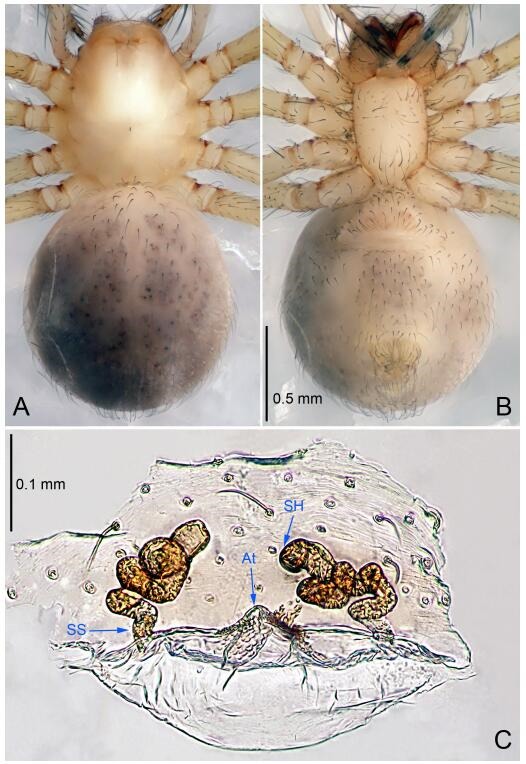
*Leptonetela dashui* sp. nov., one of the paratype females

**Type material. Holotype:** male (IZCAS), Dashui Cave, N26.61°, E106.61°, Shijicheng, Jinyang New Urban Area, Guiyang City, Guizhou Province, China, 18 June 2011, Z. Zha leg. **Paratypes:** 1 male and 2 females, same data as holotype.

**Etymology.** The specific name refers to the type locality; noun.

**Diagnosis.** This new species is similar to *L. furcaspina* Lin & Li, 2010, *L. qiangdao* Wang & Li **sp. nov.**, and *L. langdong* Wang & Li **sp. nov.**, but can be distinguished by the slender male pedipalpal tibia Ⅰ spine, Ⅱ Ⅲ spines curved basally (Figure 24D), conductor C shaped (Figure 24B), (tibia Ⅰ spine strong, tip curved, conductor reduced in *L. langdong* Wang & Li **sp. nov.**, tibia Ⅰ spine strong, asymmetrically bifurcated, conductor narrow and bifurcated in *L. furcaspina*, tibia Ⅰ spine strong, conductor bamboo leaf-shaped in *L. qiangdao* Wang & Li **sp. nov.**).

**Description. Male (holotype).** Total length 1.88 (Figure 24A). Carapace 0.88 long, 0.75 wide. Opisthosoma 1.00 long, 0.75 wide. Carapace yellowish. Eyes absent. Median groove, cervical grooves and radial furrows indistinct. Clypeus 0.13 high. Opisthosoma gray, ovoid. Leg measurements: Ⅰ 8.24 (2.25, 0.38, 2.43, 1.88, 1.30); Ⅱ 7.16 (1.98, 0.35, 2.00, 1.63, 1.20); Ⅲ 6.06 (1.75, 0.33, 1.60, 1.50, 0.88); Ⅳ 7.69 (2.13, 0.38, 2.08, 1.85, 1.25). Male pedipalp (Figure 24C-D): femur with 4 spines ventrally, tibia with 2 long setae prolaterally, 2 long setae and 5 slender spines retrolaterally, the spines equally strong, spines Ⅰ longest. Cymbium not wrinkled, earlobe-shaped process small. Embolus triangular, prolateral lobe reduced. Median apophysis absent. Conductor C shaped in ventral view (Figure 24B).

**Female (one of the paratypes).** Similar to male in color and general features, but larger and with shorter legs. Total length 1.93 (Figure 25A-B). Carapace 0.78 long, 0.75 wide. Opisthosoma 1.18 long, 1.00 wide. Clypeus 0.15 high. Leg measurements: Ⅰ 6.02 (1.68, 0.28, 1.73, 1.30, 1.03); Ⅱ 5.29 (1.45, 0.28, 1.43, 1.25, 0.88); Ⅲ 4.34 (1.25, 0.25, 1.13, 1.03, 0.68); Ⅳ 5.67 (1.63, 0.28, 1.58, 1.28, 0.90). Vulva (Figure 25C): spermathecae coiled, atrium fusiform, anterior margin with pointed process medially.

**Distribution.** China (Guizhou).

### *Leptonetela gang* Wang & Li sp. nov. Figures 26-27, 97

**Figure 26:**
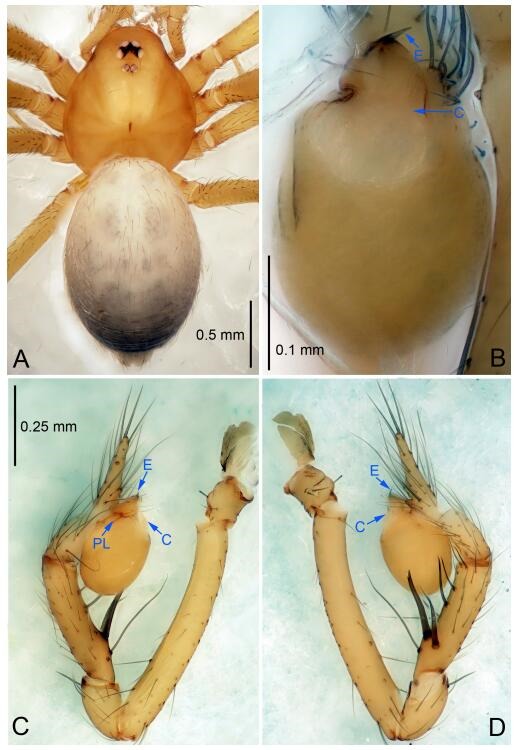
*Leptonetela gang* sp. nov., holotype male

**Figure 27:**
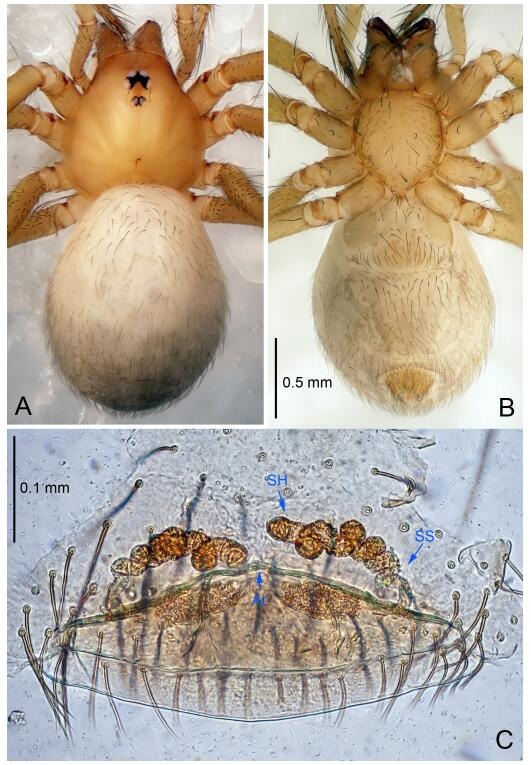
*Leptonetela gang* sp. nov., one of the paratype females

**Type material. Holotype:** male (IZCAS), Gang Cave, N26.87°, E108.91°, Tunhou, Nanming Town, Jianhe County, Kaili City, Guizhou Province, China, 15 December 2011, Z. Zha leg. **Paratypes:** 15 males and 6 females, same data as holotype; 4 males and 5 females, Long Cave, N26.85°, E108.79°, Longtang, Liangshang Town, Sansui County, Kaili City, Guizhou Province, China, 18 December 2011, Z. Zha leg; 5 males and 5 females, Shenxian Cave, N26.87°, E108.89°, Shixing, Xiaolan Country, Nanming Town, Jianhe County, Kaili City, Guizhou Province, China, 16 December 2011, Z. Zha leg; 5 females, Niu Cave, N26.86°, E108.93°, Cenge, Nanming Town, Jianhe County, Kaili City, Guizhou Province, China, 14 December 2011, Z. Zha leg.

**Etymology.** The specific name refers to the type locality; noun.

**Diagnosis.** This new species is similar to *L. jiulong* Lin & Li, 2010, but can be distinguished by the male pedipalpal tibia with 6 spines retrolaterally, tibia Ⅱ spine thickest, Ⅰ, Ⅱ spines equally length, tibia Ⅱ spine asymmetrically bifurcated (Figure 26D), median apophysis absent, conductor reduced (Figure 26B) (Ⅰ, Ⅱ spines equally strong, tibia Ⅱ spine longest and bifurcate, tibia Ⅰ spine half the length of Ⅱ, median apophysis broad and smooth, conductor rugose, triangular in *L. jiulong*).

**Description. Male (holotype).** Total length 2.63 (Figure 26A). Carapace 1.25 long, 1.00 wide. Opisthosoma 1.50 long, 1.13 wide. Carapace yellow. Ocular area with a pair of setae, six eyes. Median groove needle-shaped, cervical grooves and radial furrows distinct. Clypeus 0.13 high. Opisthosoma gray, ovoid. Leg measurements: Ⅰ 10.66 (3.00, 0.38, 3.13, 2.55, 1.60); Ⅱ 8.96 (2.50, 0.38, 2.55, 2.13, 1.40); Ⅲ 7.55 (2.25, 0.35, 2.00, 1.75, 1.20); Ⅳ 9.68 (2.75, 0.38, 2.75, 2.35, 1.45). Male pedipalp (Figure 26C-D): tibia with 5 long spines prolaterally, 6 spines retrolaterally, tibia Ⅱ spine thickest asymmetrically bifurcated, Ⅰ Ⅱ spines equally length. Cymbium constricted medially, attached to an earlobe-shaped process. Embolus triangular, median apophysis absent, conductor reduced (Figure 26B).

**Female (one of the paratypes).** Similar to male in color and general features, but smaller and with shorter legs. Total length 2.38 (Figure 27A-B). Carapace 1.00 long, 0.88 wide. Opisthosoma 1.50 long, 1.20 wide. Clypeus 0.13 high. Leg measurements: Ⅰ 9.26 (2.50, 0.38, 2.75, 2.13, 1.50); Ⅱ 7.51 (2.00, 0.38, 2.13, 1.75, 1.25); Ⅲ 6.29 (1.88, 0.38, 1.63, 1.40, 1.00); Ⅳ 8.11 (2.25, 0.38, 2.25, 1.88, 1.35). Vulva (Figure 27C): spermathecae coiled, atrium fusiform.

**Distribution.** China (Guizhou).

### *Leptonetela la* Wang & Li sp. nov. Figure 28-29, 97

**Figure 28:**
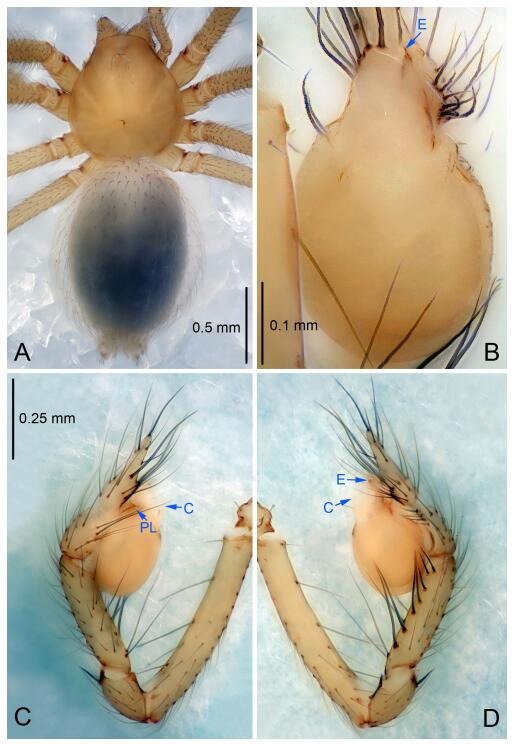
*Leptonetela la* sp. nov., holotype male

**Figure 29:**
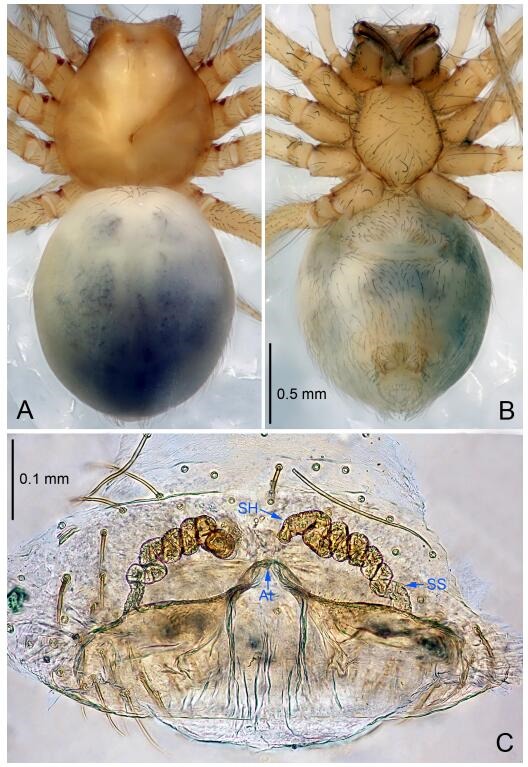
*Leptonetela la* sp. nov., one of the paratype females

**Type material. Holotype:** male (IZCAS), La Cave in Xiaoyakou, N25.80°, E104.95°, Puan County, Qianxinan Prefecture, Guizhou Province, China, 14 July 2012, H. Zhao leg. **Paratypes:** 3 males and 5 females, same data as holotype.

**Etymology.** The specific name refers to the type locality; noun.

**Diagnosis.** This new species is similar to *L. rudong* Wang & Li **sp. nov.** and *L. wenzhu* Wang & Li **sp. nov.** but can be distinguished from *L. wenzhu* Wang & Li **sp. nov.** by the male pedipalpal tibia with 7 spines retrolaterally (tibia with 6 spines retrolaterally in *L. wenzhu* Wang & Li **sp. nov.**); from *L. rudong* Wang & Li **sp. nov.** by the tibia with 4 long setae prolaterally (Figure 28D) (tibia with 2 long setae, 2 spines prolaterally, cymbium with 1 horn-shaped spine on the earlobe-shaped process in *L. rudong* Wang & Li **sp. nov.**); from *L. rudong* Wang & Li **sp. nov.**, and *L. wenzhu* Wang & Li **sp. nov.** by the conductor broad, C shaped (conductor thin, triangular in *L. rudong* Wang & Li **sp. nov.**, bamboo leaf-shaped in *L. wenzhu* Wang & Li **sp. nov.**).

**Description. Male (holotype).** Total length 2.97 (Figure 28A). Carapace 1.25 long, 1.09 wide. Opisthosoma 1.71 long, 1.40 wide. Carapace yellow. Ocular area with a pair of setae, eyes absent. Median groove needle-shaped, cervical grooves and radial furrows distinct. Clypeus 0.17 high. Opisthosoma gray, ovoid. Leg measurements: Ⅰ 10.49 (2.60, 0.40, 3.16, 2.48, 1.85); Ⅱ 9.70 (2.66, 0.41, 2.81, 2.26, 1.56); Ⅲ 8.83 (2.34, 0.40, 2.81, 1.97, 1.31); Ⅳ 10.17 (2.81, 0.41, 2.88, 2.51, 1.56). Male pedipalp (Figure 28C-D): tibia with 4 long setae prolaterally and 7 slender spines retrolaterally, tibia Ⅰ Ⅱ spines of equal length, longer than other spines. Cymbium constricted medially, attaching an earlobe-shaped process. Embolus triangular, prolateral lobe oval. Median apophysis absent. Conductor C shaped in ventral view (Figure 28B).

**Female (one of the paratypes).** Similar to male in color and general features, but smaller and with shorter legs. Total length 2.81 (Figure 29A-B). Carapace 1.09 long, 1.01 wide. Opisthosoma 1.69 long, 1.47 wide. Clypeus 0.17 high. Leg measurements: Ⅰ 9.68 (2.56, 0.40, 2.56, 2.13, 1.62); Ⅱ 8.23 (2.34, 0.34, 2.43, 1.81, 1.31); Ⅲ 7.03 (2.19, 0.34, 2.03, 1.38, 1.09); Ⅳ 9.13 (2.51, 0.34, 2.59, 2.38, 1.31). Vulva (Figure 29C): spermathecae coiled, atrium fusiform, anterior margin of atrium with one large pointed process medially, and covered with short hairs.

**Distribution.** China (Guizhou).

### *Leptonetela rudong* Wang & Li sp. nov. Figures 30-31, 97

**Figure 30:**
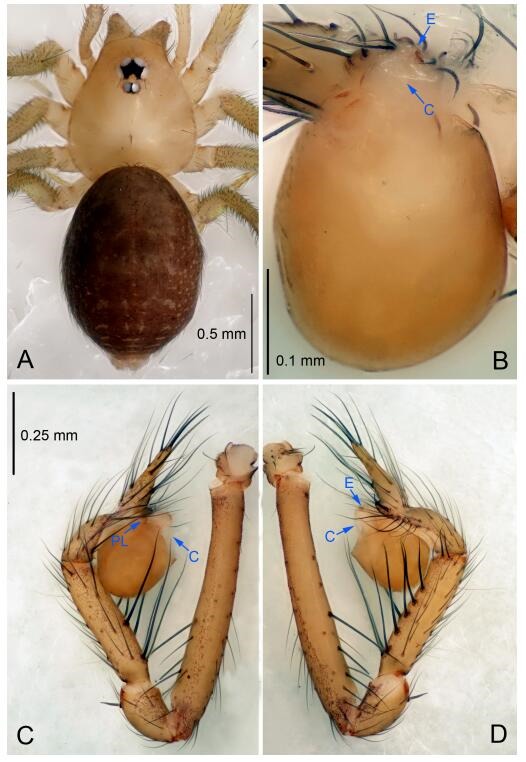
*Leptonetela rudong* sp. nov., holotype male

**Figure 31:**
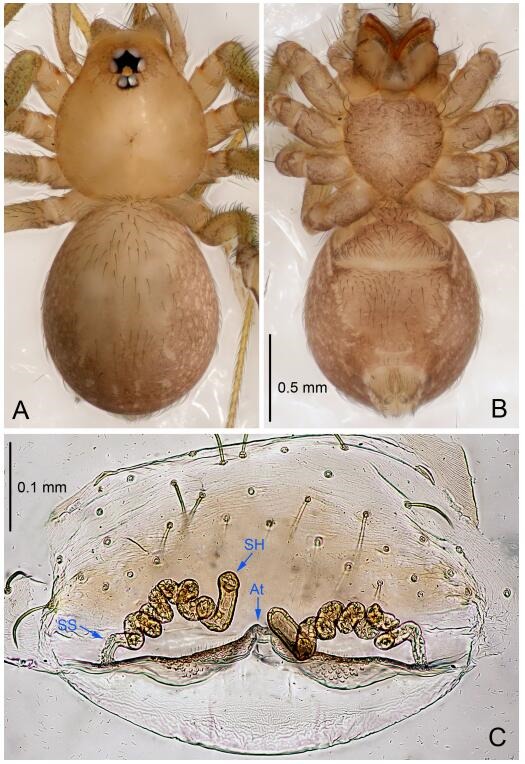
*Leptonetela rudong* sp. nov., one of the paratype females

**Type material. Holotype:** male (IZCAS), Rudong Cave, N25.57°, E110.62°, Longpan Mountain, Dongtian, Xing'an County, Guilin City, Guangxi Zhuang Autonomous Region, China, 11 July 2009, C. Wang & Z. Yao leg. **Paratypes:** 1 male and 3 females, same data as holotype; 3 females, Gouya Cave, N25.46°, E110.11°, Hufeng, Guanyang County, Guilin City, Guangxi Zhuang Autonomous Region, China, 30 August 2009, C. Wang & Z. Yao leg; 2 females, Jiulong Cave, N25.46°, E110.09°, Shifeng, Guanyang County, Guilin City, Guangxi Zhuang Autonomous Region, China, 30 August 2009, C. Wang & Z. Yao leg.

**Etymology.** The specific name refers to the type locality; noun.

**Diagnosis.** This new species is similar to *L. la* Wang & Li **sp. nov.**, and *L. wenzhu* Wang & Li **sp. nov.** but can be distinguished by the male pedipalpal tibia with 2 long setae, 2 spines prolaterally, 1 long seta, and 6 spines retrolaterally, cymbium with 1 horn-shaped spine on the earlobe-shaped process (Figure 30C-D), conductor thin, triangular in ventral view (Figure 30B) (tibia with 4 long setae prolaterally, 7 slender spines retrolaterally, conductor broad, C shaped in *L. la* Wang & Li **sp. nov.**; tibia with 2 long setae prolaterally, 6 spines retrolaterally, with tibia Ⅰ spine strongest, conductor bamboo leaf-shaped in *L. wenzhu* Wang & Li **sp. nov.**).

**Description. Male (holotype).** Total length 2.12 (Figure 30A). Carapace 0.88 long, 0.85 wide. Opisthosoma 1.25 long, 1.05 wide. Carapace yellowish. Ocular area with a pair of setae, eyes six. Median groove needle-shaped, cervical grooves and radial furrows indistinct. Clypeus 0.13 high. Opisthosoma brown, ovoid. Leg measurements: Ⅰ 10.15 (2.84, 0.38, 3.00, 2.38, 1.55); Ⅱ 7.84 (2.08, 0.38, 2.23, 1.88, 1.27); Ⅲ 6.55 (1.83, 0.35, 1.75, 1.62, 1.00); Ⅳ 8.31 (2.25, 0.38, 2.38, 2.05, 1.25). Male pedipalp (Figure 30C-D): tibia with 2 long setae, 2 spines prolaterally, 1 long seta and 6 slender spines retrolaterally, with Ⅰ spine longest. Cymbium constricted medially, with 1 horn-shaped spine on the earlobe-shaped process. Embolus triangular, pedipalpal bulb oval. Median apophysis absent. Conductor thin, triangular in ventral view (Figure 30B).

**Female (one of the paratypes).** Similar to male in color and general features, but larger and with shorter legs. Total length 2.15 (Figure 31A-B). Carapace 0.90 long, 0.88 wide. Opisthosoma 1.33 long, 1.02 wide. Clypeus 0.13 high. Leg measurements: Ⅰ 8.64 (2.35, 0.33, 2.55, 1.88, 1.53); Ⅱ 6.87 (2.03, 0.33, 1.88, 1.50, 1.13); Ⅲ 6.06 (1.90, 0.33, 1.50, 1.35, 0.98); Ⅳ 7.39 (1.98, 0.33, 2.13, 1.78, 1.17). Vulva (Figure 31C): spermathecae coiled, atrium semicircular, anterior margin of atrium with one pointed process medially, and covered with short hairs.

### *Leptonetela wenzhu* Wang & Li sp. nov. Figures 32-33, 97

**Figure 32:**
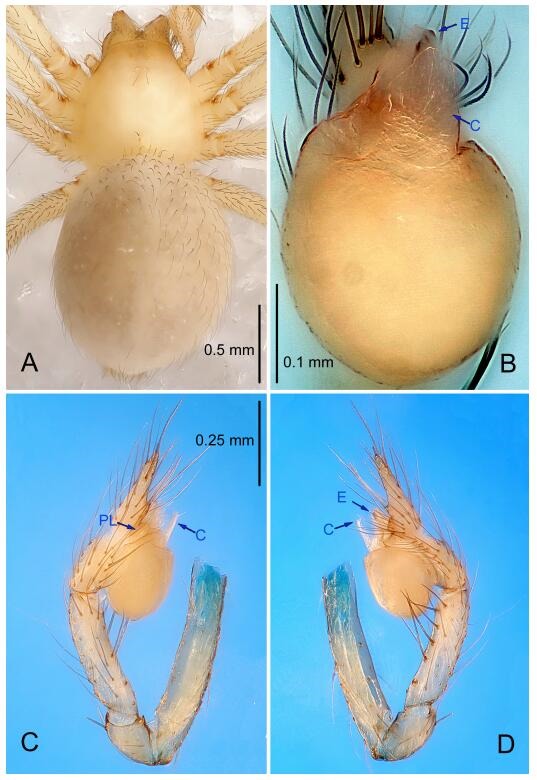
*Leptonetela wenzhu* sp. nov., holotype male

**Figure 33:**
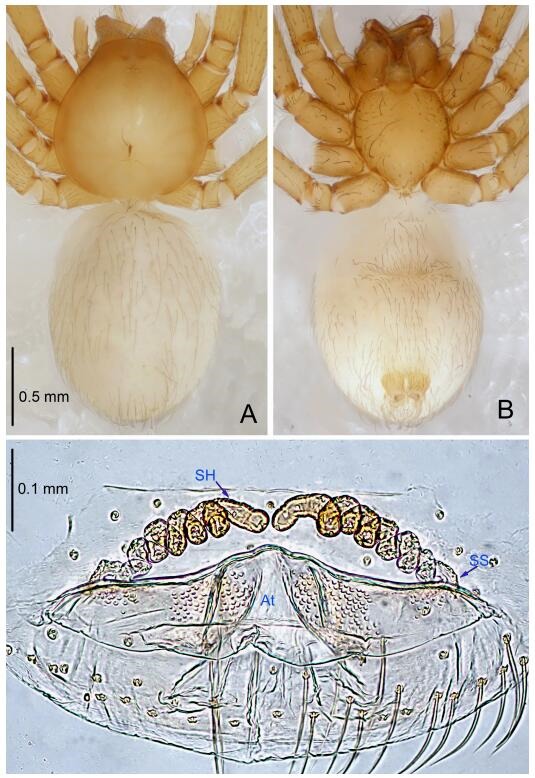
*Leptonetela wenzhu* sp. nov., one of the paratype females

**Type material. Holotype:** male (IZCAS), Wenzhu Cave, N25.44°, E105.13°, Longchang Town, Xingren City, Guizhou Province, China, 16 July 2012, H. Zhao leg. **Paratypes:** 1 male and 2 females, same data as holotype; 4 females, Xiaoya Cave, N25.44°, E105.13°, Yaqiao Town, Xingren City, Guizhou Province, China, 16 July 2012, H. Zhao leg.

**Etymology.** The specific name refers to the type locality; noun.

**Diagnosis.** This new species is similar to *L. rudong* Wang & Li **sp. nov.**, and *L. la* Wang & Li **sp. nov.** but can be distinguished by the male pedipalpal tibia with 6 spines retrolaterally (Figure 32D), conductor bamboo leaf-shaped in ventral view (Figure 32B) (tibia with 1 long seta, 6 spines retrolaterally in *L. rudong* Wang & Li **sp. nov.**, tibia with 7 spines retrolaterally in *L. la* Wang & Li **sp. nov.**, conductor broad, C shaped in *L. la* Wang & Li **sp. nov.**; thin, triangular in *L. rudong* Wang & Li **sp. nov.**).

**Description. Male (holotype).** Total length 2.63 (Figure 32A). Carapace 1.28 long, 1.03 wide. Opisthosoma 1.34 long, 1.09 wide. Carapace yellowish. Ocular area with a pair of setae, PME and PLE absent, ALE reduced to white points. Median groove, cervical groove and radial furrows indistinct. Clypeus 0.25 high. Opisthosoma gray, ovoid. Leg measurements: Ⅰ 10.17 (2.78, 0.37, 3.12, .34, 1.56); Ⅱ 7.34 (2.53, 0.37, 1.94, 1.72, 0.78); Ⅲ 7.70 (2.19, 0.37, 2.03, 1.88, 1.22); Ⅳ 9.28 (2.60, 0.37, 2.56, 2.28, 1.47). Male pedipalp (Figure 32C-D): tibia with 6 spines retrolaterally, arranged equidistantly. Embolus triangular, prolateral lobe absent. Median apophysis absent. Conductor bamboo leaf-shaped in ventral view (Figure 32B).

**Female (one of the paratypes).** Similar to male in color and general features, but larger and with shorter legs. Total length 2.88 (Figure 33A-B). Carapace 1.20 long, 1.00 wide. Opisthosoma 1.60 long, 1.20 wide. Clypeus 0.18 high. Leg measurements: Ⅰ 8.62 (2.53, 0.37, 2.51, 1.90, 1.31); Ⅱ 7.36 (2.09, 0.34, 1.94, 1.65, 1.34); Ⅲ 6.18 (1.55, 0.31, 1.69, 1.47, 1.16); Ⅳ 7.98 (2.44, 0.31, 2.01, 1.88, 1.34). Vulva (Figure 33C): spermathecae coiled, atrium triangular.

**Distribution.** China (Guizhou).

### *Leptonetela longli* Wang & Li sp. nov. Figures 34-35, 97

**Figure 34:**
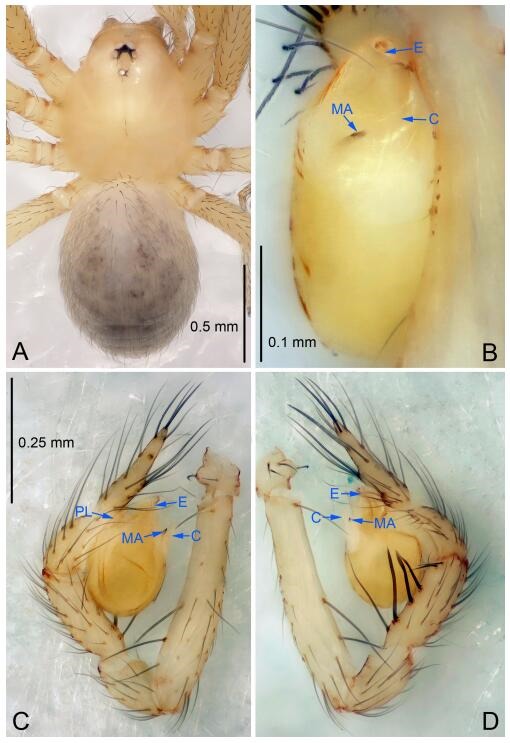
*Leptonetela longli* sp. nov., holotype male

**Figure 35:**
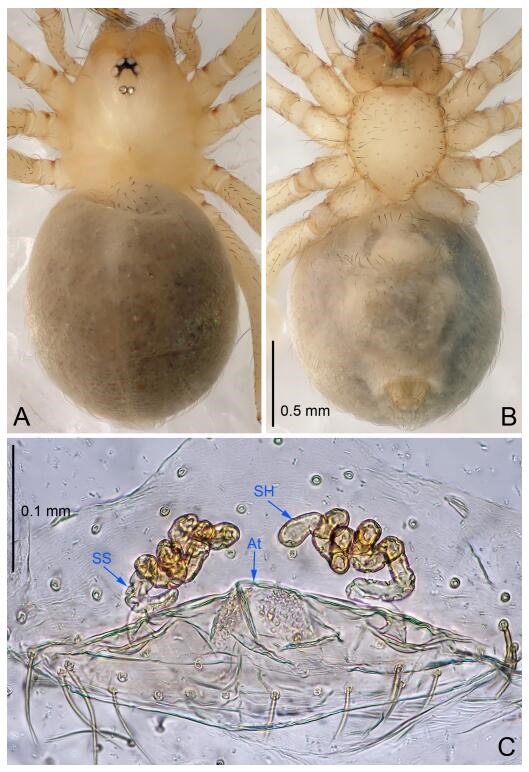
*Leptonetela longli* sp. nov., one of the paratype females

**Type material. Holotype:** male (IZCAS), Underground River, N25.27°, E107.44°, Longli, Liuzhai Town, Nandan County, Hechi City, Guangxi Zhuang Autonomous Region, China, 29 January 2015, Y. Li & Z. Chen leg. **Paratypes:** 3 males and 4 females, same data as holotype.

**Etymology.** The specific name refers to the type locality; noun.

**Diagnosis.** This new species is similar to *L. chiosensis* Wang & Li, 2011 and *L. panbao* Wang & Li **sp. nov.**, but can be distinguished by the male pedipalpal tibia Ⅰ, Ⅱ and Ⅲ spines equally strong (Figure 34D), conductor C shaped (Figure 34B) (tibia Ⅰ spine strong, conductor triangular in *L. chiosensis*; tibia spines slender, cymbium with 1 strong spine on the earlobe-shaped process, conductor reduced, embolus with 1 tooth basally in *L. panbao* Wang & Li **sp. nov.**).

**Description. Male (holotype).** Total length 1.88 (Figure 34A). Carapace 0.87 long, 0.75 wide. Opisthosoma 1.00 long, 0.80 wide. Carapace yellowish. Ocular area with a pair of setae, eyes six. Median groove, cervical grooves and radial furrows indistinct. Clypeus 0.10 high. Opisthosoma gray, ovoid. Leg measurements: Ⅰ 6.38 (1.75, 0.25, 1.88, 1.50, 1.00); Ⅱ 5.03 (1.40, 0.25, 1.38, 1.25, 0.75); Ⅲ 4.25 (1.37, 0.20, 1.13, 1.00, 0.55); Ⅳ 5.71 (1.63, 0.25, 1.60, 1.35, 0.88). Male pedipalp (Figure 34C-D): tibia with 3 long spines prolaterally, 5 spines retrolaterally, tibia Ⅰ spine longest, Ⅰ, Ⅱ Ⅲ spines equally strong, stronger than other spines. Embolus triangular, prolateral lobe reduced. Median apophysis"丿"shaped in ventral view. Conductor "C" shaped in ventral view (Figure 34B).

**Female (one of the paratypes).** Similar to male in color and general features, but larger and with shorter legs. Total length 1.95 (Figure 35A-B). Carapace 0.88 long, 0.88 wide. Opisthosoma 1.25 long, 1.00 wide. Clypeus 0.10 high. Leg measurements: Ⅰ 5.03 (1.38, 0.25, 1.50, 1.15, 0.75); Ⅱ 4.58 (1.25, 0.25, 1.13, 1.10, 0.85); Ⅲ 3.55 (1.00, 0.20, 0.88, 0.87, 0.60); Ⅳ 4.76 (1.30, 0.25, 1.38, 1.13, 0.70). Vulva (Figure 35C): spermathecae coiled, atrium fusiform.

**Distribution.** China (Guangxi).

### *Leptonetela panbao* Wang & Li sp. nov. Figures 36-37, 97

**Figure 36:**
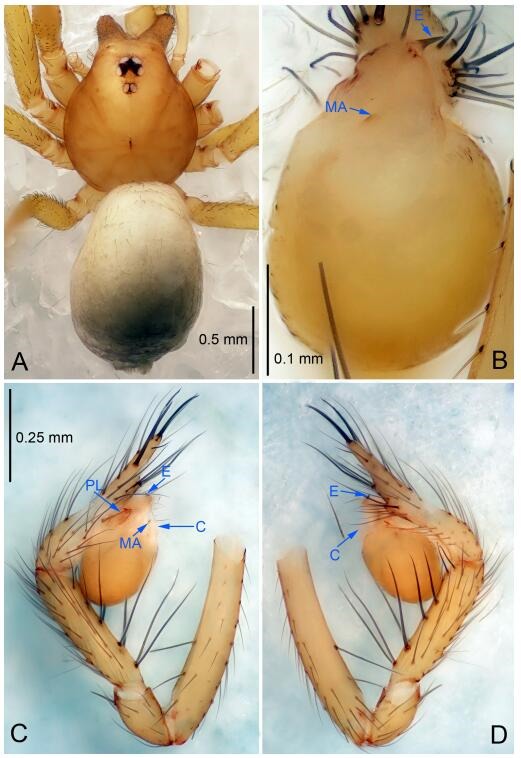
*Leptonetela panbao* sp. nov., holotype male

**Figure 37:**
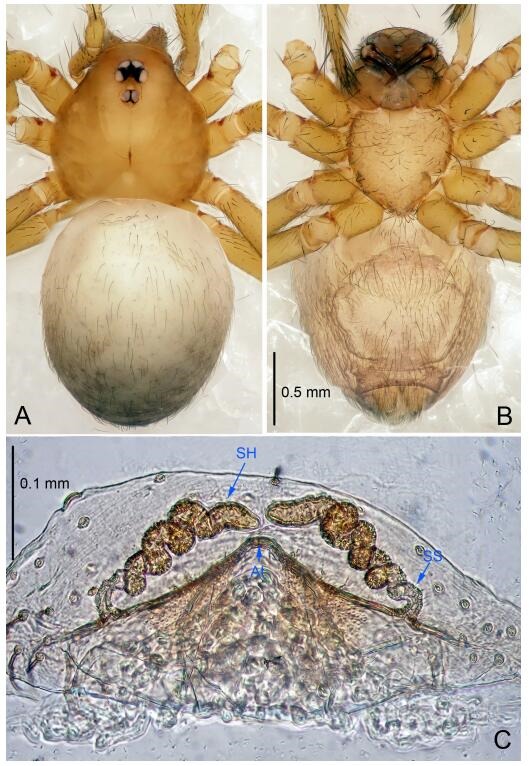
*Leptonetela panbao* sp. nov., one of the paratype females

**Type material. Holotype:** male (IZCAS), Panbao Cave, N28.38°, E108.67°, Panbao, Shichang Town, Songtao County, Tongren City, Guizhou Province, China, 8 March 2013, H. Zhao & J. Liu leg. **Paratypes:** 2 male and 4 females, same data as holotype.

**Etymology.** The specific name refers to the type locality; noun.

**Diagnosis.** This new species is similar to *L. chiosensis* Wang & Li, 2011 and *L. Longli* Wang & Li **sp. nov.**, but can be distinguished by the male pedipalpal tibial spines slender, equally strong, cymbium with 1 strong spine on the earlobe-shaped process (Figure 36D), conductor reduced, embolus with 1 tooth basally (Figure 36B) (tibia Ⅰ spine stronger than other spines, conductor triangular in *L. chiosensis*; tibia Ⅰ, Ⅱ and Ⅲ spines equally strong, stronger than other spines, conductor C shaped in *L. panbao* Wang & Li **sp. nov.**).

**Description. Male (holotype).** Total length 2.38 (Figure 36A). Carapace 1.15 long, 1.00 wide. Opisthosoma 1.25 long, 0.90 wide. Carapace yellow. Six eyes. Median groove needle-shaped, cervical grooves and radial furrows distinct. Clypeus 0.13 high. Opisthosoma gray, ovoid. Leg measurements: Ⅰ 10.50 (2.75, 0.35, 3.50, 2.40, 1.50); Ⅱ 7.86 (2.13, 0.35, 2.35, 1.90, 1.13); Ⅲ 6.22 (1.75, 0.34, 1.75, 1.38, 1.00); Ⅳ 8.43 (2.38, 0.35, 2.40, 2.05, 1.25). Male pedipalp (Figure 36C-D): tibia with 4 long spines prolaterally, 5 slender spines retrolaterally, the spines equally strong, tibia Ⅰ spine longest. Cymbium not wrinkled, earlobe-shaped process small, with 1 spine. Embolus triangular, bearing a basal tooth, prolateral lobe oval. Median apophysis single quote shaped, " ′ " in ventral view. Conductor reduced (Figure 36B).

**Female (one of the paratypes).** Similar to male in color and general features, but larger and with shorter legs. Total length 2.50 (Figure 37A-B). Carapace 1.13 long, 1.00 wide. Opisthosoma 1.62 long, 1.25 wide. Clypeus 0.13 high. Leg measurements: Ⅰ 8.85 (2.30, 0.30, 2.62, 2.13, 1.50); Ⅱ 6.81 (1.88, 0.30, 2.00, 1.50, 1.13); Ⅲ 5.76 (1.63, 0.25, 1.63, 1.25, 1.00); Ⅳ 7.58 (2.25, 0.30, 2.13, 1.75, 1.15). Vulva (Figure 37C): spermathecae coiled, atrium triangular, anterior margin of atrium covered with short hairs.

**Distribution.** China (Guizhou).

### *Leptonetela feilong* Wang & Li sp. nov. Figures 38-39, 97

**Figure 38:**
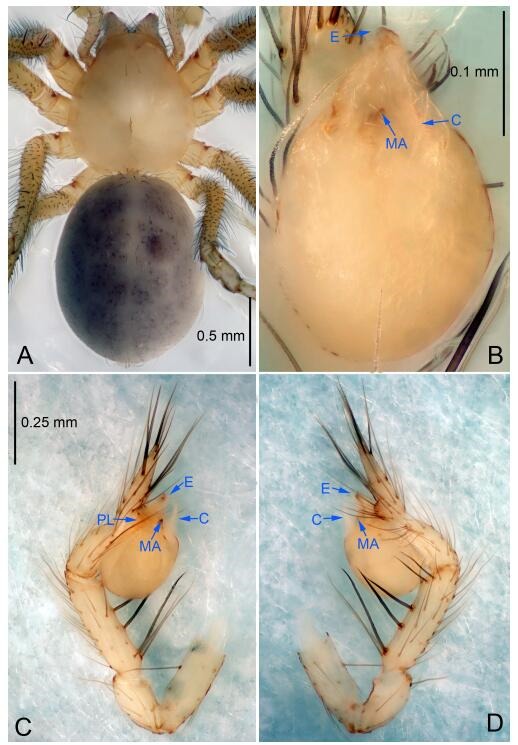
*Leptonetela feilong* sp. nov., holotype male

**Figure 39:**
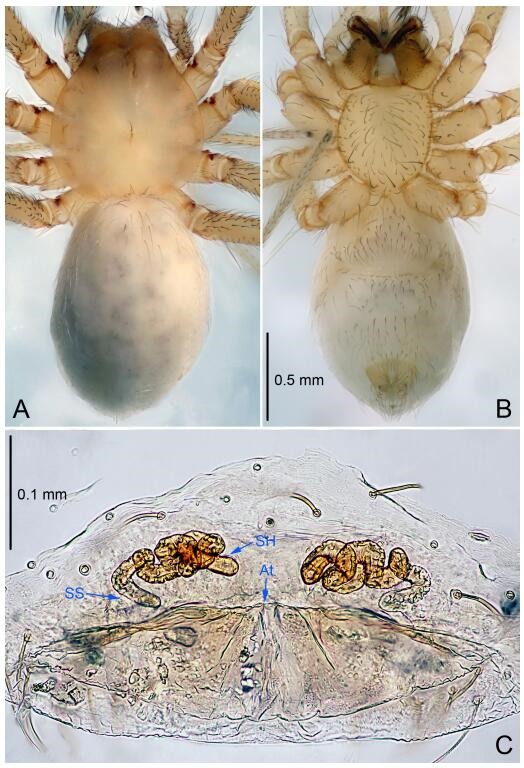
*Leptonetela feilong* sp. nov., one of the paratype females

**Type material. Holotype:** male (IZCAS), Feilong Cave, N26.44°, E107.02°, Longli Town, Qiannan Prefecture, Guizhou Province, China, 27 July 2012, H. Zhao leg. **Paratypes:** 9 females, same data as holotype; 1 female, Lianhua Cave, N26.43°, E106.95°, Lianhua Town, Qiannan Prefecture, Guizhou Province, China, 27 July 2012, H. Zhao leg.

**Etymology.** The specific name refers to the type locality; noun.

**Diagnosis.** This new species is similar to *L. yangi* Lin & Li, 2010 and *L. jiahe* Wang & Li **sp. nov.**, but can be distinguished from *L. yangi* by the male pedipalpal cymbium constricted medially, attaching an earlobe-shaped process (Figure 38D), conductor triangular (Figure 38B) (cymbium not constricted, earlobe-shaped process absent, conductor reduced in *L. yangi*), from *L. jiahe* Wang & Li **sp. nov.** by the median apophysis "m"-shaped, conductor triangular (cymbium with 1 spine on the earlobe-shaped process, and 1 curved long spine medially, median apophysis like pointedprocess, with 3 sclerotized spots distally, conductor C shaped in *L. jiahe* Wang & Li **sp. nov.**).

**Description. Male (holotype).** Total length 2.31 (Figure 38A). Carapace 1.02 long, 1.30 wide. Opisthosoma 1.30 long, 1.14 wide. Carapace yellowish. Ocular area with a pair of setae, eyes absent. Median groove needle-shaped, cervical grooves and radial furrows indistinct. Clypeus 0.15 high. Opisthosoma gray, ovoid. Leg measurements: Ⅰ 9.53 (2.66, 0.40, 2.81, 2.19, 1.47); Ⅱ 8.09 (2.41, 0.40, 2.41, 1.93, 0.94); Ⅲ 7.17 (2.03, 0.40, 1.94, 1.72, 1.08); Ⅳ 8.82 (2.71, 0.41, 2.51, 1.94, 1.25). Male pedipalp (Figure 38C-D): tibia with 2 long setae prolaterally, 5 spines retrolaterally, spines Ⅰ, Ⅱ and Ⅲ equally strong, spines Ⅰ longest. Cymbium constricted medially, attaching an earlobe-shaped process retrolaterally. Embolus triangular, prolateral lobe nearly absent. Median apophysis "m"-shaped. Conductor triangular (Figure 8B).

**Female (one of the paratypes).** Similar to male in color and general features, but smaller and with shorter legs. Total length 2.13 (Figure 39A-B). Carapace 0.88 long, 0.88 wide. Opisthosoma 1.25 long, 0.87 wide. Clypeus 0.08 high. Leg measurements: Ⅰ 8.44 (2.38, 0.30, 2.51, 1.87, 1.38); Ⅱ 7.48 (2.05, 0.31, 2.25, 1.62, 1.25); Ⅲ 6.20 (1.75, 0.31, 1.75, 1.38, 1.01); Ⅳ 7.73 (2.25, 0.32, 2.25, 1.78, 1.13). Vulva (Figure 39C): spermathecae coiled, atrium fusiform.

**Distribution.** China (Guizhou).

### *Leptonetela jiahe* Wang & Li sp. nov Figures 40-41, 97

**Figure 40:**
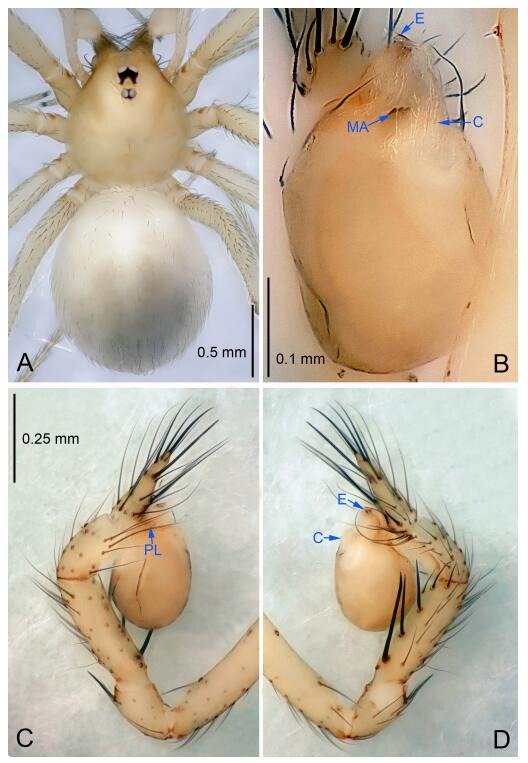
*Leptonetela jiahe* sp. nov., holotype male

**Figure 41:**
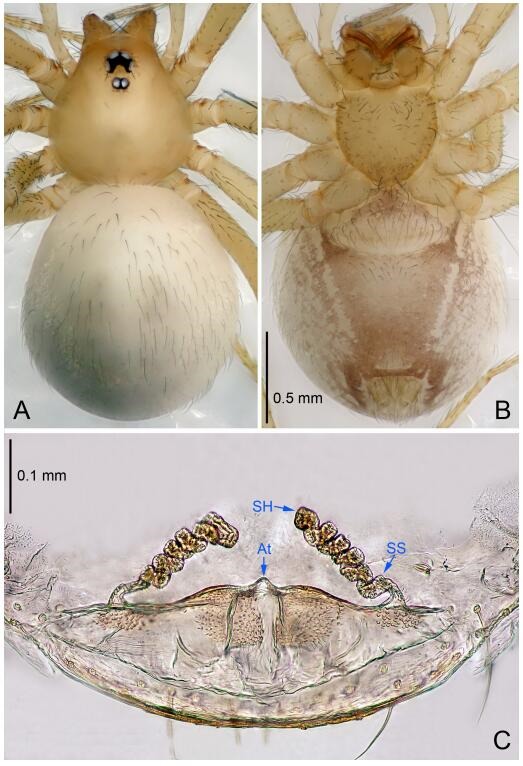
*Leptonetela jiahe* sp. nov., one of the paratype females

**Type material. Holotype:** male (IZCAS), Jiahe Cave, N25.25°, E110.20°, Lingui Town, Lingui County, Guilin City, Guangxi Zhuang Autonomous Region, China, 20 December 2013, H. Zhao leg. **Paratypes:** 3 males and 5 females, same data as holotype; 6 males and 5 females, Flytiger Cave, N25.25°, E110.20°, Lingui Town, Lingui County, Guilin City, Guangxi Zhuang Autonomous Region, China, 20 December 2013, H. Zhao leg.

**Etymology.** The specific name refers to the type locality; noun.

**Diagnosis.** This new species is similar to *L. yangi* Lin & Li, 2010, and *L. feilong* Wang & Li **sp. nov.**, but can be distinguished by the male pedipalpal cymbium with 1 short spine on the earlobe-shaped process, and 1 curved, long spine retrolaterally (Figure 40D), median apophysis shaped like a pointed process, tip with 3 sclerotized spots, conductor C shaped (Figure 40B) (median apophysis "m"-shaped in *L. yangi* and *L. feilong* Wang & Li **sp. nov.**, conductor reduced in *L. yangi*; triangular in *L. feilong* Wang & Li **sp. nov.**).

**Description. Male (holotype).** Total length 2.43 (Figure 40A). Carapace 1.03 long, 0.90 wide. Opisthosoma 1.43 long, 1.22 wide. Carapace yellowish. Ocular area with a pair of setae, six eyes. Median groove, cervical grooves and radial furrows indistinct. Clypeus 0.13 high. Opisthosoma white, ovoid. Leg measurements: Ⅰ 9.22 (2.48, 0.38, 2.66, 2.20, 1.50); Ⅱ 7.58 (2.13, 0.35, 2.20, 1.75, 1.15); Ⅲ 6.21 (1.75, 0.30, 1.63, 1.53, 1.00); Ⅳ 8.11 (2.25, 0.35, 2.13, 2.00, 1.38). Male pedipalp (**Figure** 40C-D): tibia with 5 slender spines retrolaterally, spines Ⅰ, Ⅱ and Ⅲ equally strong, stronger than other spines, spines Ⅰ longest. Cymbium constricted medially, retrolaterally attaching to 1 curved spine and an earlobe-shaped process, with 1 short spine. Embolus triangular, and prolateral lobe absent. Median apophysis shaped like pointed process, with 3 sclerotized spots distally. Conductor C shaped (Figure 40B).

**Female (one of the paratypes).** Similar to male in color and general features, but smaller and with shorter legs. Total length 2.30 (Figure 41A-B). Carapace 0.93 long, 0.80 wide. Opisthosoma 1.45 long, 1.28 wide. Clypeus 0.13 high. Leg measurements: Ⅰ -(1.95, 0.38, -, -, -); Ⅱ 5.91 (1.63, 0.35, 1.70, 1.25, 0.98); Ⅲ 4.96 (1.38, 0.33, 1.30, 1.12, 0.83); Ⅳ 6.53 (1.85, 0.35, 1.78, 1.50, 1.05). Vulva (Figure 41C): spermathecae coiled, atrium fusiform.

**Distribution.** China (Guangxi).

### *Leptonetela xianren* Wang & Li sp. nov. Figures 42-43, 97

**Figure 42:**
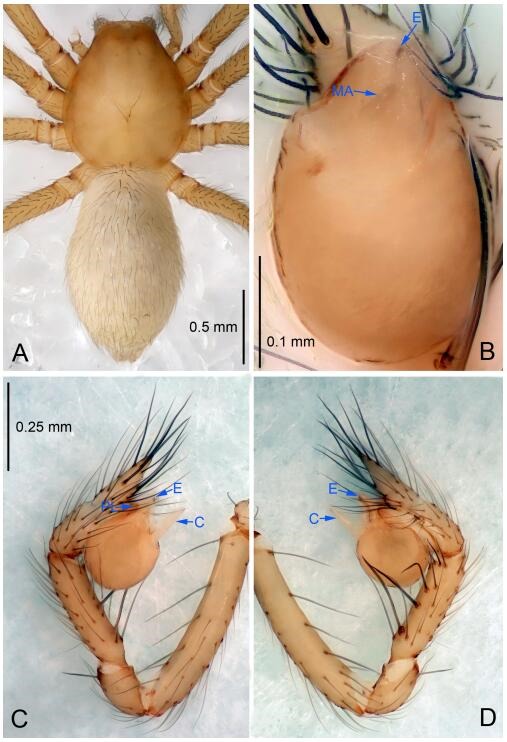
*Leptonetela xianren* sp. nov., holotype male

**Figure 43:**
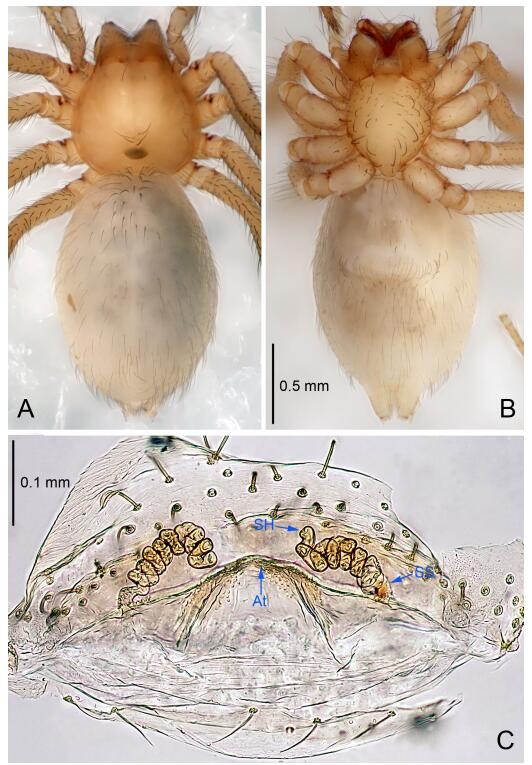
*Leptonetela xianren* sp. nov., one of the paratype females

**Type material. Holotype:** male (IZCAS), Xianren Cave, N29.73°, E110.31°, Yvpingxini, Zouma Town, Hefeng County, Enshi Tujia and Miao Autonomous Prefecture, Hubei Province, China, 27 January 2011, Y. Li & J. Liu leg. **Paratypes:** 2 males and 3 females, same data as holotype.

**Etymology.** The specific name refers to the type locality; noun.

**Diagnosis.** This new species is similar to *L. liping* Lin & Li, 2010, and *L. parlonga* Wang & Li, 2011 but can be distinguished by the male pedipalpal tibia with 5 slender spines retrolaterally, with spines Ⅰ longest (Figure 42D) (tibia with 5 spines retrolaterally, spines Ⅰ strong and longest in *L. liping*, 6 slender spines in *L. parlonga*); median apophysis triangular (Figure 42B) (median apophysis like pointed process in *L. liping*; ligulate in *L. parlonga*); from *L. parlonga* by the cymbium retrolaterally with 1 horn-shaped spine on the earlobe-shaped process in *L. parlonga*.

**Description. Male (holotype).** Total length 2.23 (Figure 42A). Carapace 0.95 long, 0.93 wide. Opisthosoma 1.25 long, 0.88 wide. Carapace yellow. Ocular zone with a pair of setae, eyes absent. Median groove, cervical groove and radial furrows indistinct. Clypeus 0.13 high. Opisthosoma gray, ovoid, lacking distinct pattern. Leg measurements: Ⅰ 8.99 (2.50, 0.38, 2.48, 2.00, 1.63); Ⅱ 8.48 (2.38, 0.37, 2.28, 1.90, 1.55); Ⅲ 7.12 (2.03, 0.33, 1.88, 1.63, 1.25); Ⅳ 7.88 (2.48, 0.38, 2.07, 1.60, 1.35). Leg formula: Ⅰ-Ⅱ-Ⅳ-Ⅲ. Male pedipalp (Figure 42C-D): femur with 5 spines ventrally, tibia with 3 long setae prolaterally, 2 long setae and 5 slender spines retrolaterally, with spines Ⅰ longest. Cymbium constricted medially, attaching an earlobe-shaped process retrolaterally. Embolus triangular, prolateral lobe indistinct. Median apophysis triangular. Conductor bamboo leaf-shaped in ventral view (Figure 42B).

**Female (one of the paratypes).** Similar to male in color and general features but larger and with shorter legs. Total length 2.38 (Figure 43A-B). Carapace 0.85 long, 0.83 wide. Opisthosoma 1.55 long, 1.03 wide. Clypeus 0.15 high. Leg measurements: Ⅰ 8.11 (2.25, 0.38, 2.20, 1.88, 1.40); Ⅱ 7.71 (2.18, 0.35, 2.13, 1.70, 1.35); Ⅲ 6.76 (2.00, 0.35, 1.78, 1.50, 1.13); Ⅳ 7.79 (2.45, 0.40, 2.03, 1.58, 1.33). Vulva (Figure 43C): spermathecae coiled, atrium fusiformed.

**Distribution.** China (Hubei).

### *Leptonetela tiankeng* Wang & Li sp. nov. Figures 44-45, 97

**Figure 44:**
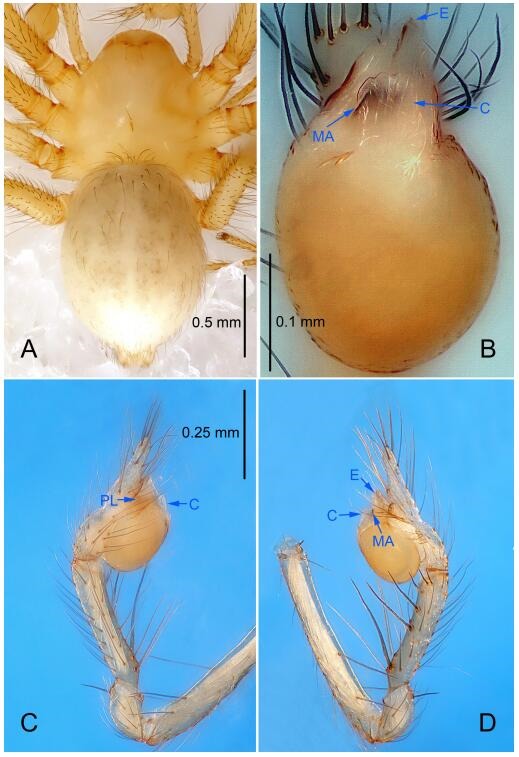
*Leptonetela tiankeng* sp. nov., holotype male

**Figure 45:**
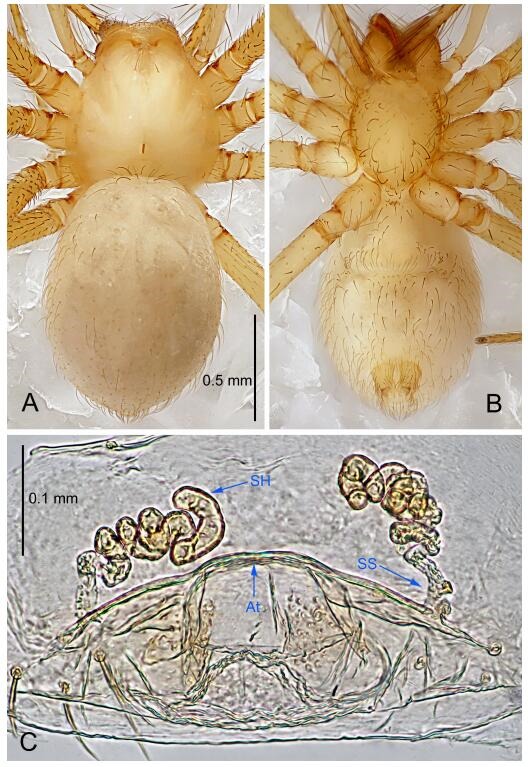
*Leptonetela tiankeng* sp. nov., one of the paratype females

**Type material. Holotype:** male (IZCAS), Tiankeng Cave, N26.64°, E104.80°, Hegou, Dewu Town, Zhongshan County, Liupanshui City, Guizhou Province, China, 9 November 2011, H. Chen & Z. Zha leg. **Paratypes:** 4 males and 5 females, same data as holotype; 2 females, Luoshui Cave, N26.64°, E104.80°, Hegou, Dewu Town, Zhongshan County, Liupanshui City, Guizhou Province, China, 9 November 2011, H. Chen & Z. Zha leg.

**Etymology.** The specific name refers to the type locality; noun.

**Diagnosis.** This new species is similar to *L. rudicula* Wang & Li, 2011, but can be distinguished by the male pedipalpal tibia with 6 spines retrolaterally (Figure 44D), prolateral lobe indistinct (Figure 44C), conductor broad and long, distal edge undulate (Figure 44B) (5 spines retrolaterally, prolateral lobe oval, conductor short, C shaped in *L. rudicula*).

**Description. Male (holotype).** Total length 2.03 (Figure 44A). Carapace 1.00 long, 0.85 wide. Opisthosoma 1.10 long, 0.88 wide. Carapace yellow. Ocular area with a pair of setae, eyes absent. Median groove needle-shaped, cervical grooves and radial furrows indistinct. Clypeus 0.13 high. Opisthosoma yellowish, ovoid. Leg measurements: Ⅰ 9.30 (2.48, 0.35, 2.81, 2.23, 1.43); Ⅱ 8.46 (2.35, 0.35, 2.30, 2.18, 1.28); Ⅲ 7.11 (2.05, 0.30, 1.98, 1.75, 1.03); Ⅳ 8.51 (2.43, 0.35, 2.33, 2.15, 1.25). Male pedipalp (Figure 44C-D): tibia with 5 long setae prolaterally, 6 slender spines retrolaterally, with spines Ⅰ longest. Cymbium slightly constricted medially, attached to an earlobe-shaped process retrolaterally. Embolus triangular, prolateral lobe indistinct. Median apophysis flake-like, sclerotized distally. Conductor broad, distal edge undulate (Figure 44B).

**Female (one of the paratypes).** Similar to male in color and general features, but smaller and with shorter legs. Total length 1.93 (Figure 45A-B). Carapace 0.83 long, 0.73 wide. Opisthosoma 1.13 long, 0.98 wide. Clypeus 0.13 high. Leg measurements: Ⅰ 7.68 (2.23, 0.34, 2.23, 1.63, 1.25); Ⅱ 6.41 (1.90, 0.35, 1.78, 1.38, 1.00); Ⅲ 5.69 (1.68, 0.28, 1.58, 1.38, 0.77); Ⅳ 7.19 (2.00, 0.33, 2.03, 1.70, 1.13). Vulva (Figure 45C): spermathecae coiled, atrium triangular.

**Distribution.** China (Guizhou).

### *Leptonetela mayang* Wang & Li sp. nov. Figures 46-47, 97

**Figure 46:**
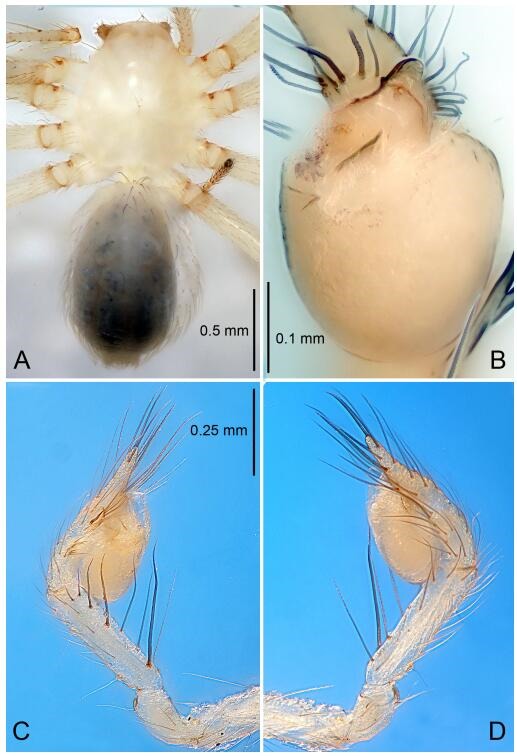
*Leptonetela mayang* sp. nov., holotype male

**Figure 47:**
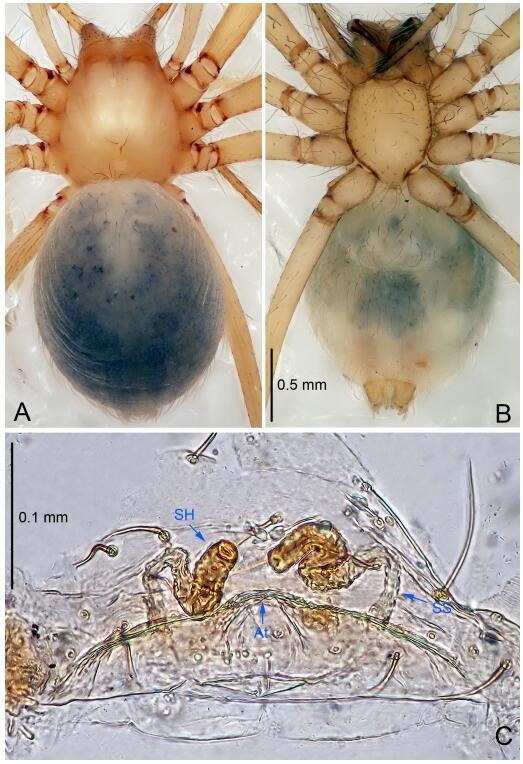
*Leptonetela mayang* sp. nov., paratype female

**Type material. Holotype:** male (IZCAS), Mayang Cave, N28.55°, E108.06°, Quankou, Dejiang County, Tongren City, Guizhou Province, China, 10 August 2012, H. Zhao leg. **Paratype:** 1 female, same data as holotype.

**Etymology.** The specific name refers to the type locality; noun.

**Diagnosis.** This new species can be distinguished from all other species of the genus by the male pedipalpal cymbium with one curved, short spine medially in retrolateral view, median apophysis triangular, spermathecae not tightly twisted, just spiraled in the female.

**Description. Male (holotype).** Total length 2.13 (Figure 46A). Carapace 1.10 long, 0.75 wide. Opisthosoma 0.90 long, 1.00 wide. Carapace white. Eye absent. Median groove, cervical grooves and radial furrows indistinct. Clypeus 0.13 high. Opisthosoma white, ovoid. Leg measurements: Ⅰ 8.98 (2.50, 0.30, 2.63, 2.05, 1.50); Ⅱ 7.68 (2.13, 0.30, 2.25, 1.75, 1.25); Ⅲ 6.76 (2.00, 0.25, 1.88, 1.63, 1.00); Ⅳ 8.21 (2.25, 0.30, 2.38, 1.88, 1.40). Male pedipalp (Figure 46C-D): tibia with 3 long setae prolaterally, and 5 slender spines retrolaterally, spines Ⅰ longest. Cymbium not wrinkled, earlobe-shaped process indistinct, and with 1 curved, short spine retrolaterally. Bulb with spoon-shaped embolus, prolateral lobe indistinct. Median apophysis triangular in ventral view. Conductor thin, triangular in ventral view (Figure 46B).

**Female.** Similar to male in color and general features, but larger and with shorter legs. Total length 2.37 (Figure 47A-B). Carapace 0.88 long, 0.88 wide. Opisthosoma 1.65 long, 1.00 wide. Clypeus 0.13 high. Leg measurements: Ⅰ -(2.25, 0.30, -, -, -); Ⅱ 6.81 (2.00, 0.30, 1.88, 1.50, 1.13); Ⅲ 6.16 (1.88, 0.25, 1.75, 1.38, 0.90); Ⅳ 7.28 (2.13, 0.30, 2.00, 1.60, 1.25). Vulva (Figure 47C): spermathecae spiraled, atrium triangular.

**Distribution.** China (Guizhou).

### *Leptonetela gubin* Wang & Li sp. nov. Figures 48-49, 97

**Figure 48:**
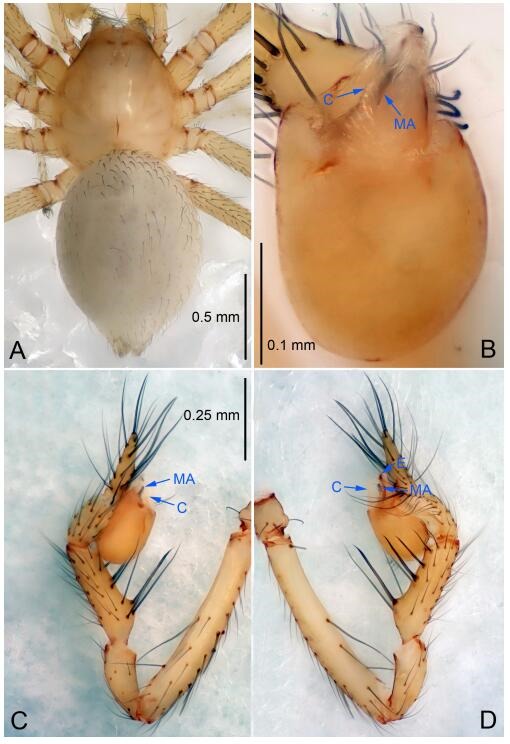
*Leptonetela gubin* sp. nov., holotype male

**Figure 49:**
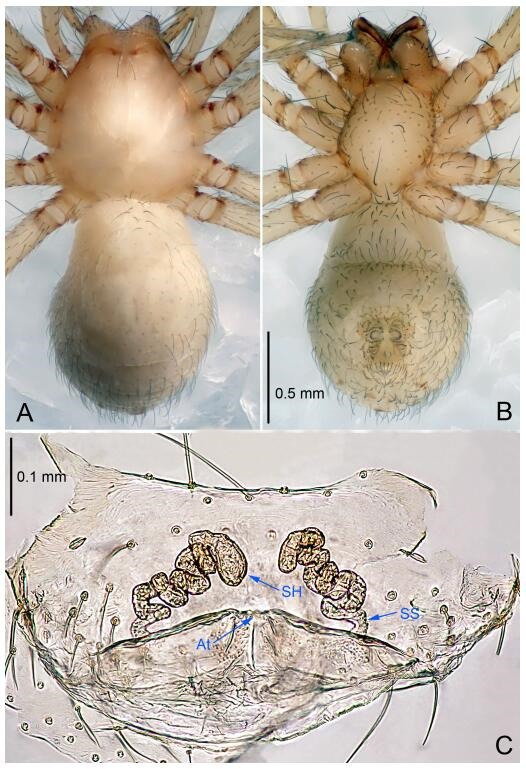
*Leptonetela gubin* sp. nov., one of the paratype females

**Type material. Holotype:** male (IZCAS), Gubin River, N26.50°, E107.52°, Gubin, Xingshan Town, Majiang County, Shengkaili City, Guizhou Province, China, 28 November 2011, H. Chen & Z. Zha leg. **Paratypes:** 22 males and 14 females, same data as holotype; 4 males and 5 females, nameless Cave, N26.50°, E107.52°, Gubin, Xingshan Town, Majiang County, Shengkaili City, Guizhou Province, China, 28 November 2011, H. Chen & Z. Zha leg.

**Etymology.** The specific name refers to the type locality; noun.

**Diagnosis.** This new species is similar to *L. jinsha* Lin & Li, 2010, *L. quinquespinata* (Chen & Zhu, 2008), *L. xinhua* Wang & Li **sp. nov.**, *L. lujia* Wang & Li **sp. nov.** and *L. xinhua* Wang & Li **sp. nov.** but can be distinguished by the male pedipalpal tibia with 4 slender spines prolaterally, 5 slender spines retrolaterally, with spines Ⅰ, Ⅱ equal length, cymbium with 2 long curved spines on earlobe-shaped process retrolaterally (Figure 48D) (tibia with 3 long setae prolaterally, 1 long setae and 5 spines retrolaterally, with spines Ⅰ strongest, tip asymmetrically bifurcated in *L. jinsha*; tibia with 3 long setae prolaterally, 6 large spines retrolaterally, with spines Ⅰ longest in *L. quinquespinata*; tibia with 4 long setae prolaterally, 5 slender spines retrolaterally, with spines Ⅰ longest, spines Ⅱ Ⅲ equal length in *L. lujia* Wang & Li **sp. nov.**; embolus bifurcated, tibia with 5 slender spines prolaterally, 5 slender spines retrolaterally, conductor triangular in *L. xinhua* Wang & Li **sp. nov.**); from *L. jinsha*, *L. lujia* Wang & Li **sp. nov.** and *L. xinhua* Wang & Li **sp. nov.** by the semicircular conductor, base of median apophysis distinctly swollen, 4 times wider than the tip (Figure 48B) (conductor broad, tip undulate in *L. jinsha*; conductor thin, triangular in *L. lujia* Wang & Li **sp. nov.** and *L. xinhua* Wang & Li **sp. nov.**; base of median apophysis slightly swollen in *L. jinsha*, *L. lujia* Wang & Li **sp. nov.**).

**Description. Male (holotype).** Total length 1.88 (Figure 48A). Carapace 0.80 long, 0.78 wide. Opisthosoma 1.13 long, 0.93 wide. Carapace yellowish. Ocular area with a pair of setae, eyes absent. Median groove needle-shaped, cervical grooves and radial furrows distinct. Clypeus 0.13 high. Opisthosoma gray, ovoid, lacking distinctive pattern. Leg measurements: Ⅰ 7.51 (2.00, 0.38, 2.13, 1.75, 1.25); Ⅱ 6.48 (1.75, 0.35, 1.80, 1.43, 1.15); Ⅲ 5.51 (1.63, 0.35, 1.40, 1.25, 0.88); Ⅳ 6.82 (1.88, 0.35, 1.88, 1.58, 1.13). Male pedipalp (Figure 48C-D): tibia with 4 long spines prolaterally and 5 spines retrolaterally, with spines Ⅰ, Ⅱ equally length. Cymbium constricted medially, earlobe-shaped process with 2 long curved spines retrolaterally. Embolus triangular, prolateral lobe absent. Median apophysis finger-shaped, base distinctly swollen. Conductor smooth, semicircular in ventral view (Figure 48B).

**Female (one of the paratypes).** Similar to male in color and general features, but larger and with longer legs. Total length 2.30 (Figure 49A-B). Carapace 0.88 long, 0.85 wide. Opisthosoma 1.40 long, 0.95 wide. Clypeus 0.15 high. Leg measurements: Ⅰ 7.69 (2.13, 0.38, 2.25, 1.65, 1.28); Ⅱ 6.71 (1.90, 0.38, 1.95, 1.40, 1.08); Ⅲ 5.85 (1.75, 0.35, 1.50, 1.35, 0.90); Ⅳ 7.02 (2.00, 0.38, 1.93, 1.58, 1.13). Vulva (Figure 49C): spermathecae coiled, atrium triangular.

**Distribution.** China (Guizhou).

### *Leptonetela lujia* Wang & Li sp. nov. Figures 50-51, 97

**Figure 50:**
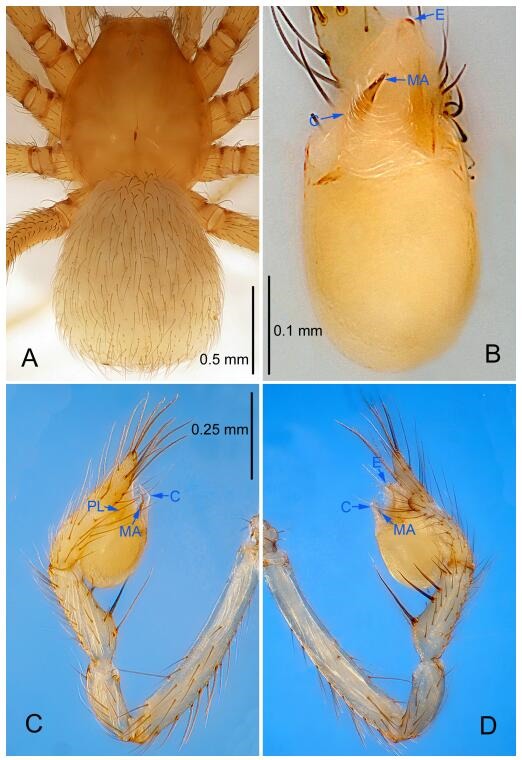
*Leptonetela lujia* sp. nov., holotype male

**Figure 51:**
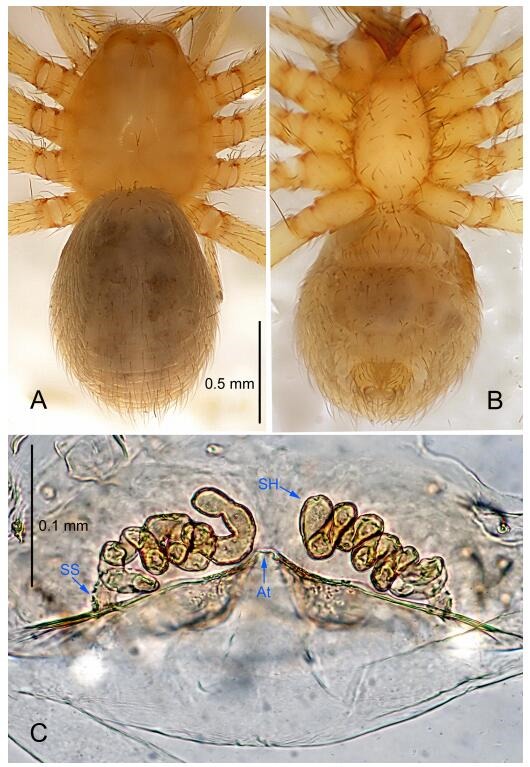
*Leptonetela lujia* sp. nov., one of the paratype females

**Type material. Holotype:** male (IZCAS), Wuming Cave, N26.48º, E107.54º, Lujia Bridge, Gubin, Xingshan Town, Majiang County, Kaili City, Guizhou Province, China, 29 November 2011, H. Chen & Z. Zha leg. **Paratypes:** 1 male and 2 females, same data as holotype.

**Etymology.** The specific name refers to the type locality; noun.

**Diagnosis.** This new species is similar to *L. jinsha* Lin et Li, 2010, *L. quinquespinata* (Chen & Zhu, 2008), *L. xinhua* Wang & Li **sp. nov.** and *L. gubin* Wang & Li **sp. nov.** but can be distinguished by the male pedipalpal tibia with 4 long setae prolaterally, 5 slender spines retrolaterally, with spines Ⅰ longest, spines Ⅱ Ⅲ equal length (Figure 50D), conductor thin, triangular (Figure 50B), (tibia with 3 long setae prolaterally, 1 long setae and 5 spines retrolaterally, with spines Ⅰ strongest, tip asymmetrically bifurcated, conductor broad, distal edge undulate in *L. jinsha*; tibia with 3 long setae prolaterally, 6 slender spines retrolaterally, with spines Ⅰ longest, conductor semicircular in *L. quinquespinata*; embolus bifurcated, tibia with 5 slender spines prolaterally, 5 slender spines retrolaterally, conductor triangular in *L. xinhua* Wang & Li **sp. nov.**; tibia with 4 slender spines prolaterally, 5 slender spines retrolaterally, spines Ⅰ, Ⅱ equal length, cymbium with 2 long, curved spines on earlobe-shaped process retrolaterally, conductor semicircular in *L. gubin* Wang & Li **sp. nov.**); from *L. gubin* and *L. quinquespinata* by the slightly swollen base of the median apophysis (Figure 50B) (base of median apophysis distinctly swollen, 4 times the width of tip in *L. gubin* Wang & Li **sp. nov.**; 3 times in *L. quinquespinata*).

**Description. Male (holotype).** Total length 1.72 (Figure 50A). Carapace 0.90 long, 0.85 wide. Opisthosoma 0.87 long, 0.88 wide. Carapace yellow. Ocular area with a pair of setae, eye absent. Median groove needle-shaped, cervical grooves and radial furrows indistinct. Clypeus 0.12 high. Opisthosoma yellowish, ovoid. Leg measurements: Ⅰ 7.75 (2.10, 0.37, 2.23, 1.78, 1.27); Ⅱ 6.97 (1.96, 0.37, 1.86, 1.62, 1.16); Ⅲ 5.73 (1.62, 0.32, 1.50, 1.32, 0.97); Ⅳ 7.15 (2.02, 0.30, 1.92, 1.80, 1.11). Male pedipalp (Figure 50C-D): tibia with 4 long spines prolaterally, 5 large spines retrolaterally, with spines Ⅰ longest, spines Ⅱ Ⅲ equal length. Cymbium not constricted medially, earlobe-shaped process distinct. Embolus triangular, prolateral lobe indistinct. Median apophysis index finger like. Conductor thin, triangular in ventral view (Figure 50B).

**Female (one of the paratypes).** Similar to male in color and general features, but larger and with shorter legs. Total length 1.70 (Figure 51A-B). Carapace 0.85 long, 0.75 wide. Opisthosoma 0.87 long, 0.83 wide. Clypeus 0.12 high. Leg measurements: Ⅰ 6.89 (1.85, 0.37, 1.97, 1.50, 1.20); Ⅱ 5.94 (1.67, 0.35, 1.62, 1.25, 1.05); Ⅲ 5.30 (1.48, 0.35, 1.38, 1.22, 0.87); Ⅳ 6.47 (1.86, 0.37, 1.70, 1.46, 1.08). Vulva (Figure 51C): spermathecae coiled, atrium triangular.

**Distribution.** China (Guizhou).

### *Leptonetela xinhua* Wang & Li sp. nov. Figures 52-53, 97

**Figure 52:**
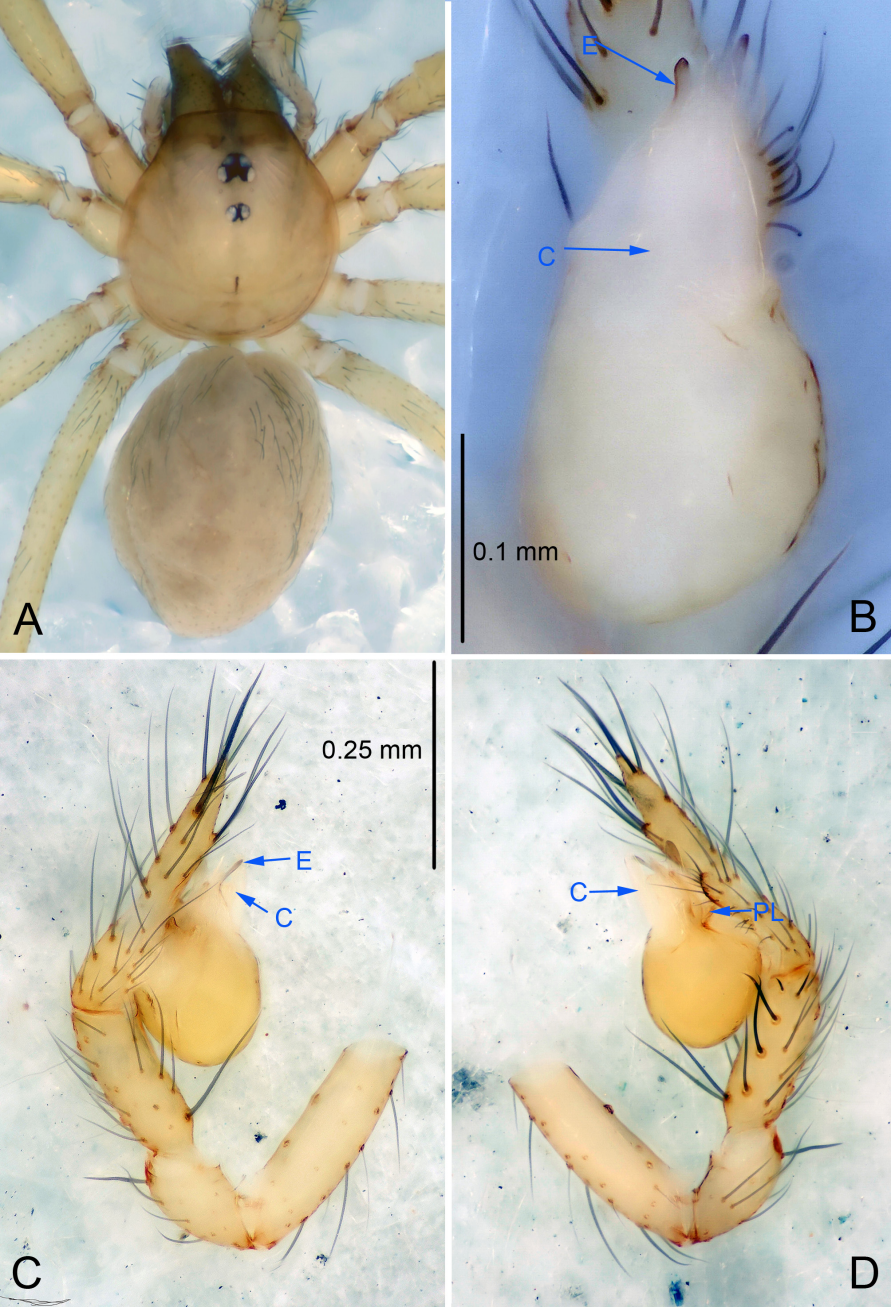
*Leptonetela xinhua* sp. nov., holotype male

**Figure 53:**
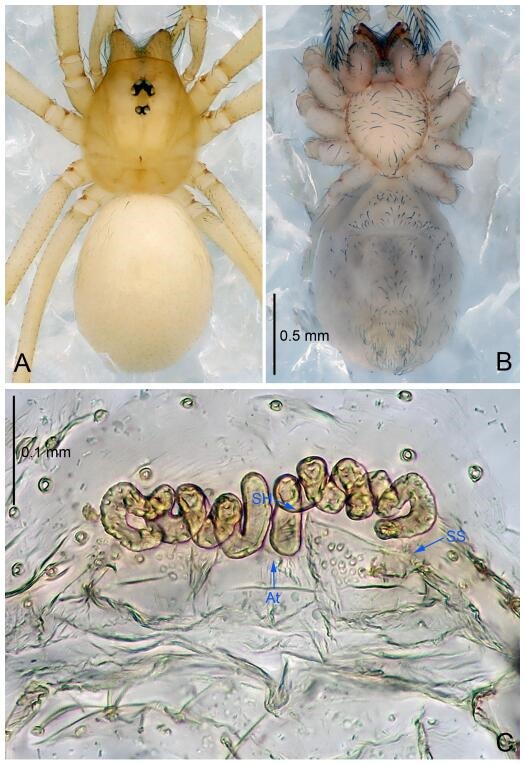
*Leptonetela xinhua* sp. nov., one of the paratype females

**Type material. Holotype:** male (IZCAS), nameless Cave, N27.85º, E111.31º, Caojia Town, Xinhua County, Loudi City, Hunan Province, China, 24 March 2016, Y. Li & Z. Chen leg. **Paratypes:** 3 males and 2 females, same data as holotype.

**Etymology.** The specific name refers to the type locality; noun.

**Diagnosis.** This new species is similar to *L. jinsha* Lin & Li, 2010, *L. quinquespinata* (Chen & Zhu, 2008), *L. lujia* Wang & Li **sp. nov.**, and *L. gubin* Wang & Li **sp. nov.**, but can be distinguished by the bifurcated embolus, male pedipalpal tibia with 5 slender spines prolaterally, 5 slender spines retrolaterally, conductor triangular (Figure 52D), (tibia with 4 long setae prolaterally, 5 slender spines retrolaterally, with spines Ⅰ longest, spines Ⅱ Ⅲ equal length, conductor thin, triangular in *L. lujia* Wang & Li **sp. nov.**; tibia with 3 long setae prolaterally, 1 long seta and 5 spines retrolaterally, with spines Ⅰ strongest, tip asymmetrically bifurcated, conductor broad, distal edge undulate in *L. jinsha*; tibia with 3 long setae prolaterally, 6 slender spines retrolaterally, with spines Ⅰ longest, conductor semicircular in *L. quinquespinata*; tibia with 4 slender spines prolaterally, 5 slender spines retrolaterally, with spines Ⅰ, Ⅱ equal length, cymbium with 2 long, curved spines on earlobe-shaped process retrolaterally, conductor semicircular in *L. gubin* Wang & Li **sp. nov.**); from *L. gubin* and *L. quinquespinata* by the base of median apophysis slightly swollen (Figure 52B) (base of median apophysis distinctly swollen, 4 times the width of tip in *L. gubin* Wang & Li **sp. nov.**; 3 times in *L. quinquespinata*).

**Description. Male (holotype):** total length 1.78 (Figure 52A). Prosoma 0.85 long, 0.71 wide. Opisthosoma 0.94 long, 0.73 wide. Prosoma yellowish. Ocular area with a pair of setae, six eyes. Median groove needle-shaped, brown. Cervical grooves and radial furrows indistinct. Clypeus 0.14 high, slightly sloped anteriorly. Opisthosoma pale brown, ovoid, covered with short hairs, lacking distinct pattern. Sternum and legs yellowish. Leg measurements: Ⅰ 5.39 (1.52, 0.28, 1.58, 1.20, 0.81); Ⅱ 4.37 (1.28, 0.29, 1.28, 1.01, 0.51); Ⅲ 3.84 (1.03, 0.25, 0.98, 0.95, 0.63); Ⅳ5.15 (1.36, 0.27, 1.50, 1.23, 0.79). Male pedipalp (Figure 52C-D): tibia with 5 slender spines prolaterally, 5 slender spines retrolaterally. Cymbium with an earlobe-shaped process retrolaterally. Embolus bifurcated, prolateral lobe triangular. Median apophysis tongue shaped in prolateral view. Conductor triangular in ventral view (Figure 52B).

**Female (one of the paratypes):** similar to male in color and general features, but with a larger body size and shorter legs. Total length 1.95 (Figure 53A-B). Prosoma 0.66 long, 0.53 wide. Opisthosoma 1.06 long, 0.86 wide. Clypeus 0.20 high. Leg measurements: Ⅰ 4.60 (1.30, 0.27, 1.33, 0.99, 0.71); Ⅱ 3.86 (1.11, 0.25, 1.01, 0.85, 0.64); Ⅲ 3.34 (0.95, 0.24, 0.83, 0.76, 0.56); Ⅳ 4.54 (1.33, 0.26, 1.25, 1.03, 0.67). Vulva (Figure 53C): spermathecae coiled, atrium fusiform.

**Distribution.** China (Hunan).

### *Leptonetela kangsa* Wang & Li sp. nov. Figures 54-55, 97

**Figure 54:**
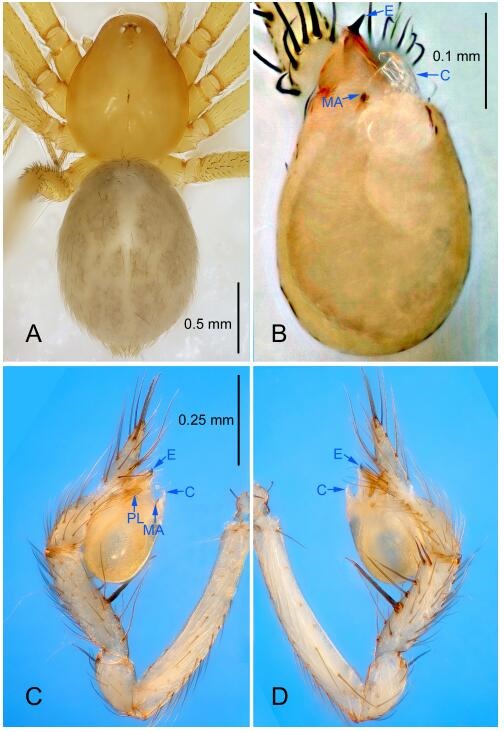
*Leptonetela kangsa* sp. nov., holotype male

**Figure 55:**
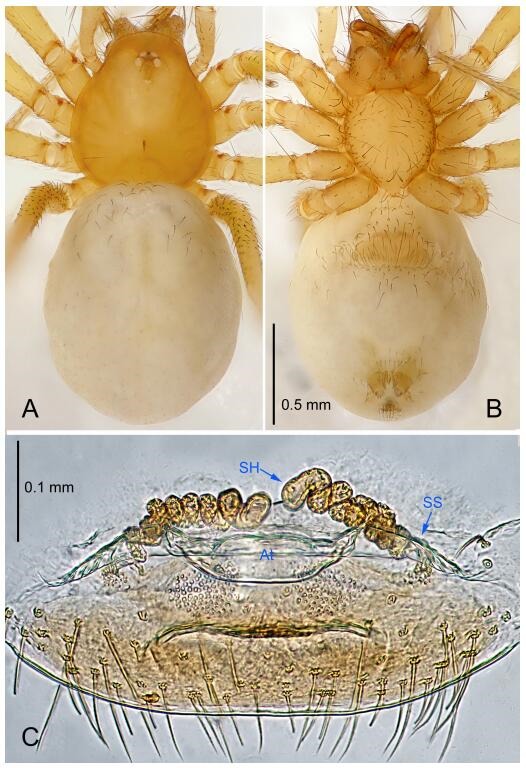
*Leptonetela kangsa* sp. nov., one of the paratype females

**Type material. Holotype:** male (IZCAS), Kangsagulie Cave, N26.79º, E108.21º, Datang, Geyi Town, Taijiang County, Kaili City, Guizhou Province, China, 5 December 2011, H. Chen & Z. Zha leg. **Paratypes:** 7 males and 6 females, same data as holotype.

**Etymology.** The specific name refers to the type locality; noun.

**Diagnosis.** This new species is similar to *L. shibingensis* and *L. wuming* Wang & Li **sp. nov.**, but can be distinguished by the median apophysis index finger-like in prolaterally view, tip bifurcated (Fig. 54D) (median apophysis small, triangular in ventral view in *L. shibingensis*; median apophysis like victory gesture, "Ⅴ" shaped in *L. wuming* Wang & Li **sp. nov.**); from *L. wuming* Wang & Li **sp. nov.**, by the tibia Ⅰ spine located at the middle of tibia, embolus with 1 basal tooth (tibia Ⅰ spine located at the base of tibia, embolus without tooth in *L. wuming* Wang & Li **sp. nov.**).

**Description. Male (holotype).** Total length 2.07 (Figure 54A). Carapace 0.85 long, 0.87 wide. Opisthosoma 1.25 long, 0.92 wide. Carapace yellow. Eyes six, PME reduced to white spots. Median groove needle-shaped, cervical grooves and radial furrows indistinct. Clypeus 0.12 high. Opisthosoma gray, ovoid. Leg measurements: Ⅰ 9.04 (2.50, 0.37, 2.65, 2.10, 1.42); Ⅱ 7.43 (2.10, 0.36, 2.05, 1.70, 1.22); Ⅲ 6.23 (1.77, 0.37, 1.60, 1.47, 1.02); Ⅳ 8.09 (2.22, 0.35, 2.27, 2.00, 1.25). Male pedipalp (Figure 54C-D): tibia with 4 long setae prolaterally, 5 large spines retrolaterally, with Ⅰ spine strong, located medially. Cymbium constricted medially, attaching an earlobe-shaped process retrolaterally. Embolus triangular, bearing a basal tooth, prolateral lobe oval. Median apophysis index finger-like in prolateral view, tip bifurcated. Conductor bamboo leaf-shaped in ventral view (Figure 54B).

**Female (one of the paratypes).** Similar to male in color and general features, but smaller and with shorter legs. Total length 2.02 (Figure 55A-B). Carapace 0.72 long, 0.72 wide. Opisthosoma 1.27 long, 1.02 wide. Clypeus 0.15 high. Leg measurements: Ⅰ 7.61 (2.07, 0.35, 2.25, 1.72, 1.22); Ⅱ 6.23 (1.72, 0.35, 1.72, 1.37, 1.07); Ⅲ 5.41 (1.62, 0.32, 1.35, 1.22, 0.90); Ⅳ 6.92 (1.80, 0.35, 2.00, 1.65, 1.12). Vulva (Figure 55C): spermathecae coiled, atrium fusiform.

**Distribution.** China (Guizhou).

### *Leptonetela wuming* Wang & Li sp. nov. Figures 56-57, 97

**Figure 56:**
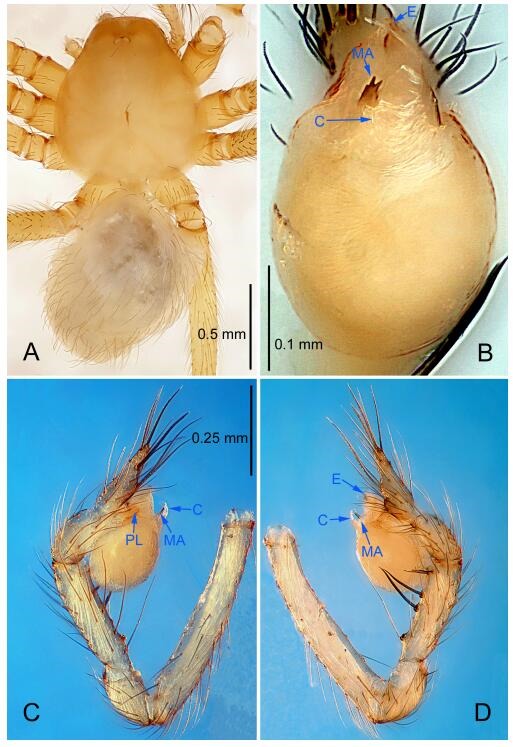
*Leptonetela wuming* sp. nov., holotype male

**Figure 57:**
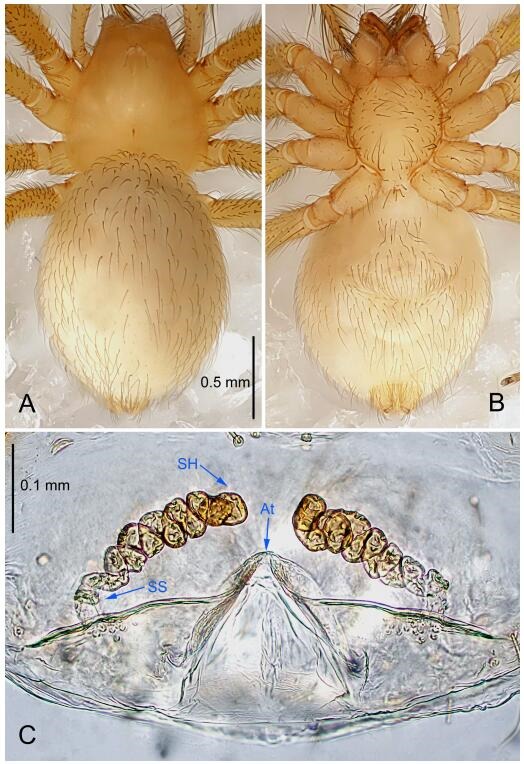
*Leptonetela wuming* sp. nov., one of the paratype females

**Type material. Holotype:** male (IZCAS), Wuming Cave, N25.43°, E105.62°, Dabei Town, Zhenfeng County, Guizhou Province, China, 18 July 2012, H. Zhao leg. **Paratypes:** 2 males and 5 females, same data as holotype.

**Etymology.** The specific name refers to the type locality; noun.

**Diagnosis.** This new species is similar to *L. kangsa* Wang & Li **sp. nov.**, and *L. shibingensis* but can be distinguished by the male pedipalpal bulb embolus without basal tooth (Figure 56B), (embolus with basal tooth in the two species mentioned above); from *L. kangsa* Wang & Li **sp. nov.** and *L. shibingensis* by the tibia spines Ⅰ located at the base of the tibia (Figure 56D) (tibia spines Ⅰ located medially in *L. kangsa* Wang & Li **sp. nov.** and *L. shibingensis*).

**Description. Male (holotype).** Total length 1.50 (Figure 56A). Carapace 0.60 long, 0.45 wide. Opisthosoma 1.10 long, 0.60 wide. Carapace yellowish. Ocular area with a pair of setae, PLE, PME absent, ALE reduced to white spots. Median groove needle-shaped, cervical grooves and radial furrows indistinct. Clypeus 0.09 high. Opisthosoma yellowish, ovoid. Leg measurements: Ⅰ 11.75 (3.12, 0.35, 3.40, 2.88, 2.00); Ⅱ 9.35 (2.75, 0.35, 2.48, 2.49, 1.28); Ⅲ 8.47 (2.80, 0.32, 2.30, 1.96, 1.09); Ⅳ 9.81 (2.81, 0.35, 2.72, 2.56, 1.37). Male pedipalp (Figure 56C-D): tibia with 3 long setae prolaterally, 5 large spines retrolaterally, with spines Ⅰ strong, longest. Cymbium constricted medially, attached to an earlobe-shaped process retrolaterally. Embolus triangular, prolateral lobe oval. Median apophysis like victory gesture, "Ⅴ" shaped. Conductor bamboo leaf-shaped in ventral view (Figure 56B).

**Female (one of the paratypes).** Similar to male in color and general features, but larger and with shorter legs. Total length 3.02 (Figure 57A-B). Carapace 1.25 long, 0.90 wide. Opisthosoma 2.45 long, 1.38 wide. Clypeus 0.09 high. Leg measurements: Ⅰ 8.67 (2.44, 0.33, 2.50, 2.01, 1.39); Ⅱ 7.29 (2.10, 0.33, 2.08, 1.65, 1.13); Ⅲ 7.22 (2.08, 0.35, 2.03, 1.63, 1.13); Ⅳ 7.30 (2.51, 0.33, 1.85, 1.56, 1.05). Vulva (Figure 57C): spermathecae coiled, atrium triangular.

**Distribution.** China (Guizhou).

### *Leptonetela shanji* Wang & Li Wang & Li sp. nov. Figures 58-59, 97

**Figure 58:**
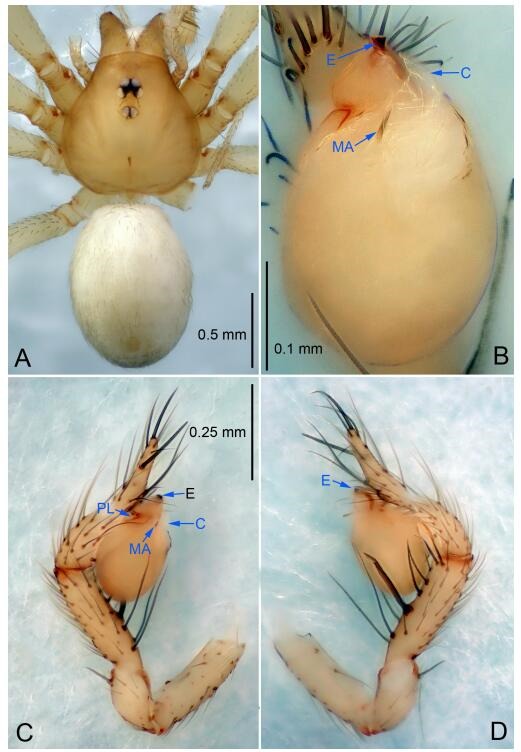
*Leptonetela shanji* sp. nov., holotype male

**Figure 59:**
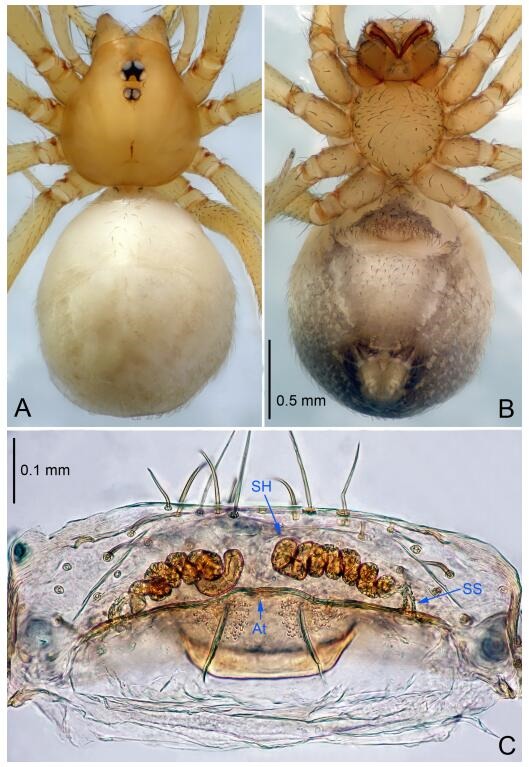
*Leptonetela shanji* sp. nov., one of the paratype females

**Type material. Holotype:** male (IZCAS), Shanji Cave, N27.28°, E107.82°, Xiaguihua, Xiaosai Town, Yuqing County, Zunyi City, Guizhou Province, China, 15 August 2012, H. Zhao leg. **Paratypes:** 3 males and 3 females, same data as holotype; 2 females, Guanyin Cave, N27.32°, E107.71°, Hongjun, Longxi Town, Yuqing County, Zunyi City, Guizhou Province, China, 15 August 2012, H. Zhao leg; 3 females, Liangfeng Cave, N27.27°, E107.76°, Xiaosai Town, Yuqing County, Zunyi City, Guizhou Province, China, 14 August 2012, H. Zhao leg.

**Etymology.** The specific name refers to the type locality; noun.

**Diagnosis.** This new species is similar to *L. digitata* Lin & Li, 2010, *L. hamata* Lin & Li, 2010 and *L. tetracantha* Lin & Li, 2010, but can be distinguished by the male pedipalpal tibia spines Ⅰ strong, located medially (Figure 58D) (tibia spines Ⅰ slender, located at the base of tibia in all above); from *L. hamata* and *L. tetracantha* by the male pediapal tibia spines Ⅰ asymmetrically bifurcated (Figure 58D) (tibia spines Ⅰ not bifurcated in *L. hamata*, and *L. tetracantha*); from *L. digitata* by themedian apophysis not curved (median apophysis curved in *L. digitata*).

**Description. Male (holotype).** Total length 2.08 (Figure 58A). Carapace 0.90 long, 0.95 wide. Opisthosoma 1.10 long, 0.83 wide. Carapace yellow. Ocular area with a pair of setae, six eyes. Median groove needle-shaped, cervical grooves and radial furrows indistinct. Clypeus 0.13 high. Opisthosoma gray, ovoid. Leg measurements: Ⅰ 8.54 (2.25, 0.38, 2.50, 2.03, 1.38); Ⅱ 6.89 (1.88, 0.38, 1.88, 1.60, 1.15); Ⅲ 5.70 (1.55, 0.35, 1.47, 1.35, 0.98); Ⅳ 7.59 (2.03, 0.38, 2.13, 1.88, 1.17). Male pedipalp (Figure 58C-D): tibia with 4 long spines prolaterally, 5 spines retrolaterally, with Ⅰ spine strong, asymmetrically bifurcated and located at the base of tibia. Cymbium constricted medially, attached to an earlobe-shaped process retrolaterally. Embolus triangular, bearing a small basal tooth, prolateral lobe oval. Median apophysis index finger like in prolateral view, tapering. Conductor bamboo leaf-shaped in ventral view (Figure 58B).

**Female (one of the paratypes).** Similar to male in color and general features, but larger and with shorter legs. Total length 2.40 (Figure 59A-B). Carapace 0.95 long, 0.88 wide. Opisthosoma 1.38 long, 1.25 wide. Clypeus 0.13 high. Leg measurements: Ⅰ 7.97 (2.13, 0.38, 2.38, 1.75, 1.33); Ⅱ 6.36 (1.75, 0.35, 1.75, 1.38, 1.13); Ⅲ 5.31 (1.45, 0.35, 1.38, 1.25, 0.88); Ⅳ 7.18 (1.95, 0.38, 2.00, 1.70, 1.15). Vulva (Figure 59C): spermathecae coiled, atrium fusiform.

**Distribution.** China (Guizhou).

### *Leptonetela xiaoyan* Wang & Li sp. nov. Figures 60-61, 97

**Figure 60:**
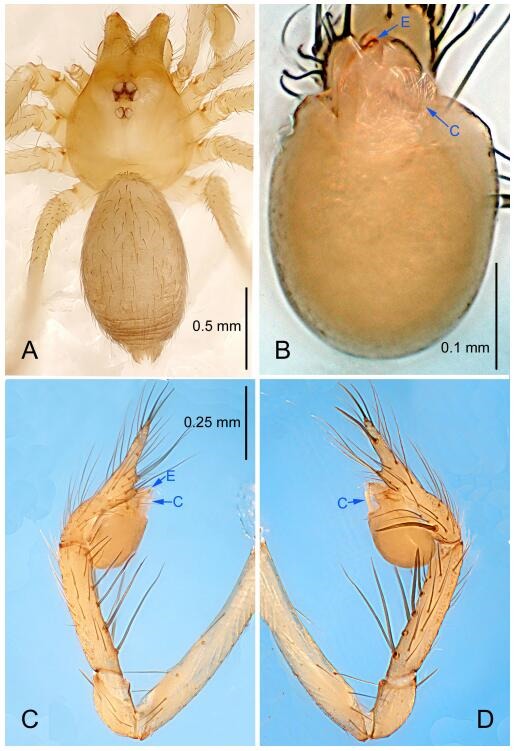
*Leptonetela xiaoyan* sp. nov., holotype male

**Figure 61:**
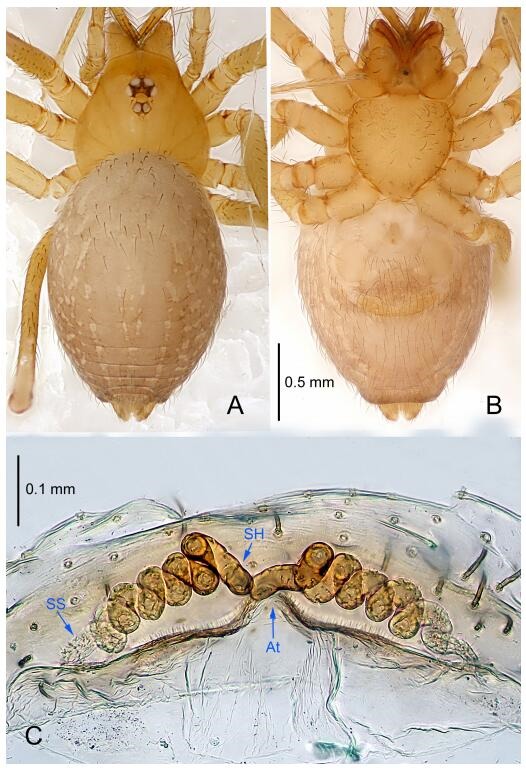
*Leptonetela xiaoyan* sp. nov., one of the paratype females

**Type material. Holotype:** male (IZCAS), Gejiaxiaoyan Cave, N27.11°, E105.24°, Shanjiao, Zhuchang Town, Bijie City, Guizhou Province, China, 27 January 2011, H. Chen & Z. Zha leg. **Paratypes:** 2 males and 6 females, same data as holotype.

**Etymology.** The specific name refers to the type locality; noun.

**Diagnosis.** This new species is similar to *L. curvispinosa* Lin & Li, 2010, but can be distinguished by the male pedipalpal tibia with 4 large spines prolaterally, 6 large spines retrolaterally (Figure 60D), median apophysis not sclerotized, little finger-shaped in prolateral view, conductor broad C tile-shaped (Figure 60B) (tibia with 3 large spines prolaterally, 5 large spines retrolaterally, median apophysis absent, conductor reduced in *L. curvispinosa*).

**Description. Male (holotype).** Total length 1.67 (Figure 60A). Carapace 0.88 long, 0.80 wide. Opisthosoma 1.15 long, 0.75 wide. Carapace yellowish. Six eyes. Median groove, cervical grooves and radial furrows indistinct. Clypeus 0.10 high. Opisthosoma yellowish, ovoid. Leg measurements: Ⅰ 9.96 (2.84, 0.35, 2.80, 2.40, 1.57); Ⅱ 7.09 (2.02, 0.32, 2.00, 1.60, 1.15); Ⅲ 6.38 (1.77, 0.32, 1.82, 1.47, 1.00); Ⅳ 7.89 (2.25, 0.35, 2.27, 1.85, 1.17). Male pedipalp (Figure 60C-D): tibia with 4 spines prolaterally and 6 spines retrolaterally, with Ⅰ spine longest. Cymbium not constricted, prolaterally with one curved spine on the base. Embolus triangular, prolateral lobe oval. Median apophysis slightly sclerotized, fingerlike in prolateral view. Conductor broad, C tile-shaped in ventral view (Figure 60B).

**Female (one of the paratypes).** Similar to male in color and general features, but larger and with shorter legs. Total length 2.12 (Figure 61A-B). Carapace 0.82 long, 0.82 wide. Opisthosoma 1.47 long, 1.12 wide. Clypeus 0.15 high. Leg measurements: Ⅰ 9.79 (2.62, 0.35, 3.03, 2.22, 1.57); Ⅱ 6.84 (1.90, 0.30, 1.97, 1.47, 1.20); Ⅲ 5.53 (1.52, 0.32, 1.52, 1.27, 0.90); Ⅳ 7.36 (2.07, 0.35, 1.97, 1.72, 1.25). Vulva (Figure 61C): spermathecae coiled, atrium triangular, anterior margin of atrium covered with short hairs.

**Distribution.** China (Guizhou).

### *Leptonetela huoyan* Wang & Li sp. nov. Figures 62-63, 97

**Figure 62:**
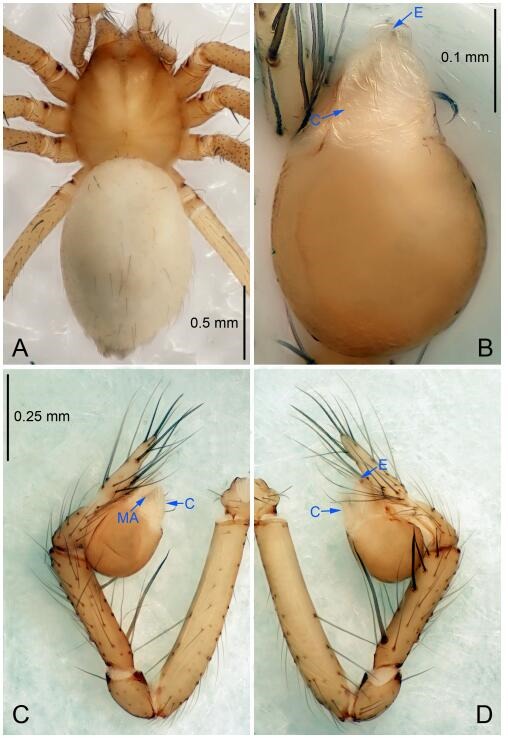
*Leptonetela huoyan* sp. nov., holotype male

**Figure 63:**
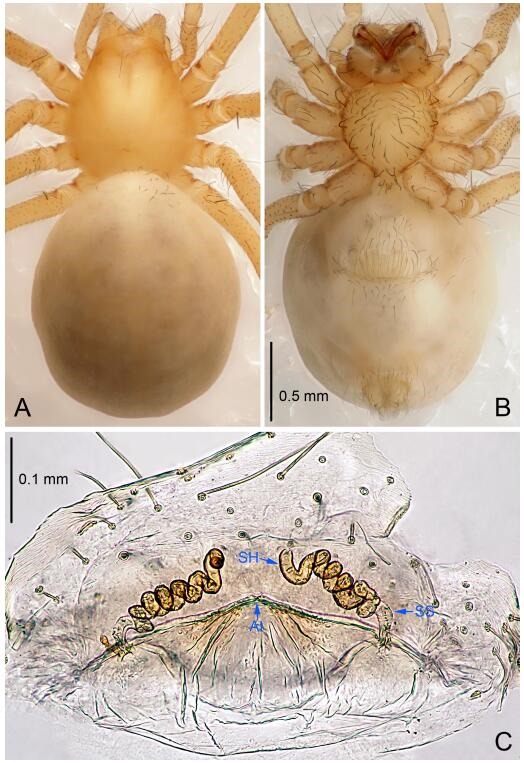
*Leptonetela huoyan* sp. nov., one of the paratype females

**Type material. Holotype:** male (IZCAS), Heyuantou nameless Cave, N29.25°, E109.35°, Huoyan Street, Guitang Dam Town, Longshan County, Hubei Province, China, 15 January 2014, Y. Li & Y. Lin leg. **Paratypes:** 1 male and 2 females, same data as holotype; 1 male and 4 females, nameless Cave, N29.61°, E109.17° Jieping, Xianfeng County, Enshi Tujia and Miao Autonomous Prefecture, Hubei Province, China, 17 January 2014, Y. Li & Y. Lin leg.

**Etymology.** The specific name refers to the type locality; noun.

**Diagnosis.** This new species is similar to *L. anshun* Lin & Li, 2010 and *L. chenjia* Wang & Li **sp. nov.**, but can be distinguished by on the male pedipalpal bulb median apophysis slightly sclerotized, index finger like, conductor broad, semicircular (Fig 62B) (median apophysis absent in *L. anshun*, and *L. chenjia* Wang & Li **sp. nov.**; tip of conductor bifurcated in *L. anshun*, conductor reduced in *L. chenjia* Wang & Li **sp. nov.**); from *L. anshun* by the tibia spines Ⅰ slender (Figure 62D) (tibia spines Ⅰ strong, tip bifurcated in *L. anshun*).

**Description. Male (holotype).** Total length 2.25 (Figure 62A). Carapace 0.88 long, 0.83 wide. Opisthosoma 1.50 long, 0.92 wide. Carapace yellow. Eye absent. Median groove, cervical groove and radial furrows indistinct. Clypeus 0.13 high. Opisthosoma gray, ovoid. Leg measurements: Ⅰ 8.90 (2.53, 0.40, 2.57, 2.00, 1.40); Ⅱ 7.69 (2.38, 0.40, 2.13, 1.65, 1.13); Ⅲ 6.51 (2.00, 0.38, 1.75, 1.50, 0.88); Ⅳ 7.73 (2.25, 0.40, 2.15, 1.78, 1.15). Male pedipalp (Figure 62C-D): tibia with 4 long setae prolaterally, 1 long seta and 5 spines retrolaterally, with spines Ⅰ longest, distant from others, the rest of the spines concentrated distally. Cymbium constricted medially, attached to an earlobe-shaped process retrolaterally. Embolus triangular, prolateral lobe absent. Median apophysis slightly sclerotized, index finger like. Conductor broad, semicircular in ventral view (Figure 62B).

**Female (one of the paratypes).** Similar to male in color and general features, but larger and with shorter legs. Total length 2.53 (Figure 63A-B). Carapace 0.93 long, 0.82 wide. Opisthosoma 1.63 long, 1.38 wide. Clypeus 0.15 high. Leg measurements: Ⅰ 8.26 (2.38, 0.38, 2.32, 1.88, 1.30); Ⅱ 7.28 (2.05, 0.35, 2.00, 1.63, 1.25); Ⅲ 6.47 (1.93, 0.35, 1.62, 1.47, 1.10); Ⅳ 7.54 (2.20, 0.38, 2.08, 1.75, 1.13). Vulva (Figure 63C): spermathecae coiled, atrium fusiform.

**Distribution.** China (Hubei).

### *Leptonetela liuguan* Wang & Li sp. nov. Figures 64-65, 97

**Figure 64:**
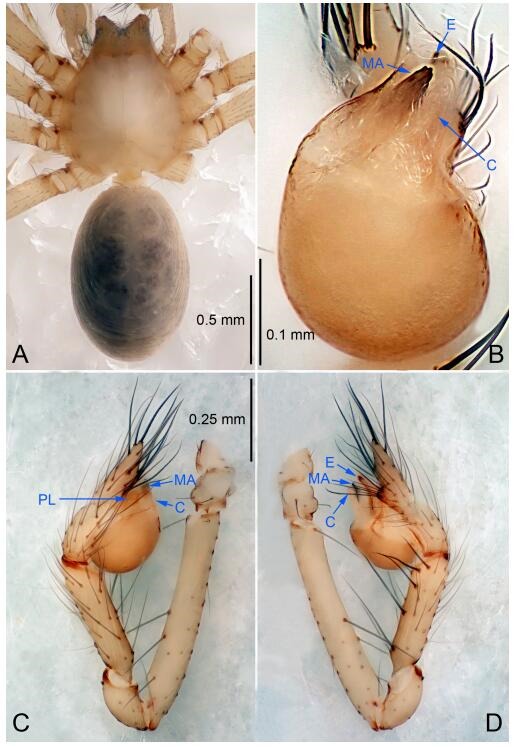
*Leptonetela liuguan* sp. nov., holotype male

**Figure 65:**
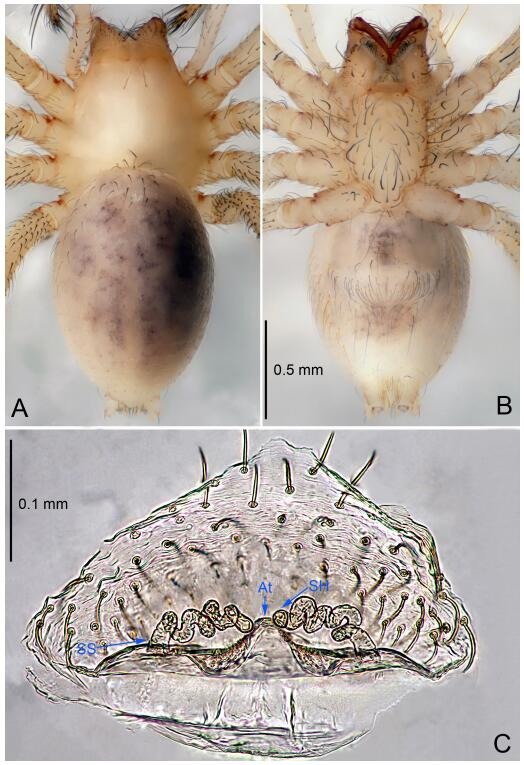
*Leptonetela liuguan* sp. nov., one of the paratype females

**Type material. Holotype:** male (IZCAS), Liuguan Cave, N26.15°, E106.46°, Mengqiu, Baiyunshan Town, Changshun County, Guizhou Province, China, 23 December 2010, Z. Zha & Z. Chen leg. **Paratypes:** 2 female, same data as holotype; 1 male, Fenghuang Cave, N26.09°, E106.39°, Shenglian, Zhonghuo Town, Changshun County, Guizhou Province, China, 23 December 2010, Z. Zha & Z. Chen leg.

**Etymology.** The specific name refers to the type locality; noun.

**Diagnosis.** This new species is similar to *L. penevi* Wang & Li, 2016 and *L. changtu* Wang & Li **sp. nov.** but can be distinguished by on the male pedipalpal bulb median apophysis long, and half the length of bulb (Figure 64B) (median apophysis short, 1/5 the length of bulb in *L. palmate*, and *L. changtu* Wang & Li **sp. nov.**); male pedipalpal tibia spines slender, equally strong (Figure 64D) (tibia spines Ⅰ Ⅱ equally strong, stronger than other spines in *L. penevi*, tibia spines Ⅰ Ⅱ Ⅲ equally strong, stronger than other spines in *L. changtu* Wang & Li **sp. nov.**).

**Description. Male (holotype).** Total length 1.88 (Figure 64A). Carapace 0.73 long, 0.75 wide. Opisthosoma 1.10 long, 0.88 wide. Carapace yellowish. Eyes absent. Median groove, cervical grooves and radial furrows indistinct. Clypeus 0.13 high. Opisthosoma gray, ovoid. Leg measurements: Ⅰ 8.93 (2.38, 0.40, 2.64, 2.13, 1.38); Ⅱ 7.84 (2.13, 0.40, 2.23, 1.78, 1.30); Ⅲ 6.56 (1.75, 0.38, 1.80, 1.63, 1.00); Ⅳ 8.04 (2.25, 0.40, 2.18, 1.88, 1.33). Male pedipalp (Figure 64C-D): tibia with 3 long setae prolaterally, 5 slender spines retrolaterally, the spines slim equally strong. Embolus triangular, prolateral lobe absent. Teeth of median apophysis reduced to sclerotized spots, conductor and median apophysis long, equal length and half the length of bulb (Figure 64C).

**Female (one of the paratypes).** Similar to male in color and general features, but larger and with shorter legs. Total length 2.08 (Figure 65A-B). Carapace 0.75 long, 0.75 wide. Opisthosoma 1.50 long, 0.88 wide. Clypeus 0.15 high. Leg measurements: Ⅰ 7.81 (2.13, 0.38, 2.30, 1.75, 1.25); Ⅱ 6.89 (1.88, 0.38, 2.00, 1.50, 1.13); Ⅲ 5.97 (1.70, 0.38, 1.63, 1.38, 0.88); Ⅳ 7.27 (2.03, 0.38, 2.05, 1.63, 1.18). Vulva (Figure 65C): spermathecae coiled, atrium fusiform, anterior margin of atrium undulate.

**Distribution.** China (Guizhou).

### *Leptonetela nanmu* Wang & Li sp. nov. Figures 66-67, 97

**Figure 66:**
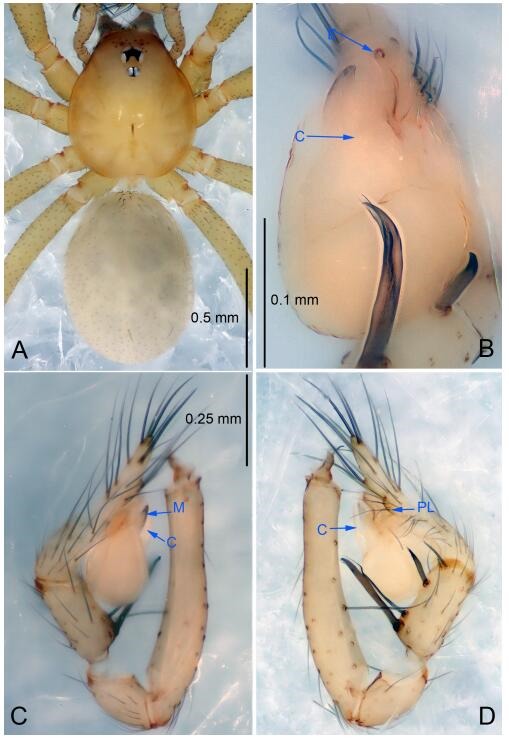
*Leptonetela nanmu* sp. nov., holotype male

**Figure 67:**
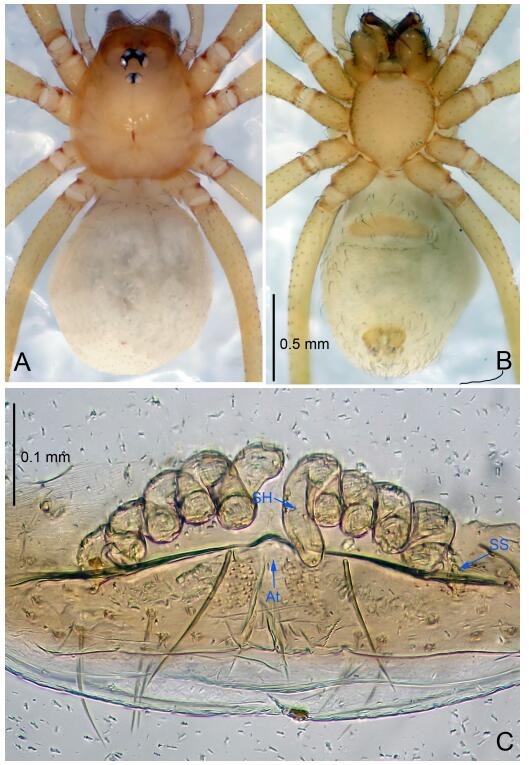
*Leptonetela nanmu* sp. nov., one of the paratype females

**Type material. Holotype:** male (IZCAS), Nanmu Cave, N28.10°, E110.08°, Pushi Town, Luxi County, Hunan Province, China, 5 April 2016, Y. Li & Z. Chen leg. **Paratypes:** 3 males and 2 females, same data as holotype.

**Etymology.** The specific name refers to the type locality; noun.

**Diagnosis.** This new species is similar to *L. tianxingensis*, but can be distinguished by on the male pedipalpal bulb conductor longer than median apophysis (Figure 66B) (conductor shorter than median apophysis in *L. tianxingensis*); male pedipalpal tibia Ⅲ spine strong (Figure 66D) (tibia Ⅲ spine slender in *L. tianxingensis*).

**Description. Male (holotype):** total length 1.70 (Figure 66A). Prosoma 0.81 long, 0.63 wide. Opisthosoma 0.94 long, 0.70 wide. Prosoma yellow. Six eyes, with a pair of setae on ocular area. Median groove needle-shaped, brown. Cervical grooves and radial furrows indistinct. Clypeus 0.14 high, slightly sloped anteriorly. Opisthosoma pale brown, ovoid, covered with short hairs, lacking distinctive pattern. Sternum and legs yellowish. Leg measurements: Ⅰ 5.45 (1.42, 0.26, 1.62, 1.27, 0.88); Ⅱ 4.76 (1.24, 0.25, 1.20, 1.27, 0.80); Ⅲ 4.12 (1.03, 0.23, 1.02, 0.95, 0.89); Ⅳ 5.60 (1.36, 0.22, 1.48, 1.27, 1.27). Male pedipalp (Figure 66C-D): tibia with 5 spines retrolaterally, with Ⅰ spine strongest, tip bifurcated, spines Ⅱ slender, spines Ⅲ strong. Embolus triangular, prolateral lobe oval. Median apophysis slightly sclerotized, thumb-shaped in ventral view. Conductor triangular, longer than median apophysis (Figure 66B).

**Female (one of the paratypes):** similar to male in color and general features, but with a larger body size and longer legs. Total length 1.98 (Figure 67A-B). Prosoma 0.88 long, 0.79 wide. Opisthosoma 1.12 long, 1.03 wide. Clypeus 0.20 high. Leg measurements: Ⅰ 5.63 (1.52, 0.28, 1.68, 1.30, 0.85); Ⅱ 4.86 (1.44, 0.27, 1.33, 0.86, 0.96); Ⅲ 4.21 (1.31, 0.21, 1.06, 0.99, 0.64); Ⅳ 5.23 (1.47, 0.25, 1.50, 1.20, 0.81). Vulva (Figure 67C): spermathecae coiled, atrium triangular.

**Distribution.** China (Hunan).

### *Leptonetela changtu* Wang & Li sp. nov. Figure 68-69, 97

**Figure 68:**
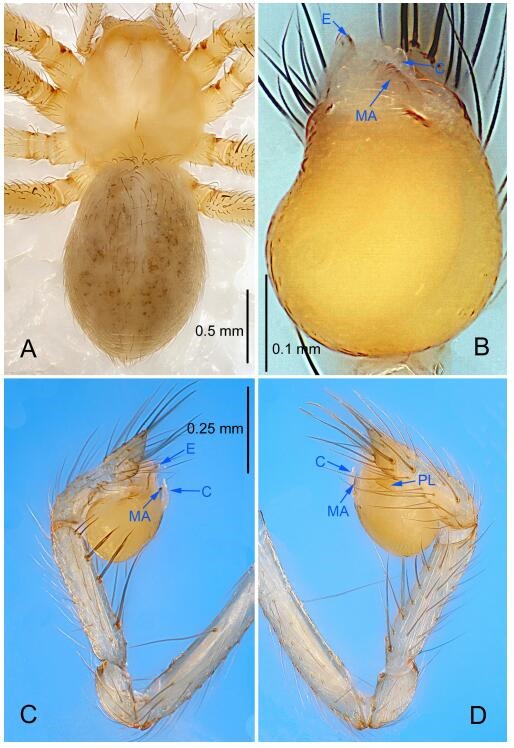
*Leptonetela changtu* sp. nov., holotype male

**Figure 69:**
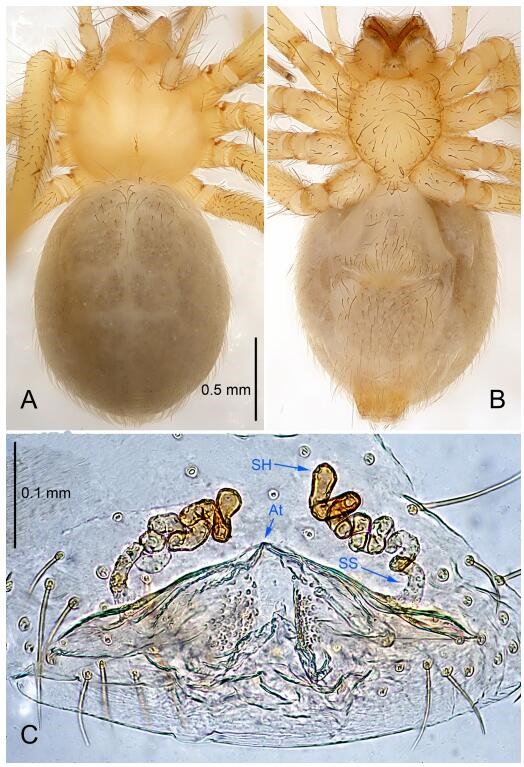
*Leptonetela changtu* sp. nov., one of the paratype females

**Type material. Holotype:** male (IZCAS), Changtu Cave, N27.14°, E105.43°, Honglin, Qianxi Town, Bijie County, Guizhou Province, China, 23 November 2011, Z. Zha & Z. Zha leg. **Paratypes:** 1 male and 10 females, same data as holotype.

**Etymology.** The specific name refers to the type locality; noun.

**Diagnosis.** This new species is similar to *L. penevi* Wang & Li, 2016 and *L. liuguan* Wang & Li **sp. nov.** but can be distinguished by the male pedipalpal tibia spines Ⅰ, Ⅱ, Ⅲ equally strong, stronger than other two spines (Figure 68C) (tibia spines Ⅰ and Ⅱ equally strong, stronger than other spines in *L. penevi*, tibial spines slender, equally strong in *L. liuguan* Wang & Li **sp. nov.**); from *L. liuguan* Wang & Li **sp. nov.** by median apophysis short, 1/5 the length of bulb (Figure 68B) (median apophysis long, half the length of bulb in *L. liuguan* Wang & Li **sp. nov.**); from *L. penevi* by the cymbium not constricted (cymbium constricted medially in *L. penevi*).

**Description. Male (holotype).** Total length 2.33 (Figure 68A). Carapace 1.06 long, 1.03 wide. Opisthosoma 1.38 long, 1.08 wide. Carapace yellowish. Ocular area with a pair of setae, eyes absent. Median groove needle-shaped, cervical grooves and radial furrows indistinct. Clypeus 0.14 high. Opisthosoma pale yellow, ovoid, with brown spots. Leg measurements: Ⅰ 10.02 (2.69, 0.39, 2.91, 2.38, 1.65); Ⅱ 8.75 (2.37, 0.38, 2.49, 2.08, 1.43); Ⅲ 7.53 (2.15, 0.38, 1.98, 1.77, 1.25); Ⅳ 9.20 (2.56, 0.38, 2.50, 2.26, 1.50). Male pedipalp (Figure 68C-D): tibia with 5 large spines retrolaterally, tibia spines Ⅰ longest, spines Ⅰ and Ⅱ equally strong, stronger than others. Cymbium not constricted. Embolus triangular, prolateral lobe oval. Median apophysis palm-shaped, teeth of median apophysis reduced to sclerotized spots. Conductor semicircular (Figure 68B).

**Female (one of the paratypes).** Similar to male in color and general features, but larger and with shorter legs. Total length 2.70 (Figure 69A-B). Carapace 1.10 long, 0.88 wide. Opisthosoma 1.72 long, 1.48 wide. Clypeus 0.20 high. Leg measurements: Ⅰ 9.10 (2.54, 0.43, 2.66, 1.98, 1.49); Ⅱ 7.73 (2.21, 0.41, 2.21, 1.67, 1.23); Ⅲ 6.85 (1.99, 0.40, 1.88, 1.50, 1.08); Ⅳ 8.22 (2.38, 0.41, 2.28, 1.88, 1.27). Vulva (Figure 69C): spermathecae coiled, atrium triangular.

**Distribution.** China (Guizhou).

### *Leptonetela lianhua* Wang & Li sp. nov. Figures 70-71, 97

**Figure 70:**
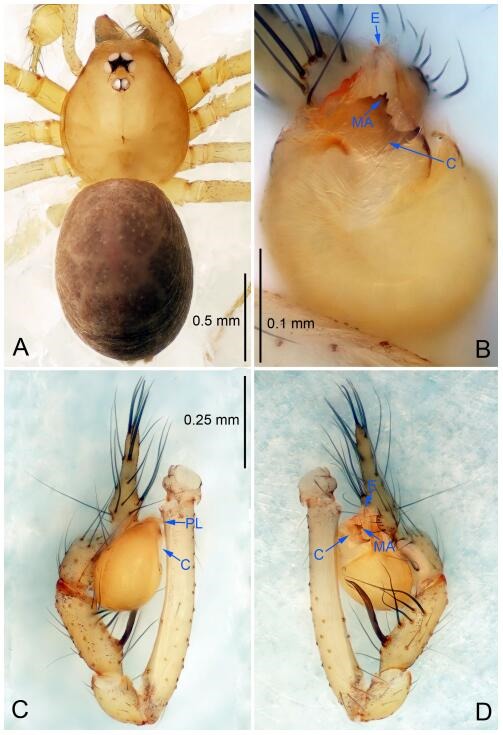
*Leptonetela lianhua* sp. nov., holotype male

**Figure 71:**
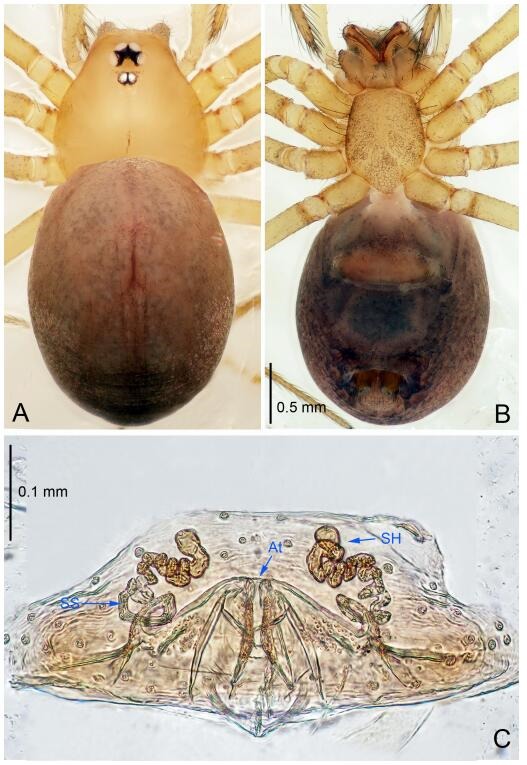
*Leptonetela lianhua* sp. nov., one of the paratype females

**Type material. Holotype:** male (IZCAS), Lianhua Cave, N25.48°, E114.09°, Niedou Town, Chongyi County, Jiangxi Province, China, 24 April 2013, Y. Luo & J. Liu leg. **Paratypes:** 3 males and 10 females, same data as holotype.

**Etymology.** The specific name refers to the type locality; noun.

**Diagnosis.** This new species is similar to *L. niubizi* Wang & Li **sp. nov.** but can be distinguished by the male pedipalpal tibia with 5 spines retrolaterally, with Ⅰ spine strongest, tip bifurcated, the other 4 spines slender, 2 of them longer than Ⅰ spine (Figure 70D); tip of median apophysis with 5 small teeth, and 1 ox horn-shaped large teeth (Figure 70B) (tibia with 5 slender spines retrolaterally, spines Ⅰ longest, not bifurcated, median apophysis antler-like, tip with 7 small teeth in *L. niubizi* Wang & Li **sp. nov.**).

**Description. Male (holotype).** Total length 2.00 (Figure 70A). Carapace 0.87 long, 0.70 wide. Opisthosoma 1.00 long, 0.87 wide. Carapace yellow. Ocular area with a pair of setae, six eyes. Median groove needle-shaped, cervical grooves and radial furrows indistinct. Clypeus 0.13 high. Opisthosoma brown, ovoid. Leg measurements: Ⅰ 9.69 (2.62, 0.25, 3.20, 2.37, 1.25); Ⅱ 7.05 (2.00, 0.25, 2.10, 1.70, 1.00); Ⅲ 5.70 (1.62, 0.22, 1.62, 1.37, 0.87); Ⅳ 7.45 (2.10, 0.25, 2.25, 1.75, 1.10). Male pedipalp (Figure 70C-D): tibia with 3 long setae prolaterally, and 5 spines retrolaterally, with spines Ⅰ strongest, tip bifurcated, and the other 4 spines slender, 2 of them longer than spines Ⅰ. Cymbium constricted medially, attached to an earlobe-shaped process retrolaterally. Embolus triangular, prolateral lobe absent. Tip of median apophysis with 5 small teeth, and 1 horn-shaped large teeth. Conductor broad C tile-shaped in ventral view (Figure 70B).

**Female (one of the paratypes).** Similar to male in color and general features, but larger and with shorter legs. Total length 2.25 (Figure 71A-B). Carapace 1.25 long, 0.95 wide. Opisthosoma 1.25 long, 0.75 wide. Clypeus 0.15 high. Leg measurements: Ⅰ 8.02 (2.12, 0.30, 2.50, 1.75, 1.35); Ⅱ 6.02 (1.65, 0.25, 1.75, 1.37, 1.00); Ⅲ 5.07 (1.35, 0.27, 1.50, 1.20, 0.75); Ⅳ 6.85 (2.00, 0.30, 1.95, 1.50, 1.10). Vulva (Figure 71C): spermathecae slender, coiled and atrium triangular.

**Distribution.** China (Jiangxi).

### *Leptonetela niubizi* Wang & Li sp. nov. Figures 72-73, 97

**Figure 72:**
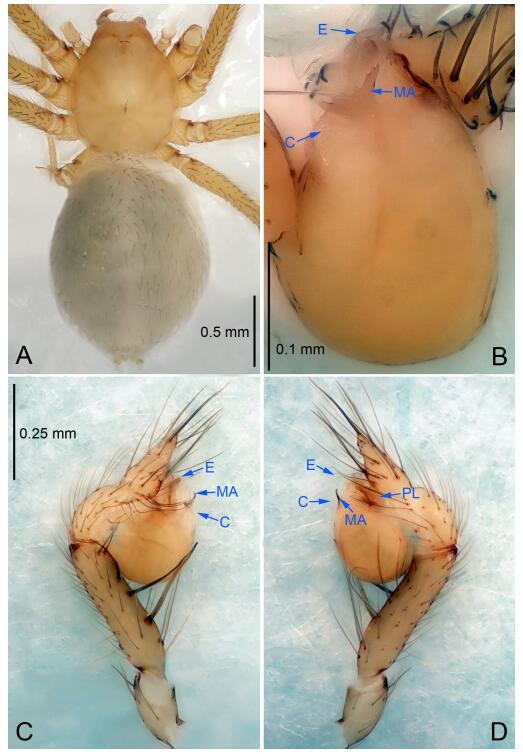
*Leptonetela niubizi* sp. nov., holotype male

**Figure 73:**
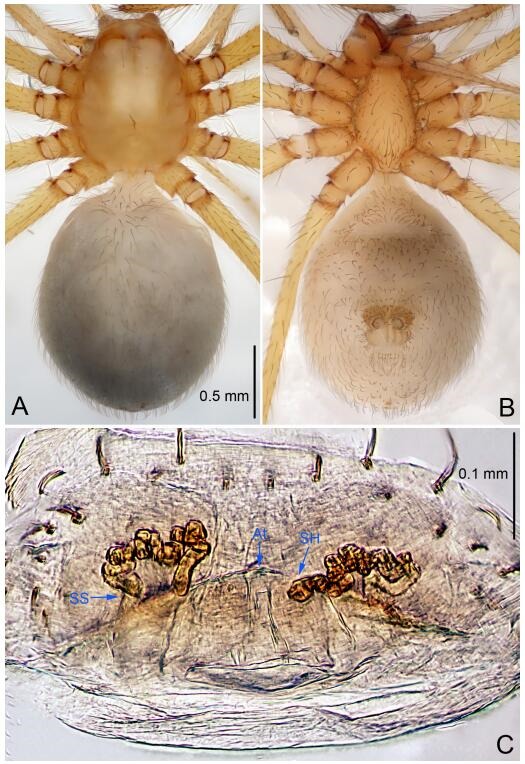
*Leptonetela niubizi* sp. nov., one of the paratype females

**Type material. Holotype:** male (IZCAS), Niubizi Cave, N27.62°, E106.67°, Leshan Town, Zunyi County, Zunyi City, Guizhou Province, China, 1 August 2012, H. Zhao leg. **Paratypes:** 7 females, same data as holotype.

**Etymology.** The specific name refers to the type locality; noun.

**Diagnosis.** This new species is similar to *L. lianhua* Wang & Li **sp. nov.** but can be distinguished by the male pedipalp tibia with 5 slender spines retrolaterally, spines Ⅰ longest, not bifurcated (Figure 72C), median apophysis antler-like, distal edge with 7 small teeth (Figure 72B) (tibia with 5 spines retrolateral, spines Ⅰ strongest, tip bifurcated, the other 4 spines slender, 2 of them longer than spines Ⅰ; tip of median apophysis decorated with 5 small teeth, and 1 horn-shaped large teeth in *L. lianhua* Wang & Li **sp. nov.**).

**Description. Male (holotype).** Total length 2.53 (Figure 72A). Carapace 0.95 long, 0.83 wide. Opisthosoma 1.58 long, 1.13 wide. Carapace yellowish. Ocular area with a pair of setae, eyes absent. Median groove needle-shaped, cervical grooves and radial furrows indistinct. Clypeus 0.15 high. Opisthosoma gray, ovoid. Leg measurements: Ⅰ 9.29 (2.56, 0.38, 2.63, 2.19, 1.53); Ⅱ 8.63 (2.50, 0.38, 2.34, 2.03, 1.38); Ⅲ 6.94 (2.03, 0.31, 1.47, 1.75, 1.38); Ⅳ -(2.55, 0.38, -, -, -). Male pedipalp (Figure 72C-D): tibia with 4 long setae prolaterally, 2 long setae and 5 slender spines retrolaterally, with spines Ⅰ longest. Cymbium constricted medially, attaching a small earlobe-shaped process retrolaterally. Embolus triangular, prolateral lobe oval. Median apophysis antler-like, distal edge decorated with 7 small teeth. Conductor short, C tile-shaped (Figure 72B).

**Female (one of the paratypes).** Similar to male in color and general features, but with a larger body size and shorter legs. Total length 2.60 (Figure 73A-B). Carapace 0.96 long, 0.95 wide. Opisthosoma 1.60 long, 1.25 wide. Clypeus 0.19 high. Leg measurements: Ⅰ 8.20 (2.34, 0.34, 2.44, 1.75, 1.33); Ⅱ 7.46 (2.25, 0.38, 1.88, 1.65, 1.30); Ⅲ 6.08 (2.05, 0.30, 1.08, 1.55, 1.10); Ⅳ 8.27 (2.38, 0.38, 2.25, 1.88, 1.38). Vulva (Figure 73C): spermathecae coiled, atrium triangular.

**Distribution.** China (Guizhou).

### *Leptonetela longyu* Wang & Li sp. nov. Figures 74-75, 97

**Figure 74:**
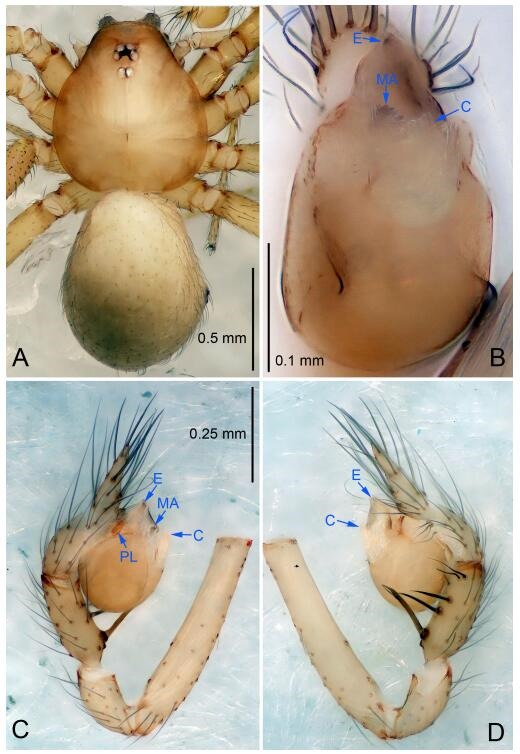
*Leptonetela longyu* sp. nov., holotype male

**Figure 75:**
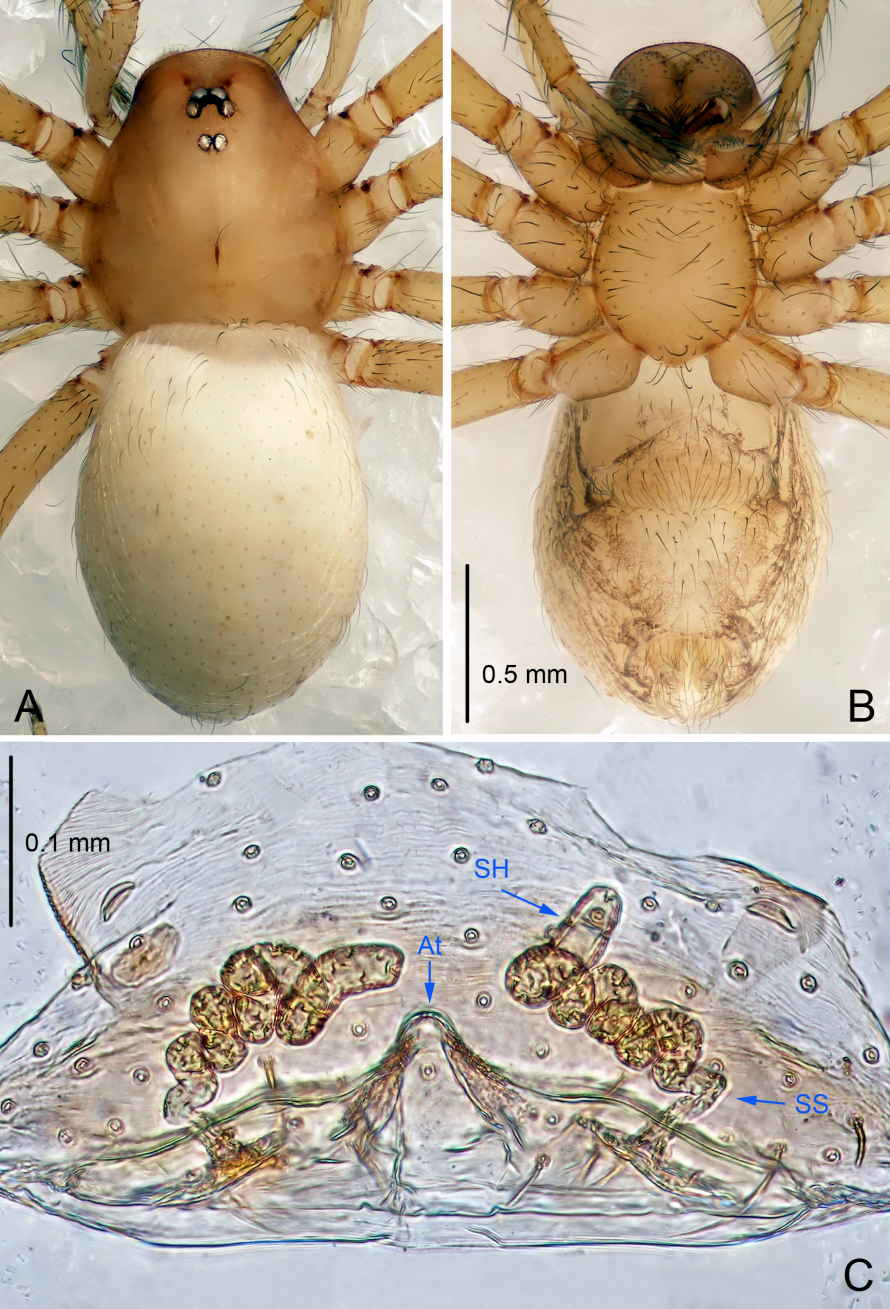
*Leptonetela longyu* sp. nov., one of the paratype females

**Type material. Holotype:** male (IZCAS), Longyu Cave, N29.40°, E110.09°, Cili County, Hunan Province, China, 5 June 2011, Z. Zha leg. **Paratypes:** 4 males and 5 females, same data as holotype, 5 males and 6 females, Niuerduo Cave, N29.404°, E110.73°, Cili County, Hunan Province, China, 9 April 2016, Y. Li & Z. Chen leg.

**Etymology.** The specific name refers to the type locality; noun.

**Diagnosis.** This new species is similar to *L. sexdentata* Wang & Li, 2011, *L. shicheng* Wang & Li **sp. nov.**, *L. zakou* Wang & Li **sp. nov.** and *L. meiwang* Wang & Li **sp. nov.** but can be distinguished by median apophysis harrow-like, tip with 5 small teeth (Figure 74B) (tip of median apophysis with 6 small teeth in *L. sexdentata* and *L. zakou* Wang & Li **sp. nov.**, 5 sharp teeth in *L. meiwang* Wang & Li **sp. nov.** and 10 in *L. shicheng* Wang & Li **sp. nov.**); from *L. shicheng* Wang & Li **sp. nov.** by the tip of conductor undulate (Figure 74B) (tip of conductor smooth in *L. shicheng* Wang & Li **sp. nov.**); from *L. zakou* Wang & Li **sp. nov.** by the teeth of median apophysis needle-shaped in *L. zakou* Wang & Li **sp. nov.**; from *L. meiwang* Wang & Li **sp. nov.** by the tibia spines Ⅰ strongest, tip asymmetrically bifurcated (tibia spines Ⅱ strongest in *L. meiwang* Wang & Li **sp. nov.**).

**Description. Male (holotype).** Total length 1.63 (Figure 74A). Carapace 1.05 long, 0.75 wide. Opisthosoma 0.88 long, 0.63 wide. Carapace yellow. Eyes six. Median groove needle-shaped, cervical grooves and radial furrows distinct. Clypeus 0.13 high. Opisthosoma gray, ovoid. Leg measurements: Ⅰ 6.28 (1.63, 0.25, 1.85, 1.55, 1.00); Ⅱ 4.89 (1.25, 0.25, 1.38, 1.13, 0.88); Ⅲ 4.13 (1.10, 0.23, 1.05, 1.00, 0.75); Ⅳ 5.63 (1.55, 0.25, 1.50, 1.38, 0.95). Male pedipalp (Figure 74C-D): tibia with 2 spines prolaterally and 5 spines retrolaterally, with spines Ⅰ strongest, tip asymmetrically bifurcated. Cymbium constricted medially, attaching a small earlobe-shaped process retrolaterally. Embolus triangular, prolateral lobe oval. Median apophysis short, palm-shaped, distal edge with 5 small teeth. Conductor C tile-shaped in ventral view, tip of conductor undulate (Figure 74B).

**Female (one of the paratypes).** Similar to male in color and general features, but larger and with longer legs. Total length 2.05 (Figure 75A-B). Carapace 0.90 long, 0.75 wide. Opisthosoma 1.13 long, 0.88 wide. Clypeus 0.12 high. Leg measurements: Ⅰ 6.28 (1.75, 0.25, 1.88, 1.40, 1.00); Ⅱ 4.94 (1.30, 0.25, 1.38, 1.13, 0.88); Ⅲ 4.41 (1.25, 0.23, 1.13, 1.05, 0.75); Ⅳ 5.58 (1.60, 0.25, 1.50, 1.35, 0.88). Vulva (Figure 75C): spermathecae coiled, atrium triangular.

**Distribution.** China (Hunan).

### *Leptonetela shicheng* Wang & Li sp. nov. Figures 76-77, 97

**Figure 76:**
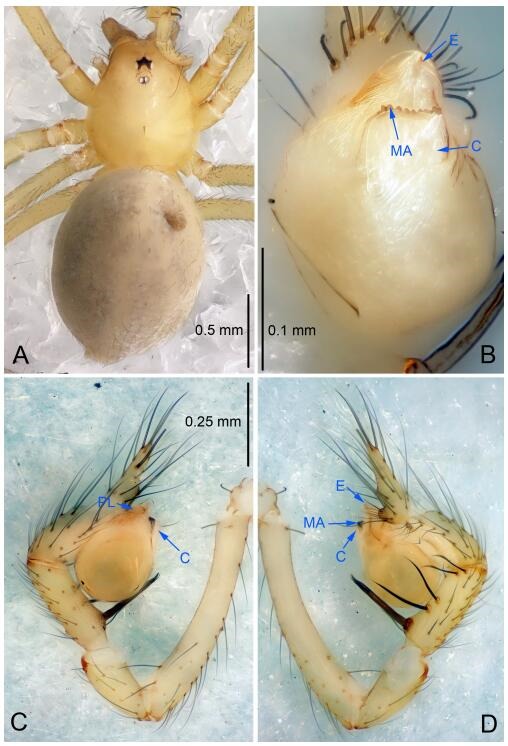
*Leptonetela shicheng* sp. nov., holotype male

**Figure 77:**
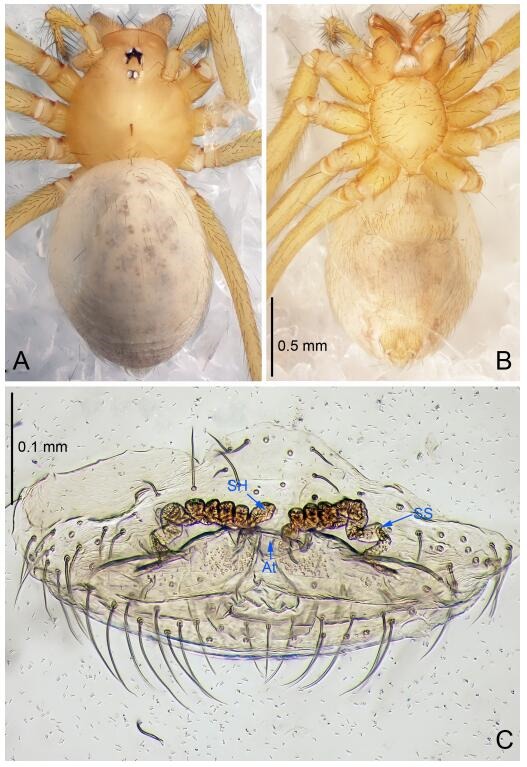
*Leptonetela shicheng* sp. nov., one of the paratype females

**Type material. Holotype:** male (IZCAS), Shicheng Cave, N27.31°, E109.07°, Jiangwu, Shanshi Town, Lianhua County, Pingxiang City, Jiangxi Province, China, 14 November 2015, Z. Chen & G. Zhou leg. **Paratypes:** 2 males and 5 females, same data as holotype.

**Etymology.** The specific name refers to the type locality; noun.

**Diagnosis.** This new species is similar to *L. sexdentata* Wang & Li, 2011, *L. longyu* Wang & Li **sp. nov.**, *L. zakou* Wang & Li **sp. nov.** and *L. meiwang* Wang & Li **sp. nov.** but can be distinguished by the harrow-like median apophysis, with 10 small teeth distally (Figure 76B) (median apophysis with 6 small teeth distally in *L. sexdentata* and *L. zakou* Wang & Li **sp. nov.**, 5 in *L. longyu* Wang & Li **sp. nov.**, and *L. meiwang* Wang & Li **sp. nov.**); conductor smooth (Figure 76B) (conductor undulate distally in *L. sexdentata*, *L. longyu* Wang & Li **sp. nov.**, and *L. zakou* Wang & Li **sp. nov.**); from *L. zakou* Wang & Li **sp. nov.** by the teeth of median apophysis needle-shaped in *L. zakou* Wang & Li **sp. nov.**; from *L. meiwang* Wang & Li **sp. nov.** by the tibia spines Ⅰ strongest, tip asymmetrically bifurcated (Figure 76D) (tibia spines Ⅱ strongest in *L. meiwang* Wang & Li **sp. nov.**).

**Description. Male (holotype).** Total length 2.40 (Figure 76A). Carapace 1.00 long, 0.73 wide. Opisthosoma 1.12 long, 0.87 wide. Carapace yellowish. Ocular area with a pair of setae, six eyes. Median groove needle-shaped, cervical grooves and radial furrows indistinct. Clypeus 0.08 high. Opisthosoma gray, ovoid. Leg measurements: Ⅰ 9.60 (2.60, 0.37, 2.50, 2.48, 1.65); Ⅱ 7.90 (2.25, 0.30, 2.25, 2.00, 1.40); Ⅲ 6.87 (1.75, 0.25, 1.87, 1.75, 1.25); Ⅳ 8.92 (2.37, 0.30, 2.50, 2.25, 1.50). Male pedipalp (Figure 76C-D): tibia with 2 long setae prolaterally, and 5 spines retrolaterally, with spines Ⅰ strongest, tip asymmetrically bifurcated. Cymbium constricted medially, attaching a small earlobe-shaped process retrolaterally. Embolus triangular, prolateral lobe indistinct. Median apophysis harrow-like, with 10 small teeth distally. Conductor smooth, C tile-shape in ventral view (Figure 76B).

**Female (one of the paratypes).** Similar to male in color and general features, but larger and with shorter legs. Total length 2.60 (Figure 77A-B). Carapace 0.87 long, 0.85 wide. Opisthosoma 1.75 long, 1.25 wide. Clypeus 0.15 high. Leg measurements: Ⅰ 8.94 (2.60, 0.37, 2.50, 2.10, 1.37); Ⅱ 7.10 (2.00, 0.30, 2.00, 1.65, 1.15); Ⅲ 5.97 (1.75, 0.25, 1.60, 1.50, 0.87); Ⅳ 7.97 (2.12, 0.35, 2.25, 2.00, 1.25). Vulva (Figure 77C): spermathecae coiled, atrium fusiform.

**Distribution.** China (Jiangxi).

### *Leptonetela zakou* Wang & Li sp. nov. Figures 78-79, 97

**Figure 78:**
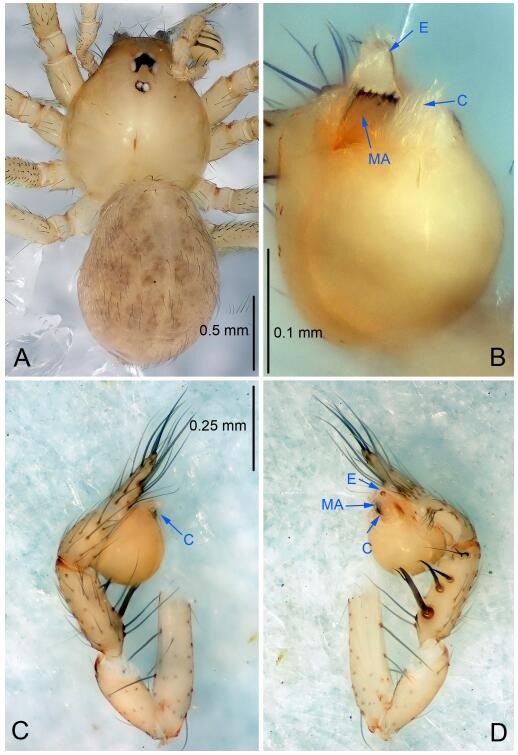
*Leptonetela zakou* sp. nov., holotype male

**Figure 79:**
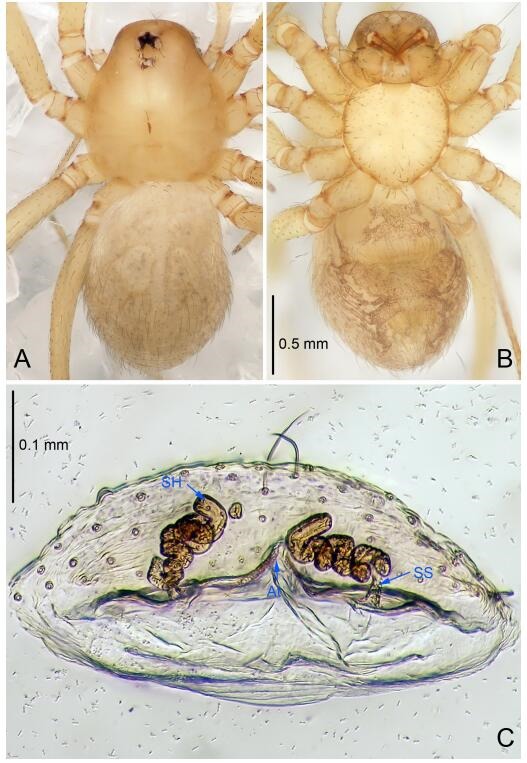
*Leptonetela zakou* sp. nov., one of the paratype females

**Type material. Holotype:** male (IZCAS), Zakou Cave, N29.35°, E109.58°, Hongyanxi Town, longshan City, Hunan Province, China, 10 January 2016, Z. Chen & Z. Wang leg. **Paratypes:** 3 males and 5 females, same data as holotype.

**Etymology.** The specific name refers to the type locality; noun.

**Diagnosis.** This new species is similar to *L. sexdentata* Wang & Li, 2011, *L. longyu* Wang & Li **sp. nov.**, *L. shicheng* Wang & Li **sp. nov.**, and *L. meiwang* Wang & Li **sp. nov.** but can be distinguished by on the male pedipalpal bulb median apophysis with 6 teeth, needle-shaped (Figure 78B) (median apophysis with 5 small teeth distally in *L. longyu* Wang & Li **sp. nov.**, 5 sharp teeth in *L. meiwang* Wang & Li **sp. nov.**, and 10 in *L. shicheng* Wang & Li **sp. nov.**, ); from *L. shicheng* Wang & Li **sp. nov.** by the distally undulate conductor (Figure 78B) (conductor smooth in *L. shicheng* Wang & Li **sp. nov.**); from *L. meiwang* Wang & Li **sp. nov.** by the tibia Ⅰ spine strongest, tip asymmetrically bifurcated (Figure 78D) (tibia Ⅱ spine strongest in *L. meiwang* Wang & Li **sp. nov.**).

**Description. Male (holotype).** Total length 1.75 (Figure 78A). Carapace 0.87 long, 0.87 wide. Opisthosoma 1.00 long, 0.87 wide. Carapace yellowish. Ocular area with a pair of setae, six eyes. Median groove needle-shaped, cervical grooves and radial furrows indistinct. Clypeus 0.08 high. Opisthosoma gray, ovoid. Leg measurements: Ⅰ 7.62 (2.00, 0.25, 2.37, 1.75, 1.25); Ⅱ 5.62 (1.50, 0.25, 1.62, 1.25, 1.00); Ⅲ 4.57 (1.25, 0.20, 1.30, 1.12, 0.70); Ⅳ 6.47 (1.87, 0.25, 1.75, 1.50, 1.10). Male pedipalp (Figure 78C-D): tibia with 3 long setae prolaterally, 5 large spines retrolaterally, with spines Ⅰ strongest, tip asymmetrically bifurcated. Cymbium not constricted, earlobe-shaped process absent. Embolus triangular, prolateral lobe absent. Median apophysis with 6 needle-shaped teeth distally. Conductor C tile-shape in ventral view (Figure 78B).

**Female (one of the paratypes).** Similar to male in color and general features, but larger and with shorter legs. Total length 1.70 (Figure 79A-B). Carapace 0.87 long, 0.80 wide. Opisthosoma 1.27 long, 0.75 wide. Clypeus 0.15 high. Leg measurements: Ⅰ 6.49 (1.75, 0.25, 1.72, 1.50, 1.27); Ⅱ 4.69 (1.37, 0.25, 1.27, 1.00, 0.80); Ⅲ 3.74 (1.12, 0.20, 1.00, 0.80, 0.62); Ⅳ 5.30 (1.50, 0.25, 1.50, 1.30, 0.75). Vulva (Figure 79C): spermathecae coiled, atrium fusiform, anterior margin of atrium with one mastoid process medially.

**Distribution.** China (Guizhou).

### *Leptonetela meiwang* Wang & Li sp. nov. Figures 80-81, 97

**Figure 80:**
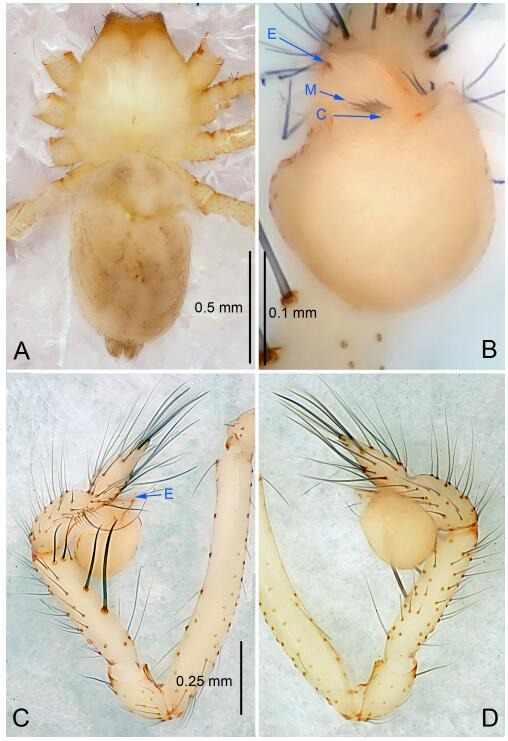
*Leptonetela meiwang* sp. nov., holotype male

**Figure 81:**
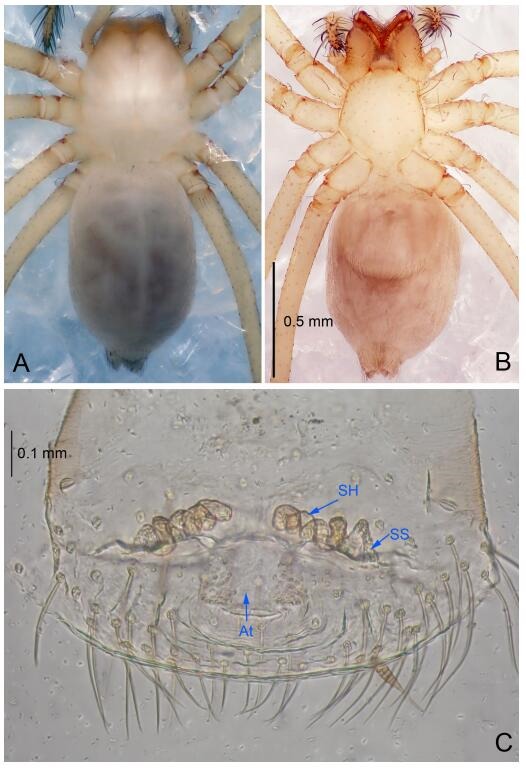
*Leptonetela meiwang* sp. nov., paratype female

**Type material. Holotype:** male (IZCAS), Meiwang Cave, N28.09°, E111.43°, Nanhua, Zhenshang Town, Lodi County, HuNan Province, China, 27 March 2016, Y. Li & Z. Chen leg. **Paratypes:** 1 male and 1 female, same data as holotype.

**Etymology.** The specific name refers to the type locality; noun.

**Diagnosis.** This new species is similar to *L. sexdentata* Wang & Li, 2011, *L. longyu* Wang & Li **sp. nov.**, *L. shicheng* Wang & Li **sp. nov.** and *L. zakou* Wang & Li **sp. nov.** but can be distinguished by the harrow-like median apophysis, with 5 sharp teeth distally (Figure 80B), tibia Ⅱ spine strongest (Figure 80C) (tibia spines Ⅰ strongest, tip asymmetrically bifurcated, median apophysis with 6 small teeth distally in *L. sexdentata* and *L. zakou* Wang & Li **sp. nov.**, 5 in *L. longyu* Wang & Li **sp. nov.**, and 10 in *L. shicheng* Wang & Li **sp. nov.**, ); conductor short, reduced (Figure 80B) (conductor broad, C shape in *L. sexdentata*, *L. longyu* Wang & Li **sp. nov.**, *L. shicheng* Wang & Li **sp. nov.** and *L. zakou* Wang & Li **sp. nov.**); from *L. zakou* Wang & Li **sp. nov.** by the teeth of median apophysis needle-shaped in *L. zakou* Wang & Li **sp. nov.**

**Description. Male (holotype):** total length 1.75 (Figure 80A). Prosoma 0.70 long, 0.62 wide. Opisthosoma 1.20 long, 0.70 wide. Carapace yellowish. Ocular area with a pair of setae, eyes absent. Median groove needle shaped, cervical grooves and radial furrows indistinct. Clypeus 0.08 high. Opisthosoma gray, ovoid. Leg measurements: Ⅰ 9.8 (2.50, 0.37, 2.81, 2.50, 1.62); Ⅱ 8.44 (2.30, 0.35, 2.30, 2.12, 1.37); Ⅲ 7.77 (2.25, 0.30, 2.12, 2.10, 1.00); Ⅳ 9.61 (2.50, 0.37, 2.50, 2.37, 1.87). Male pedipalp (Figure 80C-D): tibia with 5 spines retrolaterally, with spines Ⅱ strongest. Cymbium constricted at middle, earlobe-shaped process absent. Embolus triangular, prolateral lobe indistinct. Median apophysis with 5 sharp teeth distally. Conductor short, reduced (Figure 80B).

**Female.** Similar to male in color and general features, but larger and with shorter legs. Total length 1.75 (Figure 81A-B). Prosoma 0.75 long, 0.62 wide. Opisthosoma 1.00 long, 0.75 wide. Clypeus 0.20 high. Leg measurements: Ⅰ 8.13 (2.37, 0.34, 2.30, 1.75, 1.37); Ⅱ 7.54 (2.25, 0.34, 2.00, 1.70, 1.25); Ⅲ 6.62 (1.87, 0.30, 1.75, 1.50, 1.20); Ⅳ -(2.30, 0.34, -, -, -). Vulva (Figure 81C): spermathecae coiled, atrium triangular.

**Distribution.** China (Hunan).

### *Leptonetela tawo* Wang & Li sp. nov. Figure 82-83, 97

**Figure 82:**
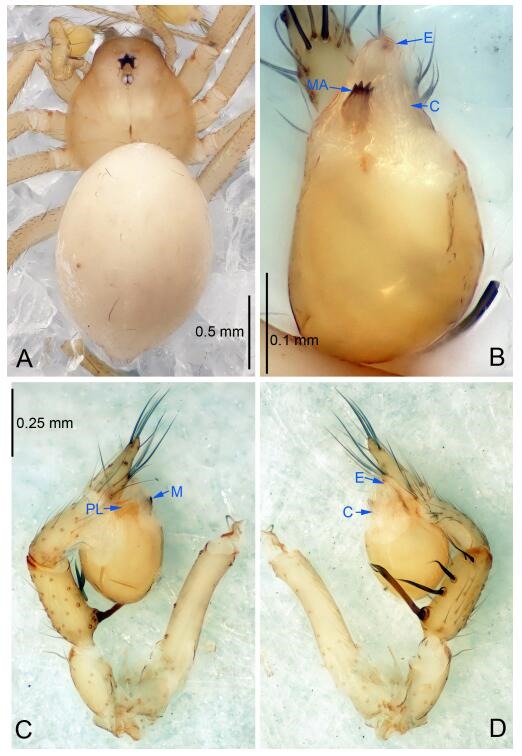
*Leptonetela tawo* sp. nov., holotype male

**Figure 83:**
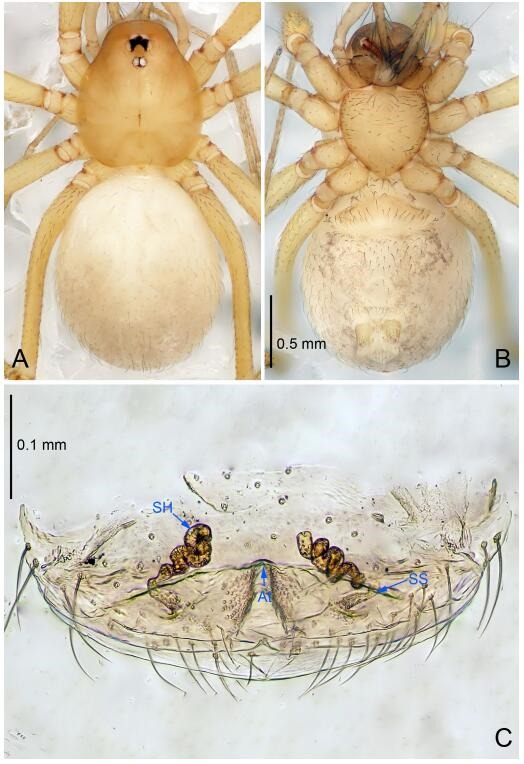
*Leptonetela tawo* sp. nov., one of the paratype females

**Type material. Holotype:** male (IZCAS), Xianren Cave, N29.18°, E109.95°, Xianren, Tawo Town, Yongshun County, Hunan Province, China, 14 January 2016, Z. Chen & Z. Wang leg. **Paratypes:** 2 males and 2 females, same data as holotype.

**Etymology.** The specific name refers to the type locality; noun.

**Diagnosis.** This new species is similar to *L. arvanitidisi* Wang & Li, 2016, *L. paragamiani* Wang & Li, 2016 and *L. erlong* Wang & Li **sp. nov.** but can be distinguished by on the male pedipalpal bulb median apophysis with 4 teeth distally (Figure 82B) (median apophysis with 6 teeth distally in *L. arvanitidisi*, 3 teeth in *L. paragamiani* and 5 teeth in *L. erlong* Wang & Li **sp. nov.**); tibia spines Ⅰ strongest, tip asymmetrically bifurcated, spines Ⅱ, Ⅲ equally strong, stronger than other 2 (Figure 82C) (tibia Ⅱ -Ⅴ spines slender, curved, and equally strong in *L. arvanitidisi* and *L. erlong* Wang & Li **sp. nov.**, tibia Ⅲ -Ⅴ spines equally strong, slender than Ⅱ spine in *L. paragamiani*); from *L. arvanitidisi* by the conductor C tile-shaped (Figure 82B) (conductor triangular in *L. arvanitidisi*).

**Description. Male (holotype).** Total length 1.90 (Figure 82A). Carapace 0.87 long, 0.75 wide. Opisthosoma 1.25 long, 0.87 wide. Carapace yellowish. Ocular area with a pair of setae, six eyes. Median groove needle-shaped, cervical grooves and radial furrows indistinct. Clypeus 0.08 high. Opisthosoma gray, ovoid. Leg measurements: Ⅰ 7.69 (2.00, 0.35, 2.37, 1.72, 1.25); Ⅱ 5.95 (1.75, 0.30, 1.55, 1.35, 1.00); Ⅲ 4.60 (1.25, 0.25, 1.15, 1.10, 0.85); Ⅳ 7.15 (1.85, 0.30, 2.25, 1.60, 1.15). Male pedipalp (Figure 82C-D): tibia with 4 long setae prolaterally, 5 large spines retrolaterally, with spines Ⅰ strongest, tip asymmetrically bifurcated, tibia spines Ⅱ, Ⅲ equally strong, stronger than other 2. Cymbium not constricted, earlobe-shaped process absent. Embolus triangular, prolateral lobe indistinct. Median apophysis with 4 teeth distally. Conductor C tile-shaped in ventral view (Figure 82B).

**Female (one of the paratypes).** Similar to male in color and general features, but larger and with shorter legs. Total length 2.00 (Figure 83A-B). Carapace 0.87 long, 0.75 wide. Opisthosoma 1.12 long, 1.00 wide. Clypeus 0.15 high. Leg measurements: Ⅰ 5.84 (1.62, 0.35, 1.62, 1.25, 1.00); Ⅱ 4.55 (1.25, 0.35, 1.20, 1.00, 0.75); Ⅲ 3.62 (1.00, 0.30, 0.87, 0.85, 0.60); Ⅳ 5.42 (1.75, 0.35, 1.37, 1.10, 0.85). Vulva (Figure 83C): spermathecae coiled, atrium triangular.

**Distribution.** China (Guizhou).

### *Leptonetela erlong* Wang & Li sp. nov. Figures 84-85, 97

**Figure 84:**
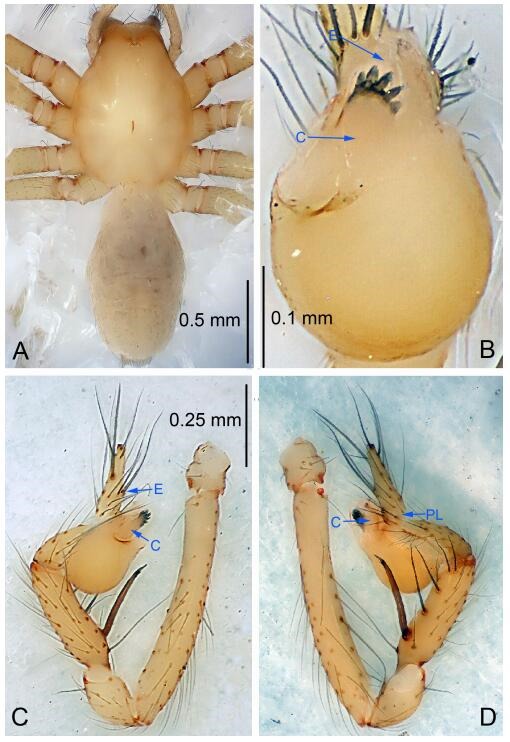
*Leptonetela erlong* sp. nov., holotype male

**Figure 85:**
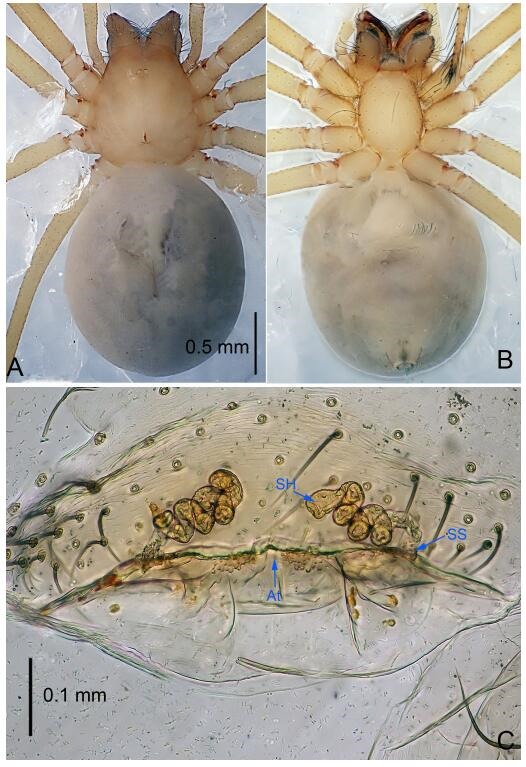
*Leptonetela erlong* sp. nov., one of the paratype females

**Type material. Holotype:** male (IZCAS), Erlong Cave, N27.82°, E110.23°, Siqian Town, Chenxi County, Huaihua City, Hunan Province, China, 19 March 2016, Y. Li & Z. Chen leg. **Paratypes:** 4 males and 2 females, same data as holotype.

**Etymology.** The specific name refers to the type locality; noun.

**Diagnosis.** This new species is similar to *L. arvanitidisi* Wang & Li, 2016, *L. paragamiani* Wang & Li, 2016 and *L. tawo* Wang & Li **sp. nov.** but can be distinguished by on the male pedipalpal bulb median apophysis with 5 teeth distally (Figure 84B) (median apophysis with 6 teeth in *L. arvanitidisi*, 4 teeth in *L. tawo* Wang & Li **sp. nov.** and 3 teeth *L. paragamiani*); from *L. paragamiani* and *L. tawo* Wang & Li **sp. nov.** by the tibia spines Ⅱ -Ⅴ slender, curved, and equally strong (Figure 84D) (tibia spines Ⅱ, Ⅲ equally strong, stronger than other 2 in *L. tawo* Wang & Li **sp. nov.**, spines Ⅲ -Ⅴ equally strong, more slender than spines Ⅱ in *L. paragamiani*); from *L. arvanitidisi* by the conductor C tile-shaped (Figure 84B) (conductor triangular in *L. arvanitidisi*).

**Description. Male (holotype):** total length 1.95 (Figure 84A). Prosoma 0.50 long, 0.80 wide. Opisthosoma 1.45 long, 1.00 wide. Prosoma yellowish. Eyes absent. Median groove needle-shaped, brown. Cervical grooves and radial furrows indistinct. Clypeus 0.13 high, slightly sloped anteriorly. Opisthosoma pale brown, ovoid, covered with short hairs, lacking distinct pattern. Sternum and legs yellowish. Leg measurements: Ⅰ 6.81 (2.35, 0.35, 1.87, 1.37, 0.87); Ⅱ 5.82 (2.25, 0.35, 1.35, 1.07, 0.80); Ⅲ 5.22 (2.20, 0.30, 1.00, 0.97, 0.75); Ⅳ 6.30 (2.30, 0.35, 1.50, 1.30, 0.85). Male pedipalp (Figure 84C-D): tibia with 5 spines retrolaterally, with spines Ⅰ strongest, tip asymmetrically bifurcated, tibia spines Ⅱ -Ⅴ slender, curved, and equally strong. Cymbium constricted medially, earlobe-shaped process small. Embolus triangular, prolateral lobe indistinct. Median apophysis with 5 teeth distally. Conductor C tile-shaped in ventral view (Figure 84B).

**Female (one of the paratypes).** Similar to male in color and general features, but larger and with shorter legs. Total length 2.30 (Figure 85A-B). Prosoma 0.85 long, 0.95 wide. Opisthosoma 0.87 long, 0.70 wide. Clypeus 0.20 high. Leg measurements: Ⅰ 8.60 (2.50, 0.35, 2.20, 1.80, 1.75); Ⅱ 7.65 (2.35, 0.30, 1.95, 1.70, 1.35); Ⅲ 6.30 (2.15, 0.25, 2.05, 1.15, 0.70); Ⅳ 7.85 (2.25, 0.30, 2.05, 1.75, 1.50). Vulva (Figure 85C): spermathecae coiled, atrium fusiform.

**Distribution.** China (Hunan).

### *Leptonetela dabian* Wang & Li sp. nov. Figures 86-87, 97

**Figure 86:**
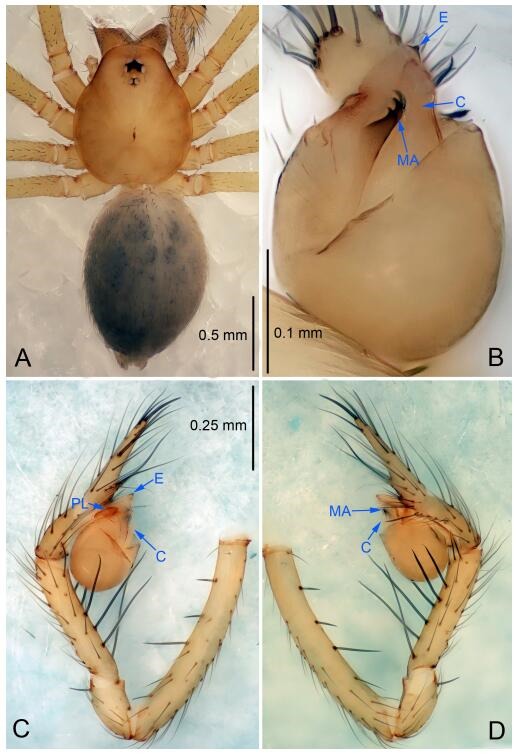
*Leptonetela dabian* sp. nov., holotype male

**Figure 87:**
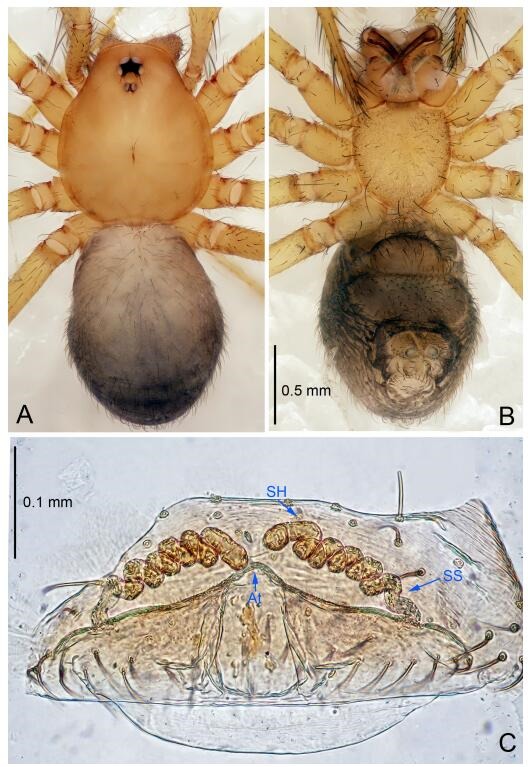
*Leptonetela dabian* sp. nov., one of the paratype females

**Type material. Holotype:** male (IZCAS), Wuming Cave, N25.75°, E107.92°, Dabian, Sandong Town, Sandu County, Qiannan Prefecture, Guizhou, China, 22 March 2013, H. Zhao & J. Liu leg. **Paratypes:** 2 females, same data as holotype.

**Etymology.** The specific name refers to the type locality; noun.

**Diagnosis.** This new species is similar to *L. thracia* Wang & Li, 2011 and *L. chuan* Wang & Li **sp. nov.**, but can be distinguished by the male pedipalal tibia with 3 spines prolaterally, 5 slender spines, retrolaterally (Figure 86C-D) (tibia with 4 long setae prolaterally, 5 large spines retrolaterally, spines Ⅰ, Ⅱ equally strong, stronger than others in *L. thracia*; tibia with 7 long setae prolaterally, 5 large spines retrolaterally, spines Ⅰ, Ⅱ, Ⅲ equally strong, stronger than others in *L. chuan* Wang & Li **sp. nov.**); tip of median apophysis bent upwards, with 3 larger teeth distally (Figure 86B) (tip of median apophysis bent downwards, with 5 larger teeth distally in *L. chuan* Wang & Li **sp. nov.**; tip of median apophysis not bent, with 4 teeth distally in *L. thracia*); conductor thin, tongue shaped (Figure 86B) (conductor triangular in *L. thracia* and *L. chuan* Wang & Li **sp. nov.**).

**Description. Male (holotype).** Total length 2.38 (Figure 86A). Carapace 1.00 long, 0.80 wide. Opisthosoma 1.25 long, 0.90 wide. Carapace yellow. Six eyes. Median groove needle-shaped, cervical grooves and radial furrows indistinct. Clypeus 0.13 high. Opisthosoma gray, ovoid, with pigmented spots. Leg measurements: Ⅰ -(2.60, 0.38, 2.35, -, -); Ⅱ 7.78 (2.15, 0.38, 2.25, 1.75, 1.25); Ⅲ -(1.88, 0.35, 1.75, -, -); Ⅳ 8.26 (2.25, 0.38, 2.38, 2.00, 1.25). Male pedipalp (Figure 86C-D): tibia with 3 slender spines prolaterally, 5 large retrolateral spines equally strong. Cymbium constricted medially, attaching a small earlobe-shaped process retrolaterally. Embolus triangular, prolateral lobe oval. Tip of median apophysis bent upward, distal edge decorated with three small teeth. Conductor thin, tongue-shaped (Figure 86B).

**Female (one of the paratypes).** Similar to male in color and general features, but larger and with shorter legs. Total length 2.40 (Figure 87A-B). Carapace 1.00 long, 0.88 wide. Opisthosoma 1.25 long, 1.00 wide. Clypeus 0.13 high. Leg measurements: Ⅰ 9.51 (2.50, 0.38, 3.00, 2.13, 1.50); Ⅱ 7.38 (2.00, 0.38, 2.13, 1.62, 1.25); Ⅲ 6.35 (1.75, 0.35, 1.75, 1.50, 1.00); Ⅳ 8.06 (2.25, 0.38, 2.30, 1.88, 1.25). Vulva (Figure 87C): spermathecae coiled, atrium triangular, and anterior margin of atrium with short hairs.

**Distribution.** China (Guizhou).

### *Leptonetela chuan* Wang & Li sp. nov. Figures 88-89, 97

**Figure 88:**
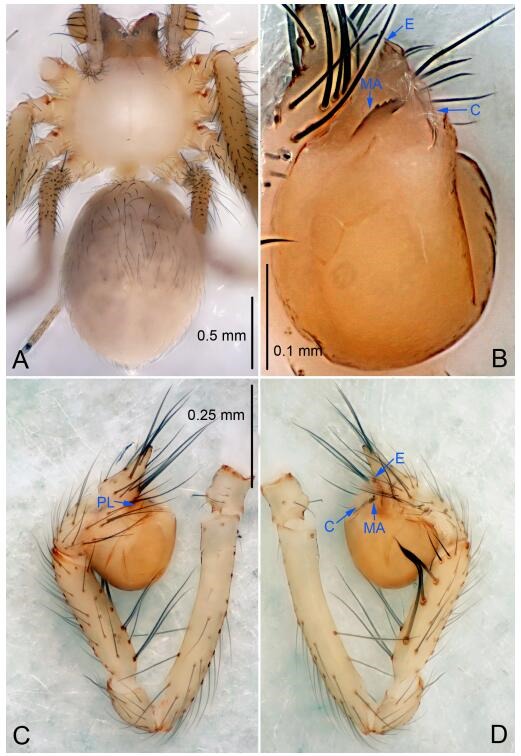
*Leptonetela chuan* sp. nov., holotype male

**Figure 89:**
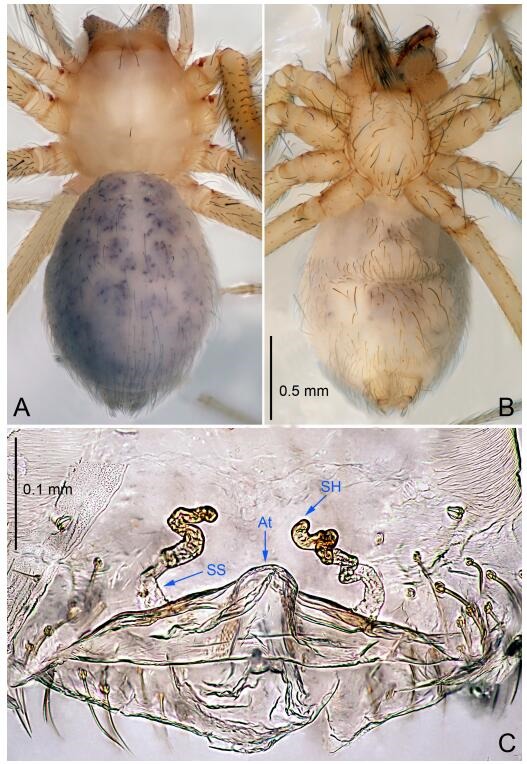
*Leptonetela chuan* sp. nov., one of the paratype females

**Type material. Holotype:** male (IZCAS), Chuan Cave, N27.08°, E105.67°, Yangchangba Town, Dafang County, Guizhou Province, China, 13 March 2011, H. Chen & Z. Zha leg. **Paratype:** 1 female, same data as holotype.

**Etymology.** The specific name refers to the type locality; noun.

**Diagnosis.** This new species is similar to *L. thracia* Wang & Li, 2011 and *L. dabian* Wang & Li **sp. nov.**, but can be distinguished by the male pedipalpal tibia with 7 long setae prolaterally, 5 slender spines retrolaterally, with spines Ⅰ, Ⅱ, Ⅲ equally strong, stronger than others (Figure 88D) (tibia with 4 long setae prolaterally, 5 slender spines retrolaterally, with spines Ⅰ, Ⅱ equally strong, stronger than others in *L. thracia*; 3 slender spines prolaterally, 5 slender retrolaterally spines equally strong in *L. dabian* Wang & Li **sp. nov.**); tip of median apophysis bent downwards, with 5 larger teeth distally (Figure 88B) (tip of median apophysis not bent, with 4 teeth distally in *L. thracia*; tip of median apophysis bent upwards, with 3 larger teeth distally in *L. dabian* Wang & Li **sp. nov.**); from *L. dabian* Wang & Li **sp. nov.** by the triangular conductor (Figure 88B) (conductor thin, tongue-shaped in *L. dabian* Wang & Li **sp. nov.**).

**Description. Male (holotype).** Total length 2.10 (Figure 88A). Carapace 0.83 long, 0.90 wide. Opisthosoma 1.18 long, 1.05 wide. Carapace whitish. Ocular area with a pair of setae, eyes absent. Median groove, cervical grooves and radial furrows indistinct. Clypeus 0.13 high. Opisthosoma gray, ovoid. Leg measurements: Ⅰ 8.79 (2.38, 0.38, 2.50, 2.13, 1.40); Ⅱ 7.77 (2.13, 0.38, 2.18, 1.78, 1.30); Ⅲ 7.51 (1.78, 0.35, 2.10, 1.73, 1.55); Ⅳ 8.36 (2.25, 0.38, 2.38, 2.00, 1.35). Male pedipalp (Figure 88C-D): tibia with 7 long setae prolaterally, 5 large spines retrolaterally, with spines Ⅰ, Ⅱ, Ⅲ equally strong, stronger than others. Cymbium constricted medially, attached to a small earlobe-shaped process retrolaterally. Embolus triangular, prolateral lobe oval. Median apophysis bent downwards, with 5 larger teeth distally. Conductor triangular in ventral view (Figure 88B).

**Female:** Similar to male in color and general features, but smaller and with shorter legs. Total length 2.08 (Figure 89A-B). Carapace 0.78 long, 0.88 wide. Opisthosoma 1.33 long, 1.03 wide. Clypeus 0.15 high. Leg measurements: Ⅰ 8.31 (2.33, 0.38, 2.40, 1.85, 1.35); Ⅱ 7.21 (2.10, 0.35, 2.08, 1.58, 1.10); Ⅲ 6.92 (1.93, 0.35, 1.88, 1.63, 1.13); Ⅳ 8.14 (2.30, 0.38, 2.38, 1.83, 1.25). Vulva (Figure 89C): spermathecae loosely coiled, atrium triangular.

**Distribution.** China (Guizhou).

### *Leptonetela lihu* Wang & Li sp. nov. Figures 90-91, 97

**Figure 90:**
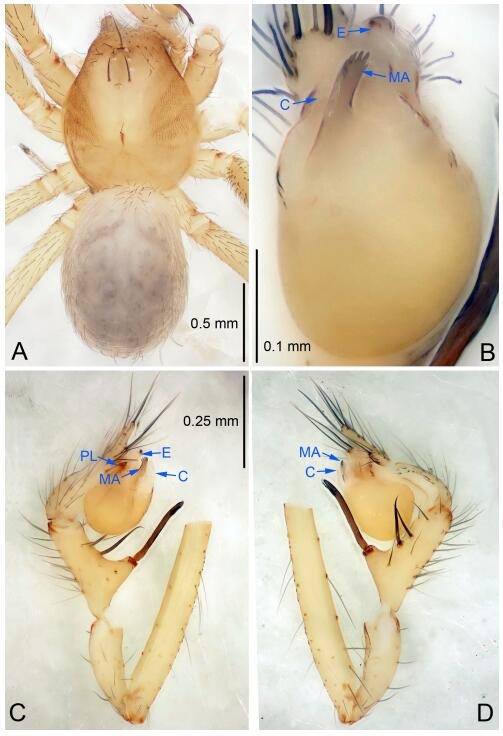
*Leptonetela lihu* sp. nov., holotype male

**Figure 91:**
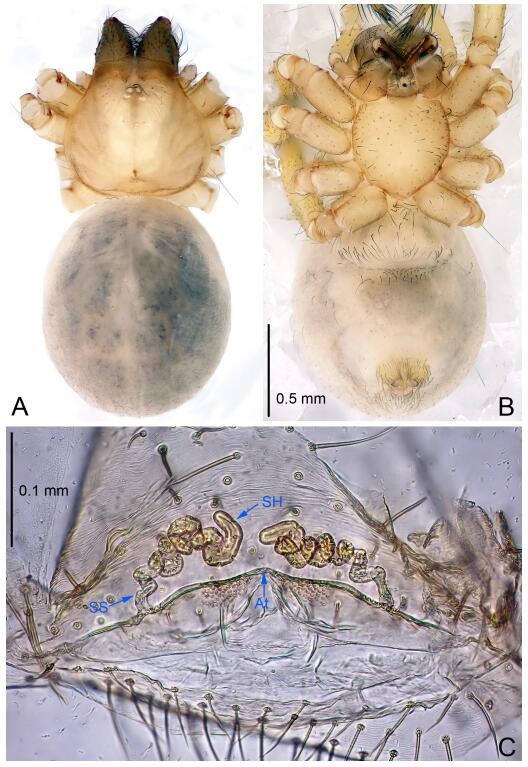
*Leptonetela lihu* sp. nov., one of the paratype females

**Type material. Holotype:** male (IZCAS), nameless Cave, N25.10°, E107.65°, Lihu Town, Nandan County, Hechi City, Guangxi Zhuang Autonomous Region, China, 31 January 2015, Y. Li & Z. Chen leg. **Paratypes:** 2 males and 5 females, same data as holotype.

**Etymology.** The specific name refers to the type locality; noun.

**Diagnosis.** This new species is similar to *L. notabilis* (Lin & Li, 2010), *L. sexdigiti* (Lin & Li, 2010); and *L. shuang* Wang & Li **sp. nov.**, but can be separated from *L. notabilis* by the male pedipalpal tibia spines Ⅰ bifurcate (Figure 90D) (tibia spines Ⅰ trifurcate in *L. notabilis*); from *L. shuang* Wang & Li **sp. nov.** by the conductor C tile-shaped, distal edge of median apophysis with 6 teeth (Figure 90B) (conductor triangular, distal edge of median apophysis with 7 teeth in *L. shuang* Wang & Li **sp. nov.**); from *L. sexdigiti* by the strongly twisted spermathecae (spermathecae loosely twisted in *L. sexdigiti*).

**Description. Male (holotype).** Total length 2.13 (Figure 90A). Carapace 1.00 long, 0.88 wide. Opisthosoma 1.12 long, 0.75 wide. Carapace yellowish. Ocular area with 3 long setae, six eyes, reduced to white spots. Median groove needle-shaped, cervical grooves and radial furrows distinct. Clypeus 0.13 high. Opisthosoma gray, ovoid. Leg measurements: Ⅰ 8.23 (2.25, 0.25, 2.38, 1.95, 1.40); Ⅱ 7.00 (2.00, 0.25, 2.00, 1.63, 1.12); Ⅲ 6.10 (1.75, 0.20, 1.75, 1.45, 0.95); Ⅳ 7.75 (2.10, 0.25, 2.25, 1.90, 1.25). Male pedipalp (Figure 90C-D): basal part of tibia swollen, tibia with 5 spines retrolaterally, with spines Ⅰ strongest, longest, bifurcate and located at the base of tibia. Cymbium constricted medially, attached to a small earlobe-shaped process retrolaterally. Embolus triangular, prolateral lobe oval. Median apophysis long and thin, with 6 small teeth distally. Conductor broad, C tile-shaped in ventral view (Figure 90B).

**Female (one of the paratypes).** Similar to male in color and general features, but larger and with shorter legs. Total length 2.50 (Figure 91A-B). Carapace 1.25 long, 0.75 wide. Opisthosoma 1.40 long, 1.00 wide. Clypeus 0.12 high. Leg measurements: Ⅰ 8.00 (2.25, 0.25, 2.38, 1.75, 1.37); Ⅱ 7.00 (2.00, 0.25, 2.00, 1.50, 1.25); Ⅲ 5.65 (1.75, 0.20, 1.65, 1.15, 0.90); Ⅳ 7.70 (2.10, 0.25, 2.25, 1.90, 1.20). Vulva (Figure 91C): spermathecae coiled, atrium fusiform.

**Distribution.** China (Guangxi).

### *Leptonetela shuang* Wang & Li sp. nov. Figures 92-93, 97

**Figure 92:**
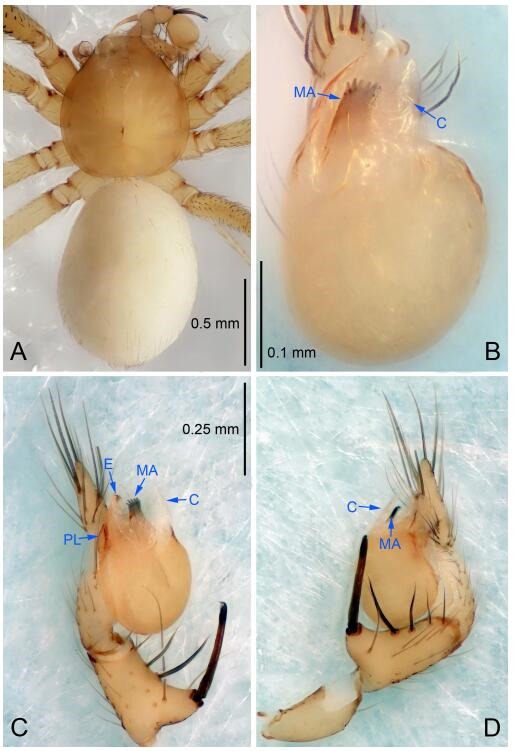
*Leptonetela shuang* sp. nov., holotype male

**Figure 93:**
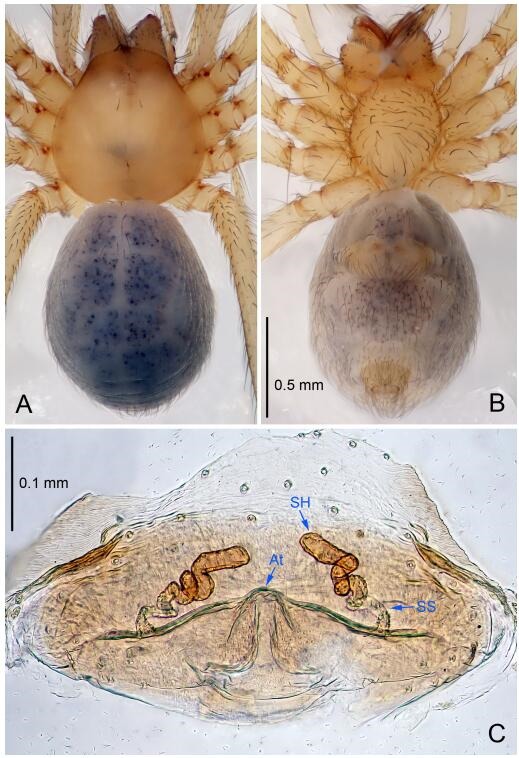
*Leptonetela shuang* sp. nov., one of the paratype females

**Type material. Holotype:** male (IZCAS), Shuang Cave, N25.93°, E107.26°, Bailong Town, Pingtang County, Qiannan Prefecture, Guizhou Province, China, 24 July 2012, H. Zhao leg. **Paratypes:** 2 females, same data as holotype; 2 males and 6 females, Dongkou Cave, N25.93°, E107.25°, Longxiang, Bailong Town, Pingtang County, Qiannan Prefecture, Guizhou Province, China, 25 July 2012, H. Zhao leg.

**Etymology.** The specific name refers to the type locality; noun.

**Diagnosis.** This new species is similar to *L. notabilis* (Lin & Li, 2010), *L. sexdigiti* (Lin & Li, 2010), and *L. lihu* Wang & Li **sp. nov.**, but can be separated from *L. notabilis* by the male pedipalp tibia spines Ⅰ bifurcate (Figure 92D) (tibia spines Ⅰ trifurcate in *L. notabilis*); from *L. sexdigiti* and *L. lihu* Wang & Li **sp. nov.** by the conductor triangular, distal edge of median apophysis with 7 teeth (Figure 92B) (conductor C tile-shaped, distal edge of median apophysis with 6 teeth in *L. sexdigiti* and *L. lihu* Wang & Li **sp. nov.**); from *L. sexdigiti* by in the female spermathecae strongly twisted (Figure 93C) (spermathecae loosely twisted in *L. sexdigiti*).

**Description. Male (holotype).** Total length 2.00 (Figure 92A). Carapace 0.83 long, 0.75 wide. Opisthosoma 1.25 long, 0.80 wide. Carapace yellow. Ocular area with a pair of setae, eyes absent. Median groove needle-shaped, cervical grooves and radial furrows indistinct. Clypeus 0.13 high. Opisthosoma whitish, ovoid. Leg measurements: Ⅰ 7.74 (2.03, 0.38, 2.25, 1.80, 1.28); Ⅱ 6.65 (1.77, 0.35, 1.83, 1.50, 1.20); Ⅲ 5.68 (1.57, 0.35, 1.50, 1.28, 0.98); Ⅳ 7.38 (1.92, 0.38, 2.05, 1.78, 1.25). Male pedipalp (Figure 92C-D): basal of tibia swollen, tibia with 3 long setae prolaterally, 1 long setae and 5 spines retrolaterally, with spines Ⅰ strongest, longest, bifurcate and located at the base of the tibia. Cymbium constricted medially, attached to a small earlobe-shaped process retrolaterally. Embolus triangular, prolateral lobe oval. Median apophysis long and thin, with 7 small teeth distally. Conductor triangular (Figure 92B).

**Female (one of the paratypes).** Similar to male in color and general features, but smaller and with shorter legs. Total length 1.98 (Figure 93A-B). Carapace 0.88 long, 0.75 wide. Opisthosoma 1.13 long, 0.88 wide. Clypeus 0.13 high. Leg measurements: Ⅰ 7.34 (1.93, 0.38, 2.13, 1.65, 1.25); Ⅱ 6.41 (1.75, 0.35, 1.78, 1.50, 1.03); Ⅲ 5.50 (1.52, 0.35, 1.45, 1.30, 0.88); Ⅳ 7.03 (1.87, 0.38, 2.05, 1.63, 1.10). Vulva (Figure 93C): spermathecae coiled, atrium triangular.

**Distribution.** China (Guizhou).

### *Leptonetela encun* Wang & Li sp. nov. Figures 94-95, 97

**Figure 94:**
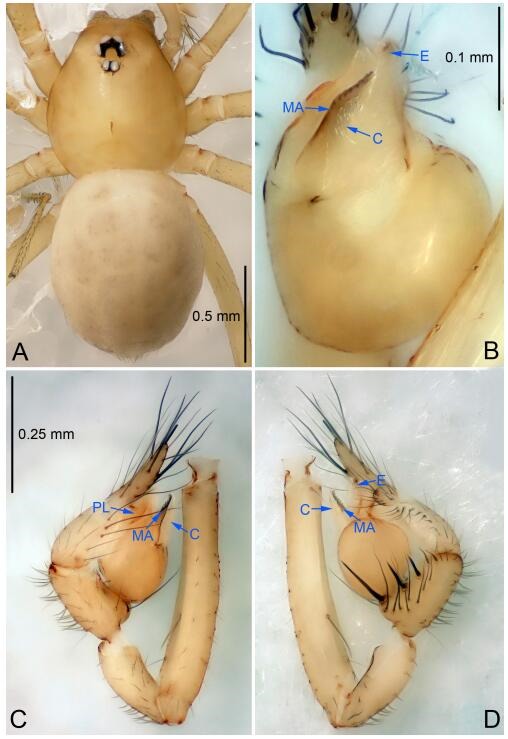
*Leptonetela encun* sp. nov., holotype male

**Figure 95:**
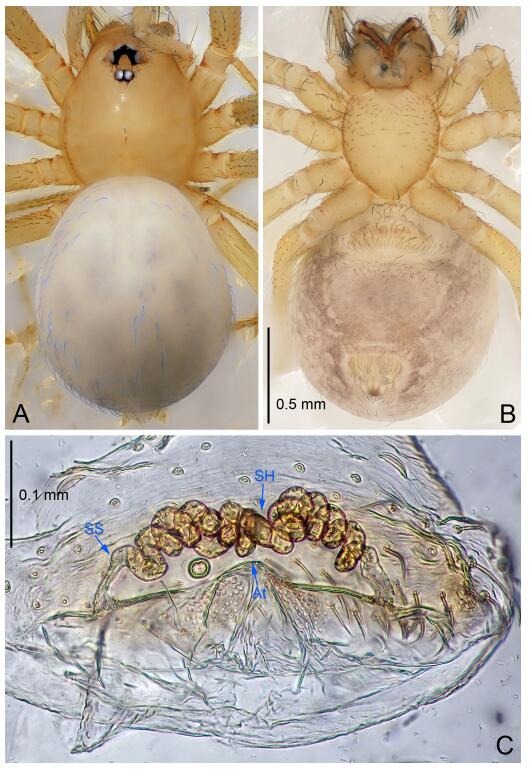
*Leptonetela encun* sp. nov., paratype female

**Type material. Holotype:** male (IZCAS), Encun Cave, N25.08°, E107.59°, En, Chengguan Town, Nandan County, Hechi City, Guangxi Zhuang Autonomous Region, China, 30 January 2015, Y. Li & Z. Chen leg. **Paratypes:** 1 male and 1 female, same data as holotype.

**Etymology.** The specific name refers to the type locality; noun.

**Diagnosis.** This new species is similar to *L. robustispina* (Chen et al, 2010) but can be distinguished by the male pedipalpal tibia with 5 spines retrolaterally, with spines Ⅰ longest, spines Ⅰ, Ⅱ, Ⅲ equally strong, stronger than others (Figure 94D), distal edge of median apophysis linear, with 8 teeth (Figure 94B) (tibia with 5 spines retrolaterally, with spines Ⅰ longest, distal edge of median apophysis semicircular, with 12 teeth in *L. robustispina*).

**Description. Male (holotype).** Total length 2.00 (Figure 94A). Carapace 0.90 long, 0.75 wide. Opisthosoma 1.25 long, 0.88 wide. Carapace yellowish, with one seta on the median part. Six eyes. Median groove needle-shaped, cervical grooves and radial furrows indistinct. Clypeus 0.10 high. Opisthosoma gray, ovoid. Leg measurements: Ⅰ -(1.88, -, -, -, -); Ⅱ 6.25 (1.75, 0.25, 1.87, 1.38, 1.00); Ⅲ 4.96 (1.38, 0.20, 1.38, 1.20, 0.80); Ⅳ 6.86 (2.00, 0.25, 1.88, 1.63, 1.10). Male pedipalp (Figure 94C-D): tibia with a few clusters of short spines dorsally, 8 long setae retrolaterally, and 5 spines retrolaterally, spines Ⅰ longest. Cymbium constricted medially, attached to a small earlobe-shaped process retrolaterally. Embolus triangular, prolateral lobe indistinct. Median apophysis harrow-like, distal edge round, with 8 small teeth. Conductor triangular in ventral view (Figure 94B).

**Female:** Similar to male in color and general features, but larger and with shorter legs. Total length 2.13 (Figure 95A-B). Carapace 0.88 long, 0.75 wide. Opisthosoma 1.00 long, 1.05 wide. Clypeus 0.11 high. Leg measurements: Ⅰ 6.50 (1.75, 0.25, 2.00, 1.50, 1.00); Ⅱ 5.01 (1.38, 0.25, 1.50, 1.13, 0.75); Ⅲ 4.45 (1.25, 0.20, 1.25, 1.00, 0.75); Ⅳ 5.52 (1.50, 0.25, 1.62, 1.25, 0.90). Vulva (Figure 95C): spermathecae coiled, atrium fusiform.

**Distribution.** China (Guangxi).

### *Leptonetela zhai* Wang & Li, 2011 Figures 96-97

**Figure 96:**
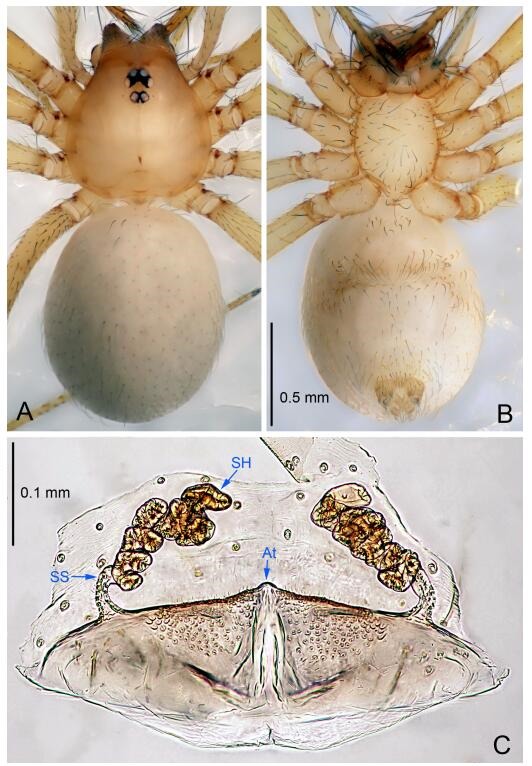
*Leptonetela zhai* Wang & Li, 2011, one female from the type locality

*Leptonetela zhai* Wang & Li, 2011: 17, Figures 69A-D, 70A-B, 71C-D.

**Material examined.** 4 females (IZCAS), Rudong Cave, N25.57°, E110.62°, Longpan Mountain, Dongtian, Hucheng Town, Xing'an County, Guilin City, Guangxi Zhuang Autonomous Region, China, 08 November 2012, Z. Chen & Z. Zhao leg.

**Description**. Male. See Wang & Li (2011).

**Female**. Total length 2.12 (Figure 96A-B). Carapace 0.80 long, 0.73 wide. Opisthosoma 1.27 long, 0.85 wide. Clypeus 0.12 high. Leg measurements: Ⅰ 6.61 (1.62, 0.37, 1.65, 1.87, 1.10); Ⅱ 5.39 (1.77, 0.30, 1.25, 1.12, 0.95); Ⅲ 4.16 (1.12, 0.27, 1.02, 1.00, 0.75); Ⅳ 5.72 (1.50, 0.30, 1.55, 1.35, 1.02). Vulva (Figure 96C): spermathecae coiled, atrium fusiform, anterior margin of atrium with short hairs.

**Distribution**. China (Guangxi).

**Remarks**. The female of the species is described for the first time. Females of *Leptonetela* *zhai* were collected from the same cave where the male holotype of *L. zhai* Wang & Li, 2011 was found.

### *Leptonetela tianxinensis* (Tong & Li, 2008) comb. nov.

*Leptoneta tianxinensis* Tong & Li, 2008: 382, Figures 5A-G (♂♀).

**Type material examined.** Paratypes: 12 males, 6 females (IZCAS), Tianxin Cave, N33.35°, E111.88°, Sandaohe, Qilipo Town, Neixiang County, Henan Province, China, 24 June 2005, Q. Wang & Y. Tong leg.

**Remarks**. Our research showed that this species should be transferred to the genus *Leptonetela*, based on the result of DNA barcoding and morphological characters such as the pedipalpal femur lacking spines and the tibia with one strong spine retrolaterally.

### *Leptonetela gigachela* (Lin & Li, 2010) comb. nov.

*Guineta gigachela* Lin & Li, 2010: 6, Figures 1A, 2A- E, 3A- B (♂♀).

**Type material examined. Holotype:** male (IZCAS), Qingzi Cave, N26.51°, E107.99°, Mianxi, Sankeshu Town, Kaili City, Guizhou Province, China, 26 May 2007, Y. Li & J. Liu leg. **Paratypes:** 2 males and 12 females, same data as holotype.

**Remarks**. Our studies showed that that *Guineta* Lin & Li, 2010 syn. nov. should be a junior synonym of *Leptonetela* Kratochvíl, 1978.

### *Leptonetela notabilis* (Lin & Li, 2010) comb. nov.

*Sinoneta notabilis* Lin & Li, 2010: 83, Figures 55A-B, 56A-C, 57A-C (♂♀).

**Type material examined. Holotype:** male (IZCAS), Hebiandong Cave, Kaikou Town, Duyun City, N26.00°, E107.20°, Guizhou Province, China, 8 May 2006, Y. Li leg. **Paratypes:** 1 male and 1 female, same data as holotype.

**Remarks**. Our studies showed that *Sinoneta* Lin & Li, 2010 syn. nov. should be a junior synonym of *Leptonetela* Kratochvíl, 1978.

### *Leptonetela sexdigiti* (Lin & Li, 2010) comb. nov.

*Sinoneta sexdigiti* Lin & Li, 2010: 87, Figures 58A-B, 59A-B, 60A-B (♂♀).

**Type material examined. Holotype:** male (IZCAS), Qiaotou Cave, Dashan, Shuangliu Town, Kaiyang County, N26.05°, E107.85°, Guizhou Province, China, 11 May 2006, Y. Li & Z. Yang leg. **Paratypes:** 5 males and 29 females, same data as holotype.

### *Leptonetela sanchahe* Wang & Li nom. nov.

*Qianleptoneta palmata* Chen, Jia & Wang, 2010: 2902, Figures 19A-G, 20A-F, 25G (♂♀).

*Sinoneta palmata* Wang & Li, 2011: 4 (Transfer from *Qianleptoneta*).

**Material examined.** 1 male and 1 female (IZCAS), Sanchahe Cave, N26.53°, E107.70°, Sanchahe, Jialiang Town, Libo County, Guizhou Province, China, 16 May 2011, C. Wang & L. Lin leg.

**Etymology.** The specific name refers to the type locality; noun.

**Remarks**. *Qianleptoneta palmata* was collected from Sanchahe Cave in Guizhou, China and published by Chen et al. in December 2010. Wang & Li (2011) transfered *Qianleptoneta palmata* Chen et al, 2010 to the genus *Sinoneta* Lin & Li, 2010. Nevertheless, in this study our results confirmed that *Qianleptoneta palmata* belonged to the genus *Leptonetela.*

*Leptonetela palmata* is a preoccupied name (secondary homonym) for a species collected from Dixian Cave in Guizhou, China and published by Lin & Li, in August 2010. Subsequently, *Leptonetela sanchahe* Wang & Li **nom. nov.** is proposed for the taxon from Sanchahe Cave, in Guizhou, China.

## ACKNOWLEDGEMENTS

Yi Wu and Guo Zheng helped to prepare photos of the manuscript. Sarah C. Crews kindly checked the English of the manuscript. Peter Jäger, Yanfeng Tong and Yucheng Lin provided valuable comments on an early version of the manuscript.
